# Comparative genomic analyses of *Streptococcus mutans *provide insights into chromosomal shuffling and species-specific content

**DOI:** 10.1186/1471-2164-10-358

**Published:** 2009-08-05

**Authors:** Fumito Maruyama, Mitsuhiko Kobata, Ken Kurokawa, Keishin Nishida, Atsuo Sakurai, Kazuhiko Nakano, Ryota Nomura, Shigetada Kawabata, Takashi Ooshima, Kenta Nakai, Masahira Hattori, Shigeyuki Hamada, Ichiro Nakagawa

**Affiliations:** 1 Division of Bacteriology, Department of Infectious Diseases Control, International Research Center for Infectious Diseases, The Institute of Medical Science, The University of Tokyo, Tokyo 108-8639, Japan; 2 Department of Pediatric Dentistry, Osaka University Graduate School of Dentistry, 1-8 Yamada-oka, Suita, Osaka 565-0871, Japan; 3 Divison of Information Biotechnology, Department of Bioinformation Engineering, Tokyo Institute of Technology School and Graduate School of Bioscience and Biotechnology, 4259 Nagatsuta-cho, Midori-ku, Yokohama Kanagawa 226-8501, Japan; 4 Human genome Center, Institute of Medical Science, The University of Tokyo; 5 Department of Oral and Molecular Microbiology, Osaka University Graduate School of Dentistry, Suita, Osaka, 565-0871, Japan; 6 Department of Computational Biology, Graduate School of Frontier Sciences, The University of Tokyo, 5-1-5 Kashiwanoha, Kashiwa, Chiba 277-8561, Japan; 7 Research Collaboration Center on Emerging and Reemerging Infections (RCC-ERI) 6F, Department of Medical Sciences, Ministry of Public Health, Tiwanon Road, Muang Nonthaburi, 11000, Thailand; 8 Section of Bacterial Pathogenesis, Graduate School of Medical and Dental Sciences, Tokyo Medical and Dental University, 1-5-45, Yushima, Bunkyo-ku, Tokyo 113-8510, Japan

## Abstract

**Background:**

*Streptococcus mutans *is the major pathogen of dental caries, and it occasionally causes infective endocarditis. While the pathogenicity of this species is distinct from other human pathogenic streptococci, the species-specific evolution of the genus *Streptococcus *and its genomic diversity are poorly understood.

**Results:**

We have sequenced the complete genome of *S. mutans *serotype *c *strain NN2025, and compared it with the genome of UA159. The NN2025 genome is composed of 2,013,587 bp, and the two strains show highly conserved core-genome. However, comparison of the two *S. mutans *strains showed a large genomic inversion across the replication axis producing an X-shaped symmetrical DNA dot plot. This phenomenon was also observed between other streptococcal species, indicating that streptococcal genetic rearrangements across the replication axis play an important role in *Streptococcus *genetic shuffling. We further confirmed the genomic diversity among 95 clinical isolates using long-PCR analysis. Genomic diversity in *S. mutans *appears to occur frequently between insertion sequence (IS) elements and transposons, and these diversity regions consist of restriction/modification systems, antimicrobial peptide synthesis systems, and transporters. *S. mutans *may preferentially reject the phage infection by clustered regularly interspaced short palindromic repeats (CRISPRs). In particular, the CRISPR-2 region, which is highly divergent between strains, in NN2025 has long repeated spacer sequences corresponding to the streptococcal phage genome.

**Conclusion:**

These observations suggest that *S. mutans *strains evolve through chromosomal shuffling and that phage infection is not needed for gene acquisition. In contrast, *S. pyogenes *tolerates phage infection for acquisition of virulence determinants for niche adaptation.

## Background

The genomic heterogeneity within a bacterial species reflects its lifestyle, the niche it occupies, and its exposure to mobile elements, such as bacteriophages and plasmids [1]. Even though organisms belonging to the same genus/species have a common gene set (the core genome), individual organisms differ (strain-specific genes) in ways representative of the physiological and virulence properties of an organism [2,3]. Although not all genetic differences between strains are important for niche adaptation of the bacteria, strain-specific genes are thought to be responsible for the survival of an organism in its chosen niche. This variation can be due to genetic noise (i.e., indels, mobile- and selfish DNA) [4,5], gene loss [6,7], gene duplication [8] or modification of some of the existing genes [9,10]. Acquisition of new genes by lateral gene transfer is a predominant force in bacterial evolution. Laterally acquired genes provide a readily available novel pool of genes for developing physiological properties that are helpful for exploiting a new niche. A recent study suggested that the total known genome content (the pan-genome) of all contemporary *Streptococcus agalactiae *strains will increases as hundreds of genomes are sequenced [11]. Although *S. pyogenes *belongs to the same genus, it has a smaller pan-genome and greater level of recombination in its core genome [12]. These organisms provide a good model for identifying the causes of genome plasticity in human pathogens.

It has long been recognized that serological, genetic, and biochemical variations exist within the species *S. mutans *[13]. *S. mutans *has been classified into four serotypes (*c*, *e*, *f*, and *k*) based on the chemical composition of its cell surface rhamnose-glucose polymers [14]. We previously developed a multilocus sequence typing (MLST) method using eight house-keeping genes. Ninety-two sequence types (STs) were identified from 102 clinical isolates, indicating that *S. mutans *is a diverse population [15]. In the MLST analysis, serotype *c *strains were widely distributed in the dendrogram, while serotype *e, f*, and *k *strains were differentiated into clonal complexes. This suggests that serotype *c*, the dominant serotype among *S. mutans *clinical isolates (almost 80%), is the ancestral phenotype of this organism and that serotype *e *and *f *strains have evolved strain-specific genes. Although differences in modification of cell surface polymers reflect evolutionary trends, differences in cariogenicity have not been observed, and the relationship between serotype and clinical condition remains unclear.

Studies of individual *S. mutans *genes have revealed sequence variations, resulting in altered function of the encoded proteins [16-18]. For example, variation has been demonstrated in the occurrence of plasmids [19,20], and in mutacin operons [21], serotype antigens [22], competence [23,24], and the *msm, bgl, cel*, and *gftBC *loci [25-28]. Waterhouse and Russell recently showed a mosaic of loci such as the *msm, gbl, cel*, and *gftBC*, which they called "dispensable genes," distributed among *S. mutans *strains [27]. They also demonstrated that 20% of the *S. mutans *UA159 open reading frames (ORFs) were absent from one or more of the nine test strains, and dispensable ORF blocks (including more than one ORF) were identified by microarray analysis based on the UA159 genome [28]. Given the wide distribution and diversities of genotypes and genetic loci in *S. mutans*, it seems likely that other strains of *S. mutans *have both unique and common genetic loci not present on the UA159 genome [28,29]. This is useful for charting *S. mutans *evolutionary history. However, these analyses are based on only one genome, *S. mutans *UA159 even though extensive genomic variation between *S. mutans *strains has been predicted [30].

Genome sequence data are now available for numerous species of bacteria and comparative evolutionary approaches show positive selection pressure and lateral gene transfer in the evolution of many bacterial species. These analyses have been performed for pathogenic bacteria such as *Helicobacter pylori *[31,32], *Mycobacterium *species [33], *Chlamydia *species [34], *Escherichia coli *[35], and *Salmonella *species [36]. The pathogenic *Streptococcus *species include important human and agricultural pathogens [12,37]. More than 30 whole genomes of *Streptococcus *sp. belonging to nine different species including *S. pyogenes, S. pneumoniae, S. agalactiae, S. thermophilus, S. suis, S. sanguinis, S. gordonii, S. equi*, and *S. mutans *are publicly available. These organisms colonize diverse habitats including tooth, oral mucosal, pharyngeal, respiratory, intestinal, and urinogenital surfaces. These species have acquired various genes for a specific niche mainly by lateral gene transfer. For example, *S. pyogenes *acquires or tolerates bacteriophages that are important for new virulence determinants and that induce genomic rearrangement [38]. *S. agalactiae*, the main cause of neonatal infection in humans, also tolerates bacteriophages [11]. Some of these organisms gain counterattack systems such as restriction modification or clustered regularly interspaced short palindromic repeats (CRISPRs) [39,40]. Multiple sequences of genomes from closely related species that inhabit different niches lead not only to an understanding of the pattern of gene movement but also to insights into the role of species-specific genes, and genome plasticity.

In this context, we determined the whole genome sequence of an *S. mutans *serotype *c *strain NN2025 isolated from Japan in 2002, and we compared the genome sequence, genome structure, and gene variation with the genomes of serotype *c *UA159 strain isolated in 1982 from the United States, and with 95 clinical isolates from Japan and Finland, and other closely related streptococcal genera to provide useful information about the evolutionary events associated with *S. mutans *strains and *Streptococcus *sp., and to provide new insights into streptococcal species-specific survival strategies.

## Results and discussion

### General features of the strain NN2025

**Figure 1:**
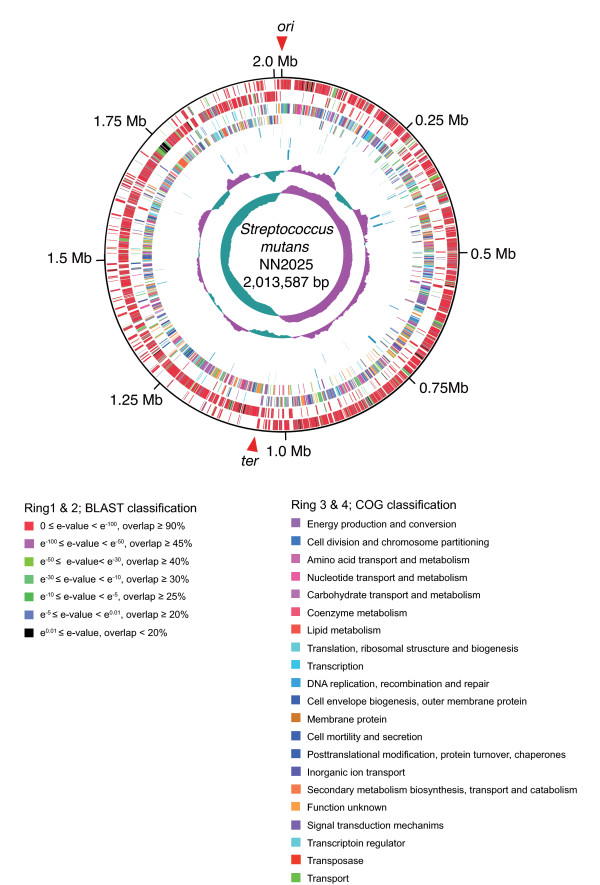
**Circular map of *S. mutans *strain NN2025**. The outer circle shows the scale (bp). From outside, rings 1 and 2 show the coding sequence (ORF) by strands (ring 1, clockwise; ring 2, counterclockwise). The predicted ORFs are distinguished by different colors in the BLAST analysis against the database (*see *Methods)(indicated as "BLAST classification"). Rings 3 and 4 show the ORF by different colors in the COG classification (indicated as "COG classification"). Ring 5 shows the location of transposase ORFs (including fragment), insertion sequence, and CRISPR associated ORFs. Rings 6 and 7 show the transfer RNA and ribosomal RNA genes identified in the genome. Rings 8 and 9 show the G + C content and GC skew, respectively. The red arrowheads indicate the origin of DNA replication (*ori*) and the putative region of replication terminus (*ter*).

The genome of NN2025 is a single, circular chromosome of 2,013,597 bp (Figure 1). The genome is almost identical in size to that of UA159 but is 17 kb shorter (additional file 1). Neither NN2025 nor UA159 contains plasmids or prophages. The genome start point for NN2025 was assigned to the putative location of the *dnaA *gene, similar to *S. mutans *UA159 and other genomic sequences [30,41]. The average GC content is 36.85%, which is similar to that of UA159. There are five rRNA operons containing 5S, 16S, and 23S rRNA genes. The 65 predicted tRNA genes encode all 20 amino acids. Most tRNA genes are clustered near the rRNA operons, 50 of 65 of these genes are less than 1 kb from an rRNA operon (Figure 1), as in other streptococcal strains [42,43]. The genome contains 1,895 predicted proteins with an average size of 903 bp that cover 85.18% of the whole sequence, which is similar to other streptococci. Both the sequence and the annotation have been deposited in the DNA Data Bank of Japan (DDBJ) (accession no. AP010655).

### Genes and regions conserved between *S. mutans *NN2025 and UA159 genomes

NN2025 contains 1,895 ORFs, of which 1,724 (90%) are predicted by reciprocal BLAST search analysis (BLAST E-value < 1 × 10^-5^) to be common to UA159. *S. mutans *strains NN2025 and UA159 are classified into the same serological group *c*, and show the same biochemical properties (e.g., fermentation of various sugars), adhesive properties to glass surfaces, and cariogenic properties in rat infection models (data not shown). Waterhouse et al. reported that 80% of the *S. mutans *ORFs are conserved among ten strains with different serotypes by microarray hybridization based on the UA159 genome, suggesting that 80% of ORFs are conserved within *S. mutans *strains [28]. This indicates that the core genome of *S. mutans *is more stable than that of other *Streptococcus *species, where the core genome represents only about 60% of the genome [12]. Most vegetative growth genes are highly conserved. Carbohydrate metabolism is a key survival strategy for *S. mutans *[30], and genes for transport and metabolism of various sugars, and the fermentation of carbohydrates are completely conserved between UA159 and NN2025. *S. mutans *is suggested to be capable of metabolizing a wider variety of carbohydrates than many other Gram-positive organisms that have been sequenced. *S. mutans *resides in the oral cavity, and this varied sugar metabolism is an important survival strategy for this organism. *S. mutans *is predicted to possess at least five sugar ABC transport systems and at least 14 sugar PTS systems, and can use at least 16 sugars for glycolysis. Nine PTSs were confirmed to be transcribed in the presence of 13 different sugars [44]. In addition, ORFs predicted as virulence factors of *S. mutans *including adhesins, glucan-producing and binding exoenzymes, were conserved. Adhesins of *S. mutans *are also important for protecting the bacterium against possible host defenses and for maintaining its ecological niche in the oral cavity. Comparative genome analysis revealed that the major *S. mutans *surface receptors SpaP (SMU.610 and SmuNN2025.1372) (also known as Pac, antigen I/II, etc.) and wall-associated antigen A (WapA) (SMU.987 and SmuNN2025.1036) are completely conserved. An uncharacterized wall-associated protein, WapE (SMU.1091 and SmuNN2025.0945), and a dextranase precursor (dexA; SMU2042 and SmuNN2025.1788) [45] were determined from BLAST analysis to be *S. mutans*-specific genes within the sequenced *Streptococcus *species (data not shown), suggesting that these might be important for colonization.

### Strain-specific genes and regions in NN2025 and UA159

Strain-specific regions were determined using MAUVE software, where the locally collinear blocks (LCBs) represent the landmarks (i.e. the homologous/conserved regions shared by all the input sequences) in chromosomes [46]. An LCB is defined as a collinear (consistent) set of multi-MUMs (exact match subsequences shared by all the considered chromosomes that appear once in each chromosome and are bordered on both sides by mismatched nucleotides). The weight (the sum of the lengths of the included multi-MUMs) of an LCB serves as a measure of confidence that it is a true homologous region instead of a random match. Therefore, the ORFs or sequences between the LCBs and any regions with low similarity (shown as white in LCB) are identified as strain specific regions.

**Figure 2:**
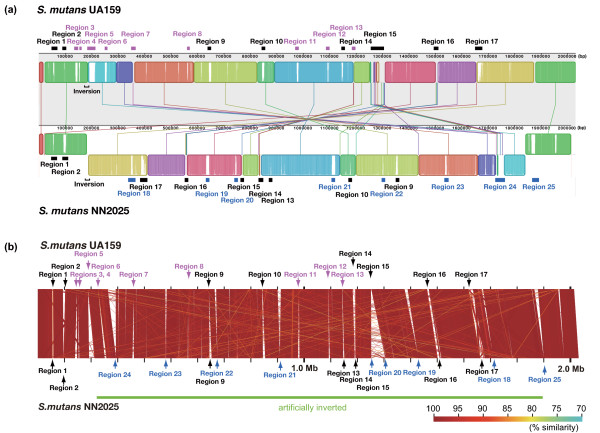
**Local collinear blocks (LCBs) between chromosomal sequences of the two strains of *Streptococcus mutans***. (a) Representation of the whole 32 local collinear blocks (LCBs) between chromosomal sequences of the two strains of *Streptococcus mutans*, UA159 and NN2025, was generated by MAUVE software at a minimum weight of 411. The *S. mutans *UA159 DNA sequence given on the forward strand is the reference against which the sequence of the NN2205 was aligned and compared. LCBs placed under the vertical bars represent the reverse complement of the reference DNA sequence. The 32 connecting lines between genomes identify the locations of each orthologous LCB in the two genomes. Unmatched regions within an LCB indicate the presence of strain-specific sequence. Each sequential block represents homologous backbone DNA sequence without rearrangements. Black horizontal bars indicate rearrangement regions and strain-specific regions analyzed in detail (*see *additional files 2 and 3). Blue region numbers and bars indicate NN2025-specific regions, pink region numbers and bars indicate UA159-specific regions, and black region numbers and bars indicate variable regions. Distributions of regions 1–25 are analyzed by long-PCR in 97 *S. mutans *strains including strains NN2025 and UA159 (*see *additional files 4 and 6). A dot plot to compare genome structure of these strains is also shown in Figure 6. (b) Alignment of the two genomes is generated by artificially correcting for the inversion of NN2025 at the *rrn-comX *region by the PROmer of MUMmer software and GenomeMatcher software (green bar). Similarity is shown by color code as represented in this figure. Blue region numbers and arrows indicate NN2025-specific regions, pink region numbers and arrows indicate UA159-specific regions, and black region numbers and arrows indicate variable regions.

The NN2025 genome contains eight strain-specific regions (Regions 18–25; Figure 2 and additional file 2) containing 65 ORFs that were unique in comparison with UA159. Other NN2025-specific ORFs are randomly inserted into the NN2025 genome, and these ORFs are classified into transposase fragments, ribosomal proteins, hypothetical proteins and disrupted gene fragments. Only two ORFs (SmuNN2025_1263, a putative transcriptional regulator, and SmuNN2025_1602, a putative RNA-directed DNA polymerase) are predicted to be functional genes from an unknown foreign source. In contrast, the UA159 genome contains nine strain-specific regions (Regions 3–8, and 11–13; additional file 3) containing 70 unique ORFs; another 88 UA159-specific ORFs are distributed in the UA159 genome (additional file 3).

Of the 25 regions that are unique between the NN2025 and UA159 genomes, eight regions (Regions 1, 2, 9, 10, 14–17) exist in both strains, but the contents of the ORFs are highly diverse. These regions are designated "variable regions". Region 1 contains genes of the purine nucleotide biosynthesis pathway (*pur *genes) and is thought to be important for bacterial growth, but genetic variation is found within this region. Region 2 in NN2025 includes two transposase fragments, five hypothetical proteins, methyltransferase (SmuNN2025_0077), *gapC *(SmuNN2025_0079), transcriptional regulator gene (SmuNN2025_0082), and *luxS *(SmuNN2025_0080). *luxS *plays an important role in the production of autoinducer-2 (AI-2) in many bacterial species, and 30% of the genes of *S. mutans *are affected by *luxS *[47]. Region 9 in NN2025 includes hypothetical proteins and ABC transporters; however, the contents of this region in UA159 are quite different and include hypothetical proteins that appear to have been horizontally transferred from other species. Region 10 in UA159 and Regions 14 and 17 in NN2025 include putative restriction/modification (R/M) system genes. The R/M system is composed of genes that encode a restriction enzyme and a modification methylase, and they defend against invaders by attacking non-self DNA, or by killing cells that have eliminated them [39].

Waterhouse et al. reported that 20% of UA159 ORFs did not hybridize with one or more of the nine *S. mutans *genomes in a UA159-based microarray analysis [28]. They also showed that 37 genomic blocks involving more than one ORF were found within the test strains. In the case of UA159, comparison of the regions identified in this study by PCR with previously reported 'genomic blocks' [28] and 'genomic island' [48] showed a good correlation, confirming the accuracy of both our results and the earlier reports, even though these were derived from different experimental approaches (additional file 3). The slight differences between the results, therefore, may have resulted from the characteristics of each method, because PCR primers target short sequences with high similarity, while microarray probes overall similarity to detect the target sequence. This second *S. mutans *genome sequence (NN2025) identifies new strain-specific content that could not be identified from only one genome. Based on their correspondence with the 'genomic islands', Region 1, 5, 8, 11, 12 and 15 appear to be horizontally transferred. The gene order of Region 15 in UA159 is different from that in NN2025, which may suggest the existence of an unknown mechanism to reorder the genomic island (additional file 3). These regions may reflect the phenotypic and biochemical differences of *S. mutans *strains. Although this comparative study was based on the UA159 genome, unique ORFs or regions in the genomes of other strains were not completely analyzed. Therefore, we also determined the differences of these regions among 97 test strains using long-PCR against 25 regions (additional files 4, 5 and 6) and clustering analysis (additional file 7). However, no characteristic differences in serotype specificity, geographical distribution, or the ability for sucrose-dependent adhesion to a glass surface were found (additional file 6). We have previously demonstrated that 102 clinical isolates from Japan and Finland were resolved into 92 STs by MLST [15], 85 of which were identified only once. We could not determine any distribution or lineage differences between Japanese isolates and Finnish isolates. We, therefore, conclude that the *S. mutans *population diversity is not caused by variation in gene content but probably with genetic recombination. In some *S. mutans *strains, similar insertion/deletion events appear in the genomes of strains with very different origins based on PCR determination of 14 loci [27]. In our analysis, 25 characteristic regions in the whole genome comparison were diverse, although they were isolated from only two geographical areas. This indicates that insertions/deletions in these specific regions are acquired by unknown mechanisms, probably by multiple acquisition events and the spread of an ancestral acquisition through the species by recombination from other bacterial strains. Various genes appear to be moved by lateral gene transfer in *S. mutans*, resulting in strain-specific regions. NN2025 possesses 16 transposase ORFs, and 16 IS elements in its genome, whereas UA159 possesses 34 transposon-like elements (additional file 1) [30]. These mobile genetic elements are located near the strain-specific regions. Thus, either laterally acquired genes account for the emergence of strain-specific ORFs or the comparator strain has lost these ORFs.

TnSmu1 was found to be a conjugative transfer element in UA159 [30]. This region in UA159 is large (~23 kb), involving SMU.191-226, one helicase gene (SMU.191c), two transposase-like genes (SMU.198c and 207c), and several hypothetical proteins. This region in NN2025 contains only seven short ORFs, including two transposase fragments (SmuNN2025_1728 and 1729). Among 97 test strains, 55 isolates, including all the Finnish isolates, show the same pattern as NN2025 (Region 5, Table 1), and 15.5% (15/97) of strains do not have this TnSmu1 region.

**Figure 3:**
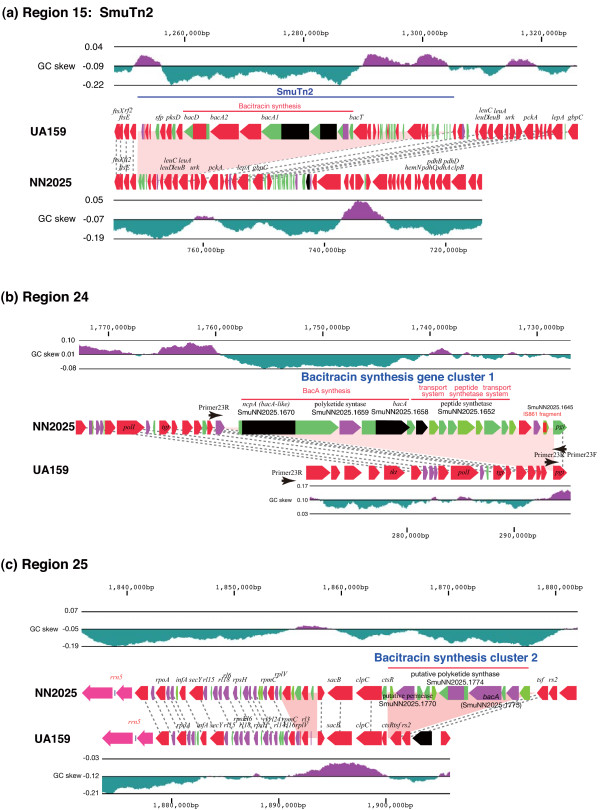
**Putative bacitracin synthesis clusters located in putative conjugative transposon in the different position of *S. mutans *UA159 and NN2025 genome**. Putative bacitracin synthesis clusters in *S. mutans *UA159 (a) and *S. mutans *NN2025 (b, c). The ORFs colors indicate the BLAST classification as shown in Figure 1. The BLASTP analysis was carried out across a non-redundant protein database in GenBank. The pink areas indicate the specific regions in each strain. Black dotted lines indicate orthologous genes that are located in identical relative positions or that are located in the inverted chromosomal regions. The whole gene list of these regions for each strain is shown in additional files 2 and 3.

Some isolates in Japan possess the same TnSmu2 as found in UA159 in a (~50 kb) genomic island, along with 29 ORFs predicted to be involved in biosynthesis and secretion of the antimicrobial peptide, bacitracin (Figure 3a). However, TnSmu2 has a high level of variation, including degenerated ORFs, and is not found in all *S. mutans *strains. In previous work, TnSmu2 was found only in UA159 and GS5 [27,28]. The variation in the TnSmu2 region has already been reported in strains Ingritt and LML7 [27], whose these sequences are almost identical to that of NN2025 (Figure 3a). Therefore, we constructed various PCR primer sets based on the sequence of UA159 and determined whether TnSmu2 was found in our test strains. In our experiments, 85% (75/88 Japanese isolates and 6/7 Finnish isolates) of clinical strains did not contain this region, and regional bias was not observed (Table 1). These observations indicate that the existence of TnSmu1 and TnSmu2 may not be common or necessary for the virulence of *S. mutans *strains in rats.

### Putative bacitracin synthesis regions

The bacitracin synthesis gene cluster in TnSmu2 did not exist in most of the clinical strains, and two other bacitracin or polypeptide antibiotic synthesis gene clusters were found in NN2025 (Figure 3). Bacitracin is a mixture of related cyclic polypeptide antibiotics produced by some strains of *Bacillus licheniformis *and *B. subtilis*. Its primary mode of action is to bind to undecaprenyl pyrophosphate (UPP) in the presence of a bound divalent metal cation, sequestering UPP and preventing its interaction with phosphatase [49]. This in turn prevents the return of the undecaprenyl monophosphate lipid carrier, inhibiting peptidoglycan and teichoic acid biosynthesis [50,51]. Region 24 is a large (~32 kb) transposon-like region containing bacitracin synthesis genes (SmuNN2025_1658, 1659, and 1660), peptide synthetase (SmuNN2025_1652), ABC transporters (SmuNN2025_1651 and 1655), and an *IS861 *fragment (SmuNN2025_1645) (Figure 3a). Smu2025_1660 and Smu2025_1658 are multiple-domain proteins similar to nonribosomal peptide synthetases such as gramicidin or bacitracin, but the sequence identity is lower (~24%) than that of the known peptide synthetases (Figure 3b). Region 25 (~13 kb) includes a nonribosomal peptide synthetase (SmuNN2025_1775), a putative polyketide synthase (SmuNN2025_1774), and a putative permease (SmuNN2025_1770); these genes are related to the synthesis of bacitracin or other peptide antibiotics (Figure 3c). *S. mutans *strains have bacitracin resistance [52]. The existence of bacitracin resistant genes (Smu.244 and SmuNN2025.1713) in this organism may reflect bacitracin production for protection. Regions 24 and 25 are distributed among 92.8% (85/92 Japanese isolates and 5/5 Finnish isolates) and 77.3% (71/93 Japanese isolates and 4/5 Finnish isolates), respectively (Table 1). Interestingly, all of the *S. mutans *strains used in this study possess at least one of these bacitracin or peptide antibiotic synthetases genes such as mutacins. In fact, Region 18 in NN2025 includes mutacin III (*mut III*) production genes (*scn *genes; SmuNN2025_0319, 0320, and 0326–0330; Table 1) [53]. SmuNN2025_0326 and _0327 are similar to the *scnM *and *scnT *of *S. pyogenes*, respectively, but these two genes were not found and an apparent fragmentation of the polyketide genes has been shown in UA159 [28,30]. Although bacitracin synthesis associated with TnSmu2 should be experimentally confirmed, NN2025 has a bactericidal effect (data not shown), indicating that the bacitracin production and *mutIII *cluster might be active in this strain, and that *S. mutans *has acquired peptide antibiotic synthetase genes to exclude other bacteria, perhaps for niche acquisition.

### CRISPR associated regions

Numerous prokaryote genomes contain structures known as clustered regularly interspaced short palindromic repeats (CRISPRs), composed of 25–50 bp repeats separated by unique sequence spacers of similar length [54,55]. It is well known that bacteria have a plethora of mechanisms to counterattack a diverse phage population [40]. Consequently, many bacterial species have developed a variety of natural defense mechanisms that target diverse steps of the phage life cycle, notably blocking adsorption, preventing DNA injection, restricting the incoming DNA, and abortive infection systems. In the genus *Streptococcus*, three distinct CRISPR loci have been identified within the *S. thermophilus *chromosome; namely, CRISPR-1, CRISPR-2 and CRISPR-3 [55,56]. CRISPR-1 and CRISPR-3 loci have been experimentally demonstrated to integrate novel spacers in response to bacteriophage [57,58]. Comparative analysis of CRISPR-1 sequences between various *S. thermophilus *strains has revealed polymorphisms [55]. In addition, it was recently reported that CRISPR provides acquired resistance against viruses in prokaryotes, notably in *S. thermophilus *[59].

**Figure 4:**
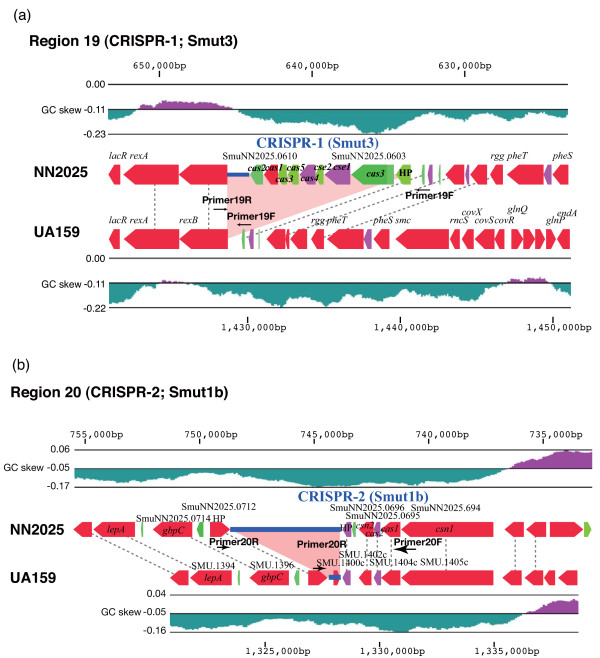
**CRISPR regions of *S. mutans *UA159 and NN2025**. CRISPR-1 region (Region 19) in NN2025 is a newly acquired region (a), and CRISPR-2 region (Region 20) is conserved between NN2025 and UA159 (b). The ORF colors indicate the BLAST classification as shown in Figure 1. The BLASTP analysis was carried out across a non-redundant protein database in the GenBank. HP; hypothetical protein. Blue lines indicate the palindromic repeat and spacer sequences. Pink areas indicate the specific regions in each strain. Black dotted lines indicate orthologous genes that are located in identical relative positions or that are located in the inverted chromosomal regions. The whole gene list of this region for each strain is shown in additional file 2 and additional file 3.

Phage tolerance capacity is known to be diverse [60]. For example, phage integration in *S. pyogenes *is an important source for new virulence factors as well as for the genomic rearrangement of the prophage regions [38,61]. Natural phage resistance mechanisms in *S. mutans *have not previously described, because phage donor and acceptor strains are quite rare in *S. mutans*. Based on in silico and in vivo analyses, several hypotheses have been put forward proposing roles for CRISPR and *cas *genes, including providing immunity against foreign genetic elements via a mechanism based on RNA interference [62]. Strain NN2025 possesses two characteristic CRISPR regions, regions 19 and 20 (Figure 4). Region 19 contains nine genes highly similar to the *cas *and *cse *genes (SmuNN2025_0603-0610) in *Lactobacillus casei *(BLAST E value < 10^-17^) (additional file 8). The gene order of this CRISPR region is similar to the "Ecoli" subtype [63], or to the Lcas2 of the Ldbu1 family recently named by Horvath [64], indicating that region is unique for *S. mutans*. It has been designated CRISPR-1 (or according to the nomenclature of Horvath et al., Smut3 [64]). The CRISPR-1 locus was present in 30/97 clinical isolates (Figure 4a; additional file 7). No variation of the length of repeat-spacer regions is found in these strains, indicating that this region might have been acquired in recent years or might be degenerate. The CRISPR repeat, direct repeat (DR), sequence length is 29 bp (ATT TTA CCC GCA CGA GCG GGG GTG ATC CT), and 18 spacer sequences are found adjacent to *cse*2 (SmuNN2025_0610) in NN2025. Of these 18 spacer sequences, six are identical to the sequence of the recently identified *S. mutans *phage M102 (additional file 9) [65].

**Figure 5:**
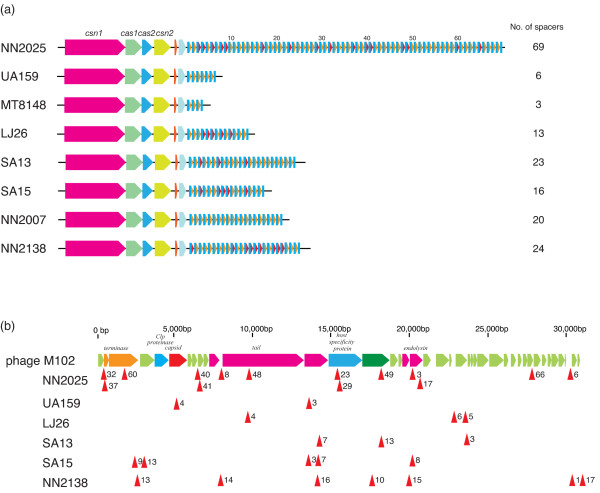
**Number of spacer sequences and its similarities against known phage genome of CRISPR in *S. mutans *strains**. (a) CRISPR-2 regions, widely distributed among *S. mutans*, in strain NN2025, UA159 and six selected strains (MT8148, LJ29, SA13, SA15, NN2207 and NN2138; *see *additional file 5 and 6). Blue rectangles indicate the direct repeat (DR). Orange triangles indicate spacer regions without homology to the phage M102 genome and red triangles are spacer regions corresponding to the sequence of the phage. Numbers of the spacer regions are determined by CRISPRfinder (*see *Methods for details). (b) Location of the spacer sequences corresponding to the M102 phage sequence (red arrowheads). The number of each arrowhead indicates the spacer number of each strain (additional file 9). The sequences of leader, spacer and repeat of CRISPR loci in the strain NN2025 and UA159 are listed in additional files 8, 9 and 10.

In contrast, another CRISPR region (Region 20) is found in both UA159 and NN2025, and has been designated as CRISPR-2 (or Smut1b [64]) in *S. mutans*. This CRISPR-2 locus consists of four *cas *and *csn *genes (SmuNN2025_0694-0696) and two hypothetical proteins (Figure 4b), and the gene order of the CRISPR-2 locus is almost completely conserved among *S. mutans *strains (92/97 strains; Table 1). Though the CRISPR subtype (Smut1 of Sthe3 family) in NN2025 is the same as in UA159, with the gene order, the lengths of the repeat sequences are quite different not only in these two strains but also among clinical isolates, ranging from 2 to 5 kb (data not shown). To determine the spacer variability in the clinical isolates, the CRISPR-2 loci of an additional six strains were sequenced for comparison (Figure 5). Strain NN2025 possesses a 36 bp DR sequence (GTT TTA GAG CTG TGT TGT TTC GAA TGG TTC CAA AAC), typical of the length of Sthe3 family, and 69 spacer sequences were found just downstream of *csn*2 [64]. This is the second example of a CRISPR locus with more than 50 repeats found in Firmicutes and it contains the highest number of repeats found in the genus *Streptococcus*. The five sequenced strains and UA159 all have the same DR sequence as NN2025, and only strain MT8148 has another 37 bp DR sequence (GTT TTG GAA CCA TTC GAA ACA ACA CAG CTC TAA AAC T) with the lowest repeat number in any tested strains. This is the first example of a repeat length other than from 36 bp in this Sthe3 family. However, the CRISPR-2 locus gene order was completely conserved among strains. The number of the spacers is quite divergent among strains, ranging from 3 to 69 (Figure 5a). We next determined the sequence similarity of spacer sequence with *S. mutans *phage M102 (Figure 5b). Of the 69 spacers of NN2025, 16 (23%) are highly similar to the sequence of *S. mutans *phage M102 (additional file 9) [65], as are 20–30% of spacers among the sequenced strains (additional file 10) with the exception of strain MT8148. Interestingly, strain LJ29 possesses two spacers similar to M102, and two spacers similar to *S. thermophilus *phages Sfi21 and 01205, indicating that *S. mutans *strains might have been attacked by these streptococcal phages, and protected by the CRISPR-2 locus.

The biological significance of CRISPR against phage infection has recently been reported in *S. thermophilus*. The presence of a CRISPR spacer identical to a phage sequence adjacent to *cas *provides resistance against phages containing that particular sequence. Limited information is available regarding the ecological role of bacteriophages in the oral cavity, although bacteriophages have been isolated from human saliva or dental plaque [66,67]. Only 16 lytic bacteriophages were isolated from more than 1000 plaque samples tested for lytic activity against indicator strains of *S. mutans *and *S. sobrinus *[65]. Therefore, the absence in both strain UA159 and NN2025 genomes of prophage or their remnants was not surprising. The long spacer sequences found in the CRISPR-1 and -2 loci in NN2025 (the longest in the genus *Streptococcus *with high variation in the CRISPR repeat number), and their sequence similarity to the lytic phage, suggest that *S. mutans *might resist phage infection via CRISPR. This observation is in contrast with the phage-permissive *S. pyogenes*, because *S. pyogenes *possesses two to eight prophage regions within its genome (average 4.5 per genome), and the number of CRISPR-associated direct repeats and spacers is less than that of other streptococcal species (additional file 11). The average number of prophages in *S. pyogenes *that lack the *cas *genes found in *S. mutans *is six (5–8 prophages; MGAS315, SSI-1, Manfredo, MGAS10394, MGAS8232), while that in the other eight *S. pyogenes *strains carry the same *cas *gene set is 3.5 (2–5 prophages; data not shown). The number of prophages in an *S. pyogenes *genome may be affected by the CRISPR gene set present, and further examination is needed, especially considering that the many sequenced genomes of streptococci carrying prophages have rarely demonstrated natural transformation [68]. Therefore, in the genus *Streptococcus*, specific species may have evolved to function in a particular way to acquire foreign genes via natural transformation or bacteriophages, and the acquisition of new foreign genes via phage infection may not be favorable for the lifestyles of *S. mutans *in the oral cavity. Elucidation of the mechanism by which *S. mutans *acquires new genes will be interesting to clarify further the species-specific evolutionary strategies in the streptococci.

### Genomic analysis of the rearrangement in the UA159 and NN2025 genomes

The physical structure of the bacterial genome is highly conserved during evolution in *Escherichia coli *[69,70]. However, X-shaped chromosomal inversion has been found between *Pyrococcus horikoshii *and *P. abyssi *[71], and between *Chlamydia pneumoniae *and *C. trachomatis *[34]. Interestingly, a typical X-shaped chromosomal inversion is found in *S. pyogenes *[38,72]. Such rearrangements leading to genome plasticity in these bacteria might reflect or contribute to evolutionary processes in the creation of new pathogens.

The recombination sites near the *ori *region in NN2025 are found within the 16S rRNA, tRNA-Ala, 23S rRNA, tRNA regions, and the *comX1 *homologue in both regions (data not shown). The genome of *S. mutans *NN2025 shows that the rearrangement breakpoint is located 86 bp downstream from the 23S rRNA, and this rearrangement breakpoint is conserved within the other *S. mutans *strains isolated in Taiwan in which Huang et al. firstly observed chromosomal inversion in 25/58 strains [73]. These observations indicate that the chromosomal recombination may frequently occur in this genomic site because isolates from Japan, Finland and Taiwan shows the same inversion site though the frequency differs between our strains (83/97) and those reported by Huang et al. (25/58), and might occur by *recA*-dependent recombination between two ribosomal operons [74]. In Gram-negative *Salmonella typhimurium*, inversion between large inverted repeats (>5 kb) separated by large intervals (>60 kb) has been shown to be *recA*- and *recB-*dependent [75]. In addition, chromosomal inversion between the ribosomal operons was reported between *S. typhimurium *and *S. paratyphi *A [76]. Our sequencing analysis showed that long repeated sequences across the replication axis can also induce large-scale chromosomal rearrangements within a species of Gram-positive bacteria.

Although the role of homologous recombination in genomic rearrangements across the replication axis has not been elucidated, Tillier and Collins proposed an alternative model for the observed pattern of rearrangement [74]. Gene translocation across the replication axis may result in close physical proximity during the process of genome replication because homologous recombination equidistant from the *ori *region occurs between two replication forks and single- or double-stranded DNA breaks [77]. In fact, two *rrn-comX *regions were found equidistant from the *ori *region in the *S. mutans *genomes; thus, this model is in good agreement with the genomic rearrangement mechanism of *S. mutans *and *S. pyogenes*. This sequence specificity may affect site-specific recombination during homologous recombination.

**Figure 6:**
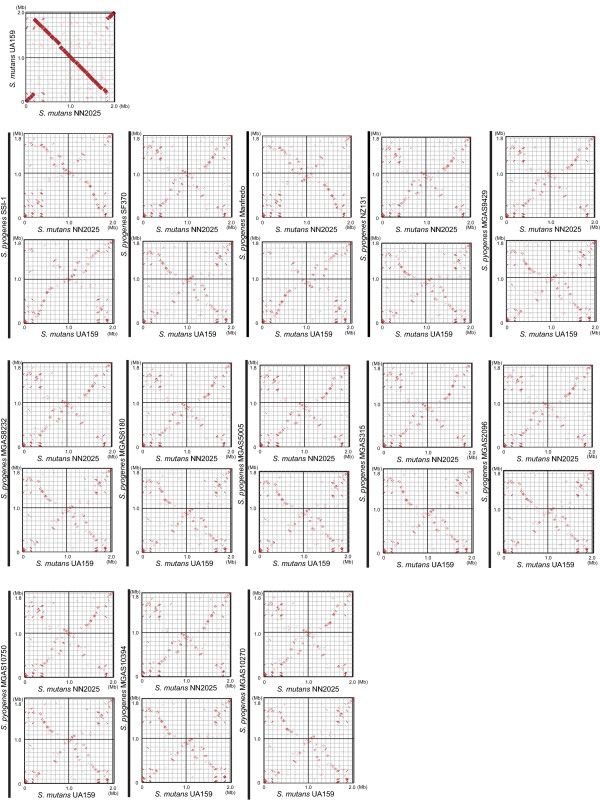
**Genome comparison of two *S. mutans *strains, or each *S. mutans *strain against 13 *S. pyogenes *strains based on the chromosomal organization of strain NN2025 or UA159**. Dot plots of *S. mutans *NN2025 vs 13 *S. pyogenes *strains and of *S. mutans *UA159 vs the same set of *S. pyogenes *strains are presented. These were generated by PROmer of MUMmer software and were visualized with the GenomeMatcher software (*see *Methods).

Alignments and dot plots of the genomes of *S. mutans *NN2025 and UA159 revealed extensive genomic rearrangements (Figures 2 and 6). The origin of DNA replication (*ori*) [30,78] and *dif*-like termination sequence (*ter*) [30] were conserved. Both alignment and dot plot analyses using MAUVE and MUMmer software revealed X-shaped chromosomal inversions that were symmetrical across the replication axis [46]. The locations of the genes around the *ori *region (from 1,850 kb to 190 kb) were almost completely conserved between NN2025 and UA159, and the other homologous genes were translocated to an inverted position on the chromosome. These chromosomal segments are symmetrical except for small gaps encoding the genes of strain-specific regions (Figure 2). As a consequence, the inversion in NN2025 relative to UA159 did not change gene orientation relative to the replication axis and produced an X-shaped DNA dot plot [74,79].

For *S. pyogenes *Manfredo and *S. pyogenes *SSI-1, there is a large central inversion (~1.3 Mb), which probably resulted from reciprocal recombination between *rrn-comX *regions that are a similar distance from the terminus of replication [72], and there is an additional (~200 kb) inversion near the terminus of SSI-1 caused by reciprocal recombination between prophages across the replication axis, possibly generating novel recombinant prophages with alternative cargos [38]. The comparison between *S. mutans *strains, therefore, could provide evidence of both intraspecies and interspecies genomic rearrangement in streptococci.

### Comparison of genome content and architecture of *S. mutans *species with other streptococcal species

Extensive comparative genomic analyses of positive selection, recombination, and the genome composition of 26 *Streptococcus *genomes from six different species were reported by Lefébure and Stanhope [12]. In this study, the analyses concentrated on the comparison of *S. mutans *species-specific gene groups and, because limited information is available regarding overall symmetry between the genomes of various streptococcus species, on the genome-wide rearrangement within nine species (32 genomes). To identify sets of gene groups shared between two *S. mutans *strains or unique to each species/strain, the predicted ORFs from the 32 streptococcal strains were clustered into groups using reciprocal BLASTP analysis. The set of shared gene groups (core genome) for each species was determined to compare the numbers of species/strain-specific gene groups and those shared with *S. mutans *(*see *Methods, additional file 12). A total of 4740 and 573 gene groups, respectively, comprised the pan- and core-genomes of the genus *Streptococcus*, based on 32 complete genomes (data not shown). Eighty-one percent of gene groups were shared between the two *S. mutans *strains. Comparison of the 1069 groups constituting the core genome of *S. mutans *with other bacterial species/strains showed that 49.9% to 66.3% of gene groups are conserved between *S. mutans *and other streptococcal genomes (additional file 12). From the 16S rRNA sequences, these species can be grouped into the pyogenic group (49.9–56.2% of gene groups shared with *S. mutans*), the mutans group (*S. mutans*), the mitis group (57.4–68.3% of gene groups shared with *S. mutans*), the salivarius group (61.2% of gene groups shared with *S. mutans*), and others (51.7% of gene groups shared with *S. mutans*) [80]. This leads to difficulties in distinguishing between the species based on the number of shared gene groups. These difficulties result only from the analysis of complete extant genomes, and the availability of fewer complete genomes in a species significantly affects the number of species-specific genes identified. Sequencing of further genomes in each species will be useful for defining actual species-specific genes.

As hypothesized by Lefébure and Stanhope [12], *S. agalactiae *has a larger pan-genome and less recombination than *S. pyogenes *as evidenced by the species having a larger habitat and gene pool diversity. This is supported by the fact that the pan-genomes of ecologically restricted organisms are smaller than those of bacteria that adopt a more generalist lifestyle [81]. Although *S. mutans *may have a smaller pan-genome and more recombination than *S. agalactiae *because it is usually isolated only from dental caries, the analyses here concentrated on the *S. mutans *species-specific regions as discussed, comparing NN2025 and UA159 to understand the species (116 gene groups are shared only within *S. mutans*, and nine gene groups are shared without *S. mutans*). In addition, long-PCR analysis of the dissimilar regions in the genome reveals the genomic diversity of the species, as well as the results of MLST analysis previously reported by Nakano et al[15,82] (additional file 6).

*S. mutans*, *S. sanguinis *and *S. gordonii *are classified as oral streptococci. Although *S. mutans *and the viridans streptococci (*S. sanguinis *and *S. gordonii*) are further classified into another subgroup within the non-pyogenic streptococci [83], they have evolved to adapt to the oral environment. Therefore, we compared the gene groups common to oral streptococci with the other streptococcal species to identify the features of oral streptococci. In total, 31 gene groups of *S. mutans *NN2025 were identified as common to oral streptococci but not to other streptococci (additional file 13). Among them, genes for histidine metabolism are conserved among oral streptococci (NN2025.0784, 0785, 0787, 0790–0793). In particular, histidine biosynthesis genes related to the conversion of 5-phosphoribosyl-α-1-pyrophosphate to L-histidine are completely conserved between oral streptococci. The physiological importance of histidine biosynthesis for colonization in the oral cavity is unclear, but we speculate that the presence of histidine biogenesis in situ may be more beneficial in oral environment than importing it from outside.

To confirm the chromosomal inversion at the gene level, we further compared the order of orthologous genes in *S. mutans *and *S. pyogenes *species (additional file 14) and other streptococcus strains (data not shown) [11,42,84,85] using MAUVE and MUMmer software. As shown in Figure 6, an X-shaped symmetrical DNA dot plot was observed when comparing *S. mutans *NN2025 and *S. pyogenes *strains. Interestingly, an X-shaped symmetrical DNA dot plot is also found for the comparison between the genomes of *S. mutans *UA159 and *S. pyogenes *strains, as well as for the comparison of the two *S. mutans *strains (Figure 2). This X-shaped symmetrical DNA dot plot was also found when comparing the two *S. mutans *strains with other streptococcal strains of different species (additional files 15, 16, 17, 18). These observations indicate that chromosomal inversion across the replication axis occurs frequently in a number of streptococcal species.

We also determined the sequence alignment for the ORF order between *S. mutans *and other streptococcal strains using MAUVE software. The genome structure of *S. pyogenes *is more highly conserved against *S. mutans *strains than against other streptococcal strains (data not shown). The *ori *and *ter *regions of these two species are highly conserved, and there are fewer short LCBs (0–25 kb) than in other streptococcal strains. This suggests that there are fewer genome rearrangements between *S. mutans *and *S. pyogenes *than between other streptococcal species. We hypothesize that these two species occupy similar ecological niches or smaller habitats and gene pools than *S. agalactiae *(additional file 19; [2]). The eight strains of *Yersinia pestis *and *Y. pseudotuberculosis *include 78 LCBs, although these strains are included within the same species [86]. Interestingly, the LCBs in *S. mutans *and *S. pyogenes*, except for strain-specific mobile genetic elements, seem to shuffle across the replication axis. Chromosomal inversions were also observed between several *S. pyogenes *strains (additional file 14), indicating that chromosomal shuffling of an array of ORFs as a unit has occurred following branching from a common ancestor. However, it is not clear why chromosomal rearrangements are so frequently found within the genus *Streptococcus*, although the abundance of mobile elements in the genome might affect the rearrangement distance [87].

In circular bacterial genomes, DNA replication divides the genome into two domains called replichores. In the case of *S. pyogenes*, an undesirable phage integration (more than 100 kb) into one replichore may cause an unstable genome structure, and this unbalanced genome structure might induce chromosomal inversion between highly conserved regions [38]. Furthermore, a survey of *Salmonella *genomes in culture has provided evidence that genomes with equally sized replichores (balanced replichores) may be under positive selective pressure [88]. There is also evidence that chromosome organization might influence rates of rearrangement [89], and that a genome structure unbalanced by artificial genomic inversion induces an instability, that is detrimental to cell fitness or led to cell death in *Lactococcus lactis *[90]. These observations suggest that rewinding an unstable genome (i.e., making an unbalanced genome structure wind off to produce equally sized replichores) may be important for survival of this organism. Two large conjugative transposon-like regions, TnSmu1 and TnSmu2, have been inserted in UA159, with TnSmu1 located near the *rrn2 *region of UA159. These regions are not found in NN2025. In addition, the similarity in genome structure between *S. mutans *and *S. pyogenes *suggests two possibilities regarding the chromosomal inversion in *S. mutans*. One option is that chromosomal rearrangement of *S. mutans *NN2025 and UA159 happened by chance. No characteristic differences of serotype specificity, geographical distribution, STs, or topological pattern from long-PCR analysis (additional file 6) was observed in these strain-specific regions, indicating that these regions might be acquired by chance as suggested by Waterhouse and Russell [28]. In *L. casei*, topological discrepancies between the MLST and pulse-field gel electrophoresis typing trees were observed, suggesting that intragenic point mutations have accumulated at a slower rate than indels and genome rearrangements [91]. Therefore, the species-specific position and rearrangement of these genes could have occurred faster than the evolution of each protein, resulting in interference from the ancestor in the long-PCR analysis. The other option is that this inversion occurred in UA159 by integration of foreign DNA into one replichore. However, the fact that the frequency of clinical isolates with chromosomal inversion was 83 of 97 test strains by long-PCR analysis (Table 1) may indicate that the chromosomal orientation found in NN2025 is the basic genome architecture of *S. mutans *strains. Further examination should be carried out with strains isolated from regions in addition to Japan and Finland. Following the discussions in Hendrickson and Lawrence [92] and Song et al. [93], we hypothesize that the asymmetry observed in *S. mutans *and *S. pyogenes *is under selection and will probably balance through amelioration [94], which has not been experimentally or theoretically demonstrated in the genus *Streptococcus*.

## Conclusion

Analysis of the *S. mutans *genome and its comparison with other streptococcal species revealed new insights into species-specific survival strategies. The chromosomal inversion across the replication axis between *S. mutans *strains occurred between homologous ribosomal operons located almost the same distance from the *ori *region as the *S. pyogenes *chromosomal inversion. This chromosomal inversion, a characteristic X-shaped symmetrical DNA dot plot, is found not only between *S. mutans *and *S. pyogenes *strains but also between *S. mutans *strains and all the streptococcal species in which the complete genome sequence is known. These observations indicate that the chromosomal inversion across the replication axis has occurred frequently within streptococci during evolution. This would possibly maintain the distortion of the replichore induced by the insertion of foreign genetic elements such as phage integration and/or generate genetic shuffling to create a novel genetic pool.

The *S. mutans *genome may preferentially defend against phage integration using CRISPR and/or various restriction/modification systems. These counterattack systems are heterogeneous in the genus *Streptococcus *as some streptococcal species tolerate phage integration. In the case of *S. pyogenes *and *S. agalactiae*, 10–20% of ORFs consist of phage genomes, and counterattack systems for phage integration exist but seem not to work efficiently. In contrast, *S. mutans *might have evolved to avoid acquiring genes via phage-mediated transduction in order to protect their genome from the physical distortion via phage integration. Alternatively, it may permit the integration of CRISPR via conjugative plasmid [95,96] and of antimicrobial peptide synthesis systems (bacitracin synthetase clusters) via conjugative transposons and/or may have evolved to use natural transformation as the preferred method system to acquire new genetic traits. This notable feature of foreign gene acquisition in *S. mutans *suggests that the genomic variation of the species might contribute to maintaining its niche. There remain more questions regarding how the oral environment affects gene acquisition in *S. mutans*. We believe that these findings will lead to new insights into the mechanisms of evolution in other streptococcal species.

## Methods

### Bacterial strains

*S. mutans *strain NN2025 was isolated in Japan in 2002 from a patient with dental caries, obtained from the Pedodontics Clinic of Osaka University Dental Hospital, Suita, Osaka, Japan, with informed consent according to the protocol approved by the Ethics Committee of Osaka University. It produces glycosyltransferases, and its serotype was determined to be *c *by immunodiffusion test using serotype-specific antibodies. As a control, *S. mutans *UA159 (ATCC No. 700610, UAB577) was purchased from the American Type Culture Collection. Other clinical isolates and laboratory strains have been isolated at Osaka University and Helsinki University. These strains were selected from our culture collection and all of the strains were confirmed to be *S. mutans *based on conventional physiological tests, including rough colony morphology on mitis-salivarius agar (Difco Laboratories, BD Diagnostics, Sparks, MD, USA), bacitracin resistance, and fermentation of sorbitol, mannitol, raffinose, or melibiose (1% each) in a phenol red broth base (Difco). Bacterial genomic DNA was extracted using DNAeasy kit according to the manufacturer's instruction (Qiagen, Valencia, CA, USA).

### Genome sequencing and annotation

The initial stage of sequencing was performed using whole genome random shotgun methods with sheared chromosomal DNA from strain NN2025. We constructed a pUC18-based library containing 1–2 kb and 4–5 kb inserts, and sequenced 48,000 clones (12.6-fold coverage) with Big-Dye terminator chemistry and ABI 3730 × l sequencer (Applied Biosystems, Foster City, CA, USA) and with ET-Dye terminator chemistry and MegaBACE 4500 sequencer (GE Healthcare, Uppsala, Sweden). The sequence was assembled using Phred/Phrap/Consed [97-99]. Gaps in the sequence were filled by direct PCR sequencing, using primers constructed to anneal to each end of neighboring contigs. Finally, the entire sequence was estimated to have an error rate of less than 1 per 10,000 bases (Phrap score >= 40). To verify and determine the assembled sequences, a total of 90 primer sets were constructed to cover whole chromosomal DNA of strain NN2025 at the unique flanking sequence, and 18–25 kb of long-PCR was performed by the LA-PCR method (Takara, Otsu, Japan). Large repeated elements in the genome (700–6000 bp) such as the 16S and 23s rRNA operons (*rrn*) were amplified from chromosomal DNA using LA-Taq (Takara), sequenced, and assembled independently, as described above.

ORFs >60 bp were identified and annotated separately using Metagenome gambler light (MGGL, ver. 2.1.5), in silico Molecular Cloning Genomics Edition (IMC-GE, ver. 3.0.30) (Insilico biology, Yokohama, Japan) [100] and GLIMMER 2 [101,102]. The predicted ORFs were reviewed individually by a manual search for start codons on the basis of ribosomal-binding motifs. ORFs were further compared across a non-redundant protein database in the GenBank using BLASTP software (version 2.2.3) [103]. Functional motifs and the domains of proteins were identified by searches against Prosite, Blocks, and Pfam database [104] and phi-BLAST [105]. Protein localization and transmembrane domains were predicted by combining PSORT with the rule set for gram-positive bacteria [106,107], and the SOSUI/SOSUI signal program [108,109]. Cell-wall attachment motifs (LPXTG) and secreted protein motifs (sortase recognition motif) were identified with IMC-GE. The predicted ORFs are distinguished by different colors (red; E-value is between 0.0 to 1.0 × 10E-100, and overlap is over 90%, pink; E-value is between 1.0 × 10E-100 to 1.0 × 10E-50, and overlap is over 45%, light green; E-value is between 1.0 × 10E-50 to 1.0 × 10E-30, and overlap is over 40%, green; E-value is between 1.0 × 10E-30 to 1.0 × 10E-10, and overlap is over 30%, dark green; E-value is between 1.0 × 10E-10 to 1.0 × 10E-5, and overlaps is over 25%, light blue; E-value is between 1.0 × 10E-5 to 0.01, and overlap is over 20%, and black indicates no homologous genes by BLAST analysis against the database (*see *BLAST classification of Figure 1 for the actual colors). Functional categories based on the analysis of clusters of orthologous genes were assigned by using COGnitor [110,111]. Transfer RNA genes were identified using tRNAscan-SE [112].

### Comparative genomes of *S. mutans *strain and other Streptococcal species

The thirty-one genomic sequences of *S. mutans *UA159 (AE014133), *S. pyogenes *strains (SSI-1: BA000034, SF370: AE004092, Manfredo: AM295007, MGAS315: AE014074, MGAS5005: CP000017, MGAS6180: CP000056, MGAS8232: AE009949, MGAS9429: CP000259, MGAS5005: CP000017, MGAS10270: CP000260, MGAS10394: CP000003, MGAS10750: CP000262, and NZ131: CP000829), *S. pneumoniae *strains (D39: CP000410, R6: AE007317, TIGR4: AE005672, CGSP14: CP001033, G54: CP001015, and Hungary 19A-6: CP000936), *S. agalactiae *strains (2603V/R; AE009948, A909: CP000114, and NEM316: AL732656), *S. sanguinis *SK36 (CP000387), *S. suis *strains (05ZYH33; CP000407, and 98HAH33; CP000408), *S. thermophilus *strains (LMD-9; CP000419, CNRZ1066; CP00024, and LMG18311; CP000023), *S. gordonii *Challis CH1 (CP000725), and *S. equi zooepidemicus *MGCS10565 (CP001129) were obtained through the website of the National Center of Biological Information (NCBI) [113]. Alignment of the complete genomic sequences of these bacterial strains was accomplished with MAUVE Genome alignment software [46,114], or with the PROmer of MUMmer software [115], following visualization with the GenomeMatcher software [116]. SPRING software was used for comparison of LCB lengths [117,118] All the predicted ORFs from the 32 streptococcal strains were clustered into groups based on the threshold of maximum E-value = 10^-5 ^in the reciprocal BLATP analysis and the extant common cluster set (core genome) of each species was determined to compare the numbers of species-specific gene groups and those shared with *S. mutans*.

Analysis of CRISPR loci was carried out based on the method of Horvath et al. [64] with slight modifications as follows. For published genomes, CRISPR loci were retrieved from the database of CRISPRdb [119]. For *S. mutans *NN2025 genome, the detection of CRISPR loci was carried out using CRISPRFinder web service [120,121] or the Repeat Search and Dot Plot of IMC-GE. Non-coding sequences located at the 5' end of the first identified CRISPR repeat for each locus were selected as putative leader sequences and compared using the Dot Plot of IMC-GE. BLASTN was used for similarity searches between CRISPR sequences and existing sequences in the DDBJ [122] database limited to viruses (ddbjvrl and ddbjphg) or bacteria (ddbjbct). Similarly, matches showing an expected value below 0.01 and/or streptococcal phages found in viruses entries were retained, and the spacers with no similarity were further subject to BLAST analysis with bacterial entries and the matches were retained; the matches to sequences found within CRISPR loci were ignored.

Strain-specific regions and rearrangement sites depicted in Figure 2 in *S. mutans *strains were examined by the LA-PCR method (Takara, Otsu, Japan) using site-specific primer pairs (additional file 4). Briefly, PCR was performed as follows: 95°C for 1 min for one cycle, 98°C for 10 sec and 68°C for 3–20 min (according to the length of the amplicon) for 30 cycles, and 72°C for 10 min for one cycle. The amplified fragments were separated on 0.8% agarose gel electrophoresis and visualized by ethidium bromide staining.

## List of abbreviations

PFGE: pulsed-field gel electrophoresis; MLST: multilocus sequence typing; STs: sequence types; LCBs: locally collinear blocks; *ori*: origin of DNA replication; *ter*: *dif*-like termination sequence; ORF: open reading frame; IS: insertion sequence; SNP: single nucleotide polymorphism; R/M system: restriction/modification system; CRISPRs: clustered regularly interspaced short palindromic repeats; DR: direct repeat; UPP: undecaprenol pyrophosphate.

## Authors' contributions

FM and AS conceived of the studies, designed them, performed the computational analysis, and drafted the manuscript. KK and KN participated in the design of the study and bioinformatics analysis. KN and RN carried out the isolation of clinical strains. MK and KN participated in bioinformatics analysis. SK, TO and SH conceived of the studies and participated in manuscript drafting. MH generated the genomic sequence. IN conceived of the studies, participated in their design and coordination, and drafted the manuscript. All authors read and approved the final manuscript.

## Supplementary Material

Additional file 1 General features of *S. mutans *strains NN2025 and UA159. Click here for file

Additional file 2 ***S. mutans *NN2025 specific ORFs**. Different regions within the PCR region are shown in different colours and correspond to the regions shown in Figure 2. No coloring in the PCR region indicates the absence of the ORF in the regions in Figure 2. Click here for file

Additional file 3 ***S. mutans *UA159-specific ORFs**. Different regions within PCR region are shown in different colors and correspond to the regions shown in Figure 2. No coloring in the PCR resion indicates the absence of the ORF in the regions in Figure 2. Click here for file

Additional file 4 Long-PCR analyses of genomic rearrangement region and insertion/deletion regions of *S. mutans *strains. Click here for file

Additional file 5 **Primers used for detection of rearrangement and strain-specific regions in *S. mutans *strains**. Each strain-specific region is visualized as a black bar in Figure 2. Click here for file

Additional file 6 Characteristics of *S. mutans *reference strains and clinical isolates used in this study. Click here for file

Additional file 7 **Cluster analysis based on strain-specific regions showing the relationship between *S. mutans *strain NN2025, UA159 and 95 clinical isolates**. Long-PCR results were converted to numerical values according to the length of the PCR products, then complete linkage clustering was performed on CLUSTER software and visualized with Java Tree view software (contrast value; 0 to 3.0). Click here for file

Additional file 8 **Characteristics of CRISPR loci found in *S. mutans *NN2025**. The nomenclature, leader sequence, repeat sequence, number of repeats, and similarity were determined based on the method of Horvath et al. [64] with slight modifications (*see *Methods for details). Click here for file

Additional file 9 **Sequence similarities of the existing CRISPR spacers in *S. mutans *NN2025**. The spacer similarities were determined by BLASTN against viruses including bacteriophage or bacteria databases (*see *Methods). No description in "origin" or "BLAST E-value" indicates that no similarity was found in the database. Click here for file

Additional file 10 **Distribution of CRISPR-2 (Smut2b; Sthe3 family)-associated repeat sequences in genus *Streptococcus***. Repeat sequences in the CRISPR-2 homologous region (Sthe3 family) were found in 19 of 32 streptococcal genomes (*see *Methods; for NN2025, *see *additional file 8). Similarities of the direct repeat sequence of the strain NN2025 (Sthe3 family) were examined by BLASTN against each genome as a target database. The number of repeats was determined based on Horvath et al. [64] (*see *Methods). Click here for file

Additional file 11 **Distribution of CRISPR-2 (Smut2b)-associated repeat sequences in genus *Streptococcus***. Repeat sequences in the CRISPR-2 homologous region (Smut2b; Sthe3 family) found in streptococcal genomes except *S. mutans *NN2025 (15/31 strains; *see *Methods). Similarities were examined by BLASTN against each genome as a target database. The number of repeats was determined as described by Horvath et al. [64] (*see *Methods). Click here for file

Additional file 12 **Venn diagrams for the clustered gene groups in *S. mutans *species and eight other streptococcal species**. All the predicted ORFs from the 32 streptococcal strains were clustered into groups based on a threshold of maximum E-value = 10^-5 ^in the reciprocal BLATP analysis to compare the numbers of species-specific and -shared gene groups and those shared with *S. mutans *species. The number in the figure was the number of the gene groups, not of the ORFs (see Methods for details). Click here for file

Additional file 13 **Specific gene groups shared in oral streptococci**. All the predicted ORFs from the 32 streptococcal strains were clustered into groups based on a threshold of maximum E-value = 10^-5 ^in the reciprocal BLATP analysis to extract the ORF(s) of gene groups specific for oral streptococci (*S. mutans, S. sanguinis*, and *S. gordonii*). The predicted function is assigned based on the COG classification (see Methods for details). Click here for file

Additional file 14 **Comparison of genomic shuffling between two strains of *S. mutans *(N2025 and UA159) and three strains of *S. pyogenes *(SSI-1, SF370 and Manfredo)**. A MAUVE representation of the total 64 local collinear blocks (LCBs) between chromosomal sequences of the *S. mutans *strains and *S. pyogenes *strains, at a minimum weight of 144. The *S. mutans *NN2025 DNA sequence given on the forward strand is the reference against which the sequence of the NN2205 was aligned and compared. LCBs placed under the vertical bars represent the reverse complement of the reference DNA sequence. LCBs placed under the vertical bars represent the reverse complement of the reference DNA sequence. The 64 connecting lines between genomes identify the locations of each orthologous LCB in the two genomes. Unmatched regions within an LCB indicate the presence of strain-specific sequence. Each sequential block represents a homologous backbone DNA sequence without rearrangements. Click here for file

Additional file 15 **Genome comparison of each *S. mutans *with six *S. pneumoniae *strains based on the chromosomal organization of the strain NN2025 or UA159**. Dot plots of *S. mutans *NN2025 vs. six *S. pneumoniae *strains and of *S. mutans *UA159 vs the same set of *S. pneumoniae *strains are presented, as generated by PROmer of MUMmer software and visualized with GenomeMatcher software [116](*see *Methods). Click here for file

Additional file 16 **Genome comparison of each *S. mutans *with three *S. agalactiae *strains based on the chromosomal organization of the strain NN2025 or UA159**. Dot plots of *S. mutans *NN2025 vs. three *S. agalactiae *strains and of *S. mutans *UA159 vs. the same set of *S. agalactiae *strains are presented, as generated by PROmer of MUMmer software and were visualized with the GenomeMatcher software [116] (*see *Methods). Click here for file

Additional file 17 **Genome comparison of each *S. mutans *with three *S. thermophilus *strains based on the chromosomal organization of the strain NN2025 or UA159**. Dot plots of *S. mutans *NN2025 vs three *S. thermophilus *strains and of *S. mutans *UA159 vs. the same set of *S. thermophilus *strains are presented, as generated by PROmer of MUMmer software and visualized with the GenomeMatcher software (*see *Methods). Click here for file

Additional file 18 **Genome comparison of each *S. mutans *with two *S. agalactiae *strains, *S. sanguinis *SK36, *S. gordonii *Challis CH11, *S. equi zooepidemicus *MGCS10565 based on the chromosomal organization of the strain NN2025 or UA159**. Dot plots of *S. mutans *NN2025 vs. two *S. agalactiae *strains, *S. sanguinis *SK36, *S. gordonii *Challis CH11, and *S. equi zooepidemicus *MGCS10565 and of *S. mutans *UA159 vs. the same set of streptococcal strains are presented, as generated by PROmer of MUMmer software and were visualized with the GenomeMatcher software (*see *Methods). Click here for file

Additional file 19 **Lengths of locally collinear blocks (LCBs) shared by the nine *Streptococcal *species**. Block lengths are taken from the *S. mutans *NN2025 genome. Lengths of LCBs were determined using SPRING software. Click here for file
