# Supplementary material for: Comparative genomic analyses of Streptococcus mutans provide insights into chromosomal shuffling and species-specific content
Source: BMC Genomics. 2009 Aug 5;10:358. doi: 10.1186/1471-2164-10-358 (PMC2907686; doi:10.1186/1471-2164-10-358)
Supplement: Additional file 1 — General features of S. mutans strains NN2025 and UA159. [file 1471-2164-10-358-S1.pdf]

Additional file 1. General features of *S. mutans* NN2025 and UA159

Features	Strains	
	NN2025	UA159[1]
Serotype	*c*	*c*
Length of sequence	2,013,587	2,030,921
G+C content	36.85%	36.83%
Open Reading frames		
Percentage of coding	85.18%	85.82%
Protein coding region	1895	1960
Average gene length (bp)	903.2	889.3
RNA		
ribosomal RNA	5	5
transfer RNA	65	65
Tansposon or Insertion sequence (IS)		
full length	6	7
partial	13	15

[1]The genomic sequences of *S. mutans* UA159 (NC004350) was obtained through the website of the National Center of Biological Information (NCBI, http://www.ncbi.nlm.nih.gov).
