# Supplementary material for: Comparative genomic analyses of Streptococcus mutans provide insights into chromosomal shuffling and species-specific content
Source: BMC Genomics. 2009 Aug 5;10:358. doi: 10.1186/1471-2164-10-358 (PMC2907686; doi:10.1186/1471-2164-10-358)
Supplement: Additional file 2 — S. mutans NN2025 specific ORFs. Different regions within the PCR region are shown in different colours and correspond to the regions shown in Figure 2. No coloring in the PCR region indicates the absence of the ORF in the regions in Figure 2. [file 1471-2164-10-358-S2.pdf]

Additonal file 2. *S. mutans* NN2025 specific ORFs

ORF	start	stop	A.A. length	gene	origin	E-value	paralog in UA159	PCR region
SmuNN2025.0046	54797	57226	810	ATP/GTP binding protein	*Fusobacterium nucleatum* ATCC 49256	1.00E-85		Region 1
SmuNN2025.0077	94136	94846	237	Putative methyltransferase	*Maricaulis maris* MCS10	1.00E-07	SMU.1367c	Region 2
SmuNN2025.0078	94824	95528	235	methyltransferase domain protein	marine gamma proteobacterium HTCC2080	9.00E-45		Region 2
SmuNN2025.0079	95572	96585	338	extracellular GAPDH	*S. mutans* UA159	0	SMU.360	Region 2
SmuNN2025.0080	96610	97086	159	putative autoinducer-2 production protein LuxS	*S. mutans* UA159	0	SMU.474	Region 2
SmuNN2025.0081	97472	98047	192	hypothetical protein		-		Region 2
SmuNN2025.0082	98137	98610	158	hypothetical protein L153086	*Lactococcus lactis* subsp. lactis Il1403	3.37E-48		Region 2
SmuNN2025.0083	98603	99775	391	Major facilitator superfamily MFS_1	*Lactobacillus reuteri* 100-23	4.00E-47		Region 2
SmuNN2025.0084	100178	100687	170	hypothetical protein SPs1818	*S. pyogenes* SSI-1	1.58E-48		Region 2
SmuNN2025.0085	101152	101460	103	hypothetical protein gbs1316	*S. agalactiae* NEM316	8.82E-32		Region 2
SmuNN2025.0086	101729	102130	134	site-specific recombinase, phage integrase family	*S. agalactiae* A909	1.79E-20		Region 2
SmuNN2025.0090	106161	106526	122	hypothetical protein	*S. mutans* UA159	-	SMU.281	Region 2
SmuNN2025.0091	106447	106707	87	hypothetical protein	*S. mutans* UA159	-	SMU.613	Region 2
SmuNN2025.0099	114611	114880	90	putative transposon fragment		-		
SmuNN2025.0242	268237	268590	118	hypothetical protein		-		
SmuNN2025.0243	269193	269336	48	hypothetical protein		-		
SmuNN2025.0254	276724	276963	80	hypothetical protein		-		
SmuNN2025.0255	276991	277167	59	hypothetical protein		-		
SmuNN2025.0272	295529	296275	249	hypothetical protein SSA_0700	*S. sanguinis* SK36	8.33E-19		
SmuNN2025.0317	345154	345264	37	hypothetical protein		-		
SmuNN2025.0322	349976	350134	53	hypothetical protein		-		Region 18
SmuNN2025.0323	350161	350313	51	MukA2	*S. mutans*	4.00E-21		Region 18
SmuNN2025.0324	350353	350508	52	MukA3	*S. mutans*	1.00E-22		Region 18
SmuNN2025.0325	350702	350863	54	MukA'	*S. mutans*	2.00E-23		Region 18
SmuNN2025.0326	350881	353667	929	putative salivaricin A modification enzyme	*S. pyogenes* SSI-1	2.00E-47		Region 18
SmuNN2025.0327	353690	355762	691	scnT-like protein	*S. mutans* UA159	-	SMU.1773	Region 18
SmuNN2025.0334	361808	361924	39	hypothetical protein	*S. mutans* UA159	-	SMU.1804c	
SmuNN2025.0342	366314	367153	280	hypothetical protein SE1476	*Staphylococcus epidermidis* ATCC 12228	1.00E-56		
SmuNN2025.0361	388071	388373	101	hypothetical protein		-		Region 17
SmuNN2025.0362	388377	388958	194	Conserved protein associated with flavoprotein oxygenase	*S. thermophilus* LMD-9	4.00E-50		Region 17
SmuNN2025.0364	391211	392221	337	conserved hypothetical protein	*Flavobacterium johnsoniae* UW101	0		Region 17
SmuNN2025.0365	392214	393311	366	hypothetical protein		-		Region 17
SmuNN2025.0366	393304	393822	173	restriction enzyme, beta subunit	*Haemophilus somnus* 129PT	4.00E-56		Region 17
SmuNN2025.0367	393885	394649	255	hypothetical protein		-		Region 17
SmuNN2025.0368	395028	398033	1002	hypothetical protein SSP0074	*Staphylococcus saprophyticus* ATCC 15305	0		Region 17
SmuNN2025.0370	400889	402010	374	deoxyguanosine triphosphate triphosphohydrolase	*Francisella tularensis* subsp. novicida U112	6.00E-71		Region 17
SmuNN2025.0371	402335	403447	371	hypothetical protein		-		Region 17
SmuNN2025.0372	403625	403849	75	hypothetical protein		-		Region 17
SmuNN2025.0373	403842	404126	95	Plasmid addiction system poison protein	*Fusobacterium nucleatum* ATCC 25586	-	SMU.40	Region 17
SmuNN2025.0484	508515	508814	100	putative peptidyl-prolyl cis-trans isomerase (fragment)	*S. mutans* UA159	-	SMU.488	
SmuNN2025.0529	552645	552839	65	hypothetical protein SPs0823	*S. pyogenes* SSI-1	4.00E-11		
SmuNN2025.0531	555061	556923	621	hypothetical protein EF2348	*Enterococcus faecalis* V583	5.00E-29		Region 16
SmuNN2025.0532	557360	558523	388	hypothetical protein pBT9727_0064	*Bacillus thuringiensis* serovar konkukian str. 97-27	0		Region 16
SmuNN2025.0533	558593	560461	623	hypothetical protein str0709	*S. thermophilus* CNRZ1066	5.00E-12		Region 16
SmuNN2025.0534	561219	561344	42	hypothetical protein		-		Region 16
SmuNN2025.0535	561345	561470	42	hypothetical protein		-		Region 16
SmuNN2025.0536	561345	561470	42	hypothetical protein		-		Region 16
SmuNN2025.0599	631778	631918	47	putative phenylalanyl-tRNA synthetase (fragment)	*S. mutans* UA159	-	SMU.1510	
SmuNN2025.0603	634842	637652	937	CRISPR-associated helicase	*Lactobacillus casei* ATCC 334	0		Region 19
SmuNN2025.0604	637645	639363	573	CRISPR-associated protein	*Lactobacillus casei* ATCC 334	7.00E-78		Region 19
SmuNN2025.0605	639424	639975	184	CRISPR-associated protein	*Lactobacillus casei* ATCC 334	1.00E-17		Region 19
SmuNN2025.0606	639959	641038	360	hypothetical protein Ldb0871	*Lactobacillus delbrueckii* ATCC 11842	6.00E-70		Region 19
SmuNN2025.0607	641044	641760	239	hypothetical protein jk0645	*Corynebacterium jeikeium* K411	1.00E-37		Region 19
SmuNN2025.0608	641811	642407	199	CRISPR-associated protein	*Lactobacillus casei* ATCC 334	2.00E-46		Region 19
SmuNN2025.0609	642410	643351	314	CRISPR-associated protein	*Lactobacillus casei* ATCC 334	0		Region 19
SmuNN2025.0610	643353	644255	301	DNA polymerase III subunit alpha	*S. mutans* UA159	-	SMU.123	Region 19
SmuNN2025.0629	668141	668296	52	hypothetical protein	-	-		
SmuNN2025.0640	678532	678744	71	Protein of unknown function DUF1706	*Clostridium* sp. OhILAs	1.00E-18		
SmuNN2025.0648	684747	684770	8	hypothetical protein	-	-		
SmuNN2025.0661	700255	700569	105	hypothetical protein SPs0155	*S. pyogenes* SSI-1	6.00E-20		
SmuNN2025.0690	732968	733309	114	hypothetical protein	*S. mutans* UA159	-	SMU.1411	
SmuNN2025.0698	743251	743442	64	hypothetical protein	-	-		Region 20
SmuNN2025.0700	744476	744913	146	hypothetical protein SAG0901	*S. agalactiae* 2603V/R	5.00E-25		Region 20
SmuNN2025.0701	744938	745294	119	hypothetical protein SAG0901	*S. agalactiae* 2603V/R	6.00E-23		Region 20
SmuNN2025.0702	745400	745573	58	hypothetical protein		-		Region 20
SmuNN2025.0703	745598	745828	77	hypothetical protein SAG0901	*S. agalactiae* 2603V/R	1.00E-10		Region 20
SmuNN2025.0704	746126	746554	143	hypothetical protein SAG0901	*S. agalactiae* 2603V/R	4.00E-23		Region 20
SmuNN2025.0705	746588	746881	98	hypothetical protein SAG0901	*S. agalactiae* 2603V/R	8.00E-16		Region 20
SmuNN2025.0706	746919	747206	96	hypothetical protein SAG0901	*S. agalactiae* 2603V/R	4.00E-17		Region 20
SmuNN2025.0707	747249	747476	76	hypothetical protein SAG0901	*S. agalactiae* 2603V/R	2.00E-13		Region 20
SmuNN2025.0708	747513	747674	54	hypothetical protein	-	-		Region 20
SmuNN2025.0709	747711	747995	95	hypothetical protein SAG0901	*S. agalactiae* 2603V/R	3.00E-17		Region 20
SmuNN2025.0710	748219	748323	35	hypothetical protein	-	-		Region 20
SmuNN2025.0711	748402	748611	70	hypothetical protein	-	-		Region 20
SmuNN2025.0732	769735	769983	83	hypothetical protein	*S. mutans* UA159	8.00E-06	SMU.1267c	Region 15
SmuNN2025.0734	770759	771379	207	hypothetical protein	-	-		Region 15
SmuNN2025.0781	820286	820486	67	hypothetical protein	-	-		
SmuNN2025.0795	830062	830409	116	transcriptional regulator	Bacteriophage EJ-1	2.00E-18		Region 14
SmuNN2025.0796	830892	834101	1070	putative type IIS restriction/modification enzyme	*Bacteroides thetaiotaomicron* VPI-5482	1.00E-94		Region 14
SmuNN2025.0797	834388	834606	73	putative type IIS restriction/modification enzyme	*Bacteroides thetaiotaomicron* VPI-5482	6.00E-21		Region 14
SmuNN2025.0798	834738	835313	192	site-specific recombinase, resolvase family	*Porphyromonas gingivalis* W83	2.00E-48		Region 14
SmuNN2025.0799	835300	838749	1150	helicase, SNF2/RAD54 family, putative	*Clostridium novyi* NT	7.00E-54		Region 14
SmuNN2025.0800	838783	838962	60	hypothetical protein	-	-		Region 14
SmuNN2025.0801	839057	839380	108	Plasmid maintenance system killer protein	*Lactobacillus brevis* ATCC 367	4.00E-26		Region 14
SmuNN2025.0802	839418	839711	98	Transcriptional regulator, xre family	*Lactobacillus brevis* ATCC 367	2.00E-17		Region 14
SmuNN2025.0812	847318	847884	189	hypothetical protein	*S. mutans* UA159	-	SMU.1243	Region 14
SmuNN2025.0837	870094	870255	54	putative ABC transporter, amino acid binding protein (fragme	*S. mutans* UA159	-	SMU.1217c	
SmuNN2025.0844	878285	879322	346	hypothetical protein	*S. mutans* UA159	-	SMU.1160c	
SmuNN2025.0876	917575	918273	233	putative transposase	*S. mutans* UA159	-	SMU.767	
SmuNN2025.0877	918390	918680	97	hypothetical protein	*S. dysgalactiae*	4.00E-41		
SmuNN2025.0889	931385	931834	150	hypothetical protein	-	-		
SmuNN2025.0896	936861	937115	85	putative ABC transporter, ATP-binding protein MutF (fragem	*S. mutans* UA159		SMU.654	
SmuNN2025.1022	1072168	1072398	77	putative DNA processing Smf protein	*S. mutans* UA159		SMU.1001	
SmuNN2025.1041	1090293	1090403	37	hypothetical protein	-	-		
SmuNN2025.1060	1109426	1110184	253	putative integrase	*S. mutans* UA159		SMU.191c	Region 21
SmuNN2025.1061	1110623	1110880	86	hypothetical protein	*S. agalactiae* H36B	4.00E-21		Region 21
SmuNN2025.1062	1110873	1111454	194	hypothetical protein gbs1316	*S. agalactiae* NEM316	9.00E-86		Region 21
SmuNN2025.1063	1111577	1111840	88	hypothetical protein SPs1820	*S. pyogenes* SSI-1	1.00E-18		Region 21
SmuNN2025.1064	1112193	1113269	359	hypothetical protein	-		SMU.1629c	Region 21
SmuNN2025.1065	1113269	1113583	105	hypothetical protein gbs1321	*S. agalactiae* NEM316	1.00E-44		Region 21
SmuNN2025.1066	1113814	1115052	413	hypothetical protein SPs1818	*S. pyogenes* SSI-1	8.00E-16		Region 21
SmuNN2025.1067	1115091	1116548	486	hypothetical protein AmetDRAFT_3489	*Alkaliphilus metalliredigenes* QYMF	0		Region 21
SmuNN2025.1068	1116561	1117253	231	hypothetical protein AmetDRAFT_3488	*Alkaliphilus metalliredigenes* QYMF	5.00E-36		Region 21
SmuNN2025.1069	1117262	1121956	1565	hypothetical protein pli0021	*Listeria innocua* Clip11262	0		Region 21
SmuNN2025.1117	1172733	1172849	39	hypothetical protein		-		
SmuNN2025.1123	1178122	1179489	456	hypothetical protein Bcer98DRAFT_2617	*Bacillus cereus* subsp. cytotoxis NVH 391-98	3.00E-87		Region 10
SmuNN2025.1124	1179650	1179955	102	putative ABC transporter, ATP-binding protein (fragment)	*S. mutans* UA159		SMU.413	Region 10
SmuNN2025.1139	1198756	1199454	233	putative transposase, ISSmu1	*S. mutans* UA159		SMU.767	
SmuNN2025.1216	1283570	1283698	43	hypothetical protein		-		
SmuNN2025.1233	1301702	1302310	203	hypothetical protein stu0695	*S. thermophilus* LMG 18311	4.00E-38		Region 22
SmuNN2025.1234	1302201	1303409	403	hypothetical protein STER_0734	*S. thermophilus* LMD-9	8.00E-77		Region 22
SmuNN2025.1253	1322699	1322887	63	hypothetical protein		-		
SmuNN2025.1254	1323667	1323801	45	hypothetical protein		-		
SmuNN2025.1263	1225354	1226259	302	putative transcriptional regulator; CpsY-like protein	*S. mutans* UA159		SMU.734	
SmuNN2025.1267	1228958	1229293	112	hypothetical protein	*S. mutans* UA159		SMU.730	
SmuNN2025.1270	1338115	1338225	37	hypothetical protein		-		
SmuNN2025.1283	1352908	1354011	368	putative ABC transporter, ATP-binding protein	*S. mutans* UA159		SMU.1163c	Region 9
SmuNN2025.1284	1354475	1356133	553	hypothetical protein		-		Region 9
SmuNN2025.1285	1356242	1357021	260	hypothetical protein		-		Region 9
SmuNN2025.1286	1357708	1357995	96	hypothetical protein		-		Region 9
SmuNN2025.1287	1358007	1358285	93	hypothetical protein BB14905_01970	*Bacillus* sp. B14905	2.00E-14		Region 9
SmuNN2025.1457	1537919	1538530	204	hypothetical protein Efae DRAFT_1703	*Enterococcus faecium* DO	2.00E-59		Region 23
SmuNN2025.1458	1538535	1539398	288	fructose-bisphosphate aldolase	*S. mutans* UA159		SMU.99	Region 23
SmuNN2025.1459	1539422	1539832	137	hypothetical protein	*Enterococcus faecium* DO	6.00E-27		Region 23
SmuNN2025.1460	1539852	1541141	430	ascorbate-specific PTS system enzyme IIC	*S. mutans* UA159		SMU.270	Region 23
SmuNN2025.1461	1541156	1541464	103	Phosphotransferase system, lactose/cellobiose-specific IIB sul	*Enterococcus faecium* DO	6.00E-29		Region 23
SmuNN2025.1462	1541464	1543545	694	putative transcriptional regulator	*S. mutans* UA159		SMU.272	Region 23
SmuNN2025.1565	1652559	1652918	120	hypothetical protein	*S. mutans* UA159		SMU.2125	
SmuNN2025.1566	1653054	1653719	222	hypothetical protein	*S. mutans* UA159		SMU.1602	
SmuNN2025.1602	1689952	1691931	660	RNA-directed DNA polymerase from retron ec67	*Listeria monocytogenes* str. 4b H7858	0		
SmuNN2025.1610	1696871	1697005	45	50S ribosomal protein L34	*S. mutans* UA159	2.00E-05		
SmuNN2025.1630	1715185	1715268	28	hypothetical protein		-		
SmuNN2025.1633	1717688	1718629	314	hypothetical protein L80315	*Lactococcus lactis* subsp. lactis Il1403	6.00E-84		
SmuNN2025.1634	1718786	1719328	181	hypothetical protein		-		
SmuNN2025.1635	1719364	1720047	228	hypothetical protein		-		
SmuNN2025.1636	1720133	1720687	185	hypothetical protein		-		
SmuNN2025.1645	1728755	1729279	175	Amidase from nicotinamidase family	*Clostridium acetobutylicum* ATCC 824	8.00E-37		Region 24
SmuNN2025.1647	1730403	1731596	398	S-adenosylmethionine synthetase	*S. mutans* UA159		SMU.1573	Region 24
SmuNN2025.1650	1733190	1734812	541	hypothetical protein DSY3668	*Desulfitobacterium hafniense* Y51	9.00E-17		Region 24
SmuNN2025.1651	1734902	1735603	234	putative ABC transporter; ATP-binding protein	*S. mutans* UA159		SMU.1366c	Region 24
SmuNN2025.1653	1737278	1738174	299	ABC-type MDR transport system, permease component	*Clostridium acetobutylicum* ATCC 824	4.00E-14		Region 24
SmuNN2025.1654	1738288	1739031	248	putative ABC transporter (SLS)	*S. pyogenes* SSI-1	5.00E-12		Region 24
SmuNN2025.1655	1739043	1739975	311	putative ABC transporter, ATP-binding protein			SMU.654	Region 24
SmuNN2025.1656	1740037	1741203	389	Macrolide-efflux protein	*Bacillus cereus* ATCC 14579	3.00E-15		Region 24
SmuNN2025.1657	1741211	1742002	264	methyltransferase	*Synechococcus* sp. RS9917	-		Region 24
SmuNN2025.1658	1742063	1746253	1397	putative bacitracin synthetase 1; BacA	*S. mutans* UA159		SMU.1342	Region 24
SmuNN2025.1684	1784372	1784653	94	hypothetical protein		-		
SmuNN2025.1729	1831455	1831595	47	hypothetical protein		-		
SmuNN2025.1732	1832776	1832964	63	hypothetical protein			SMU.224c	
SmuNN2025.1735	1834052	1834300	83	putative integrase (fragment)			SMU.221c	
SmuNN2025.1760	1854534	1855322	263	50S ribosomal protein L2	*S. pyogenes* SSI-1	0		
SmuNN2025.1761	1855385	1855684	100	50S ribosomal protein L23	*S. pyogenes* SSI-1	3.00E-45		
SmuNN2025.1769	1864572	1864910	113	small multidrug export protein-like	*S. agalactiae* 18RS21	1.00E-47		Region 25
SmuNN2025.1770	1865116	1866912	599	putative MDR permease; possible transmembrane efflux protein			SMU.1605	Region 25
SmuNN2025.1771	1867091	1867633	181	putative transcriptional regulator			SMU.1168	Region 25
SmuNN2025.1772	1867775	1868317	181	putative transcriptional regulator			SMU.38c	Region 25
SmuNN2025.1773	1868447	1868950	168	hypothetical protein SMU.39			SMU.39	Region 25
SmuNN2025.1774	1869139	1871880	914	putative polyketide synthase			SMU.1343c	Region 25
SmuNN2025.1775	1871884	1874958	1025	putative bacitracin synthetase 1; BacA			SMU.1342	Region 25
SmuNN2025.1776	1874965	1875411	149	2,4-diacetylphloroglucinol biosynthesis protein (phlB)	*Pseudomonas fluorescens* Pf-5	1.00E-23		Region 25
SmuNN2025.1777	1875412	1876623	404	2,4-diacetylphloroglucinol biosynthesis protein (phlC)	*Pseudomonas fluorescens* Pf-5	7.00E-65		Region 25
SmuNN2025.1778	1876638	1877654	339	PhlA	*Pseudomonas fluorescens*	5.00E-39		Region 25
SmuNN2025.1787	1891163	1891300	46	hypothetical protein		-		
SmuNN2025.1793	1900824	1901174	117	hypothetical protein			SMU.1566	
SmuNN2025.1833	1943229	1943291	21	hypothetical protein		-		
SmuNN2025.1834	1943434	1943661	76	hypothetical protein SPs0663	*S. pyogenes* SSI-1	3.00E-09		
SmuNN2025.1842	1955831	1956307	159	putative transposon fragment			SMU.767	
SmuNN2025.1843	1956510	1956785	92	hypothetical protein			SMU.766	
SmuNN2025.1844	1957297	1958046	250	conserved hypothetical protein	*Herpetosiphon aurantiacus* ATCC 23779	1.00E-13		
SmuNN2025.1862	1972916	1973359	148	putative purine-nucleoside phosphorylase (fragment)			SMU.2126c	
