# Supplementary material for: Comparative genomic analyses of Streptococcus mutans provide insights into chromosomal shuffling and species-specific content
Source: BMC Genomics. 2009 Aug 5;10:358. doi: 10.1186/1471-2164-10-358 (PMC2907686; doi:10.1186/1471-2164-10-358)
Supplement: Additional file 3 — S. mutans UA159-specific ORFs. Different regions within PCR region are shown in different colors and correspond to the regions shown in Figure 2. No coloring in the PCR resion indicates the absence of the ORF in the regions in Figure 2. [file 1471-2164-10-358-S3.pdf]

Additional file 3. *S. mutans* UA159-specific ORFs

ORF	start	stop	A.A. length	gene	origin	paralog in NN2025	PCR region	GI[a]	Blocks[b]
SMU.33	38633	38914	94	hypothetical protein				3	
SMU.44	47171	47782	204	hypothetical protein		SmuNN2025.0039		3	SMU.42-SMU.47
SMU.52	55428	56093	222	hypothetical protein C0370	*Plasmodium falciparum*		Region 1	3	
SMU.53	56109	56324	72	hypothetical protein		SmuNN2025.0039	Region 1	3	SMU.52-SMU.58
SMU.54	56331	57023	231	putative amino acid recemase Cj0085c	*Campylobacter jejuni* strain NCTC 11168		Region 1	3	SMU.52-SMU.58
SMU.55	57125	57388	88	hypothetical protein			Region 1	3	SMU.52-SMU.58
SMU.58	57639	58934	432	hypothetical ATP binding protein	*Methanococcus jannaschii*		Region 1	3	
SMU.92c	95724	95861	46	hypothetical protein		SmuNN2025.1730	Region 2		SMU.92-SMU.94
SMU.93c	95980	96090	37	hypothetical protein		SmuNN2025.1730	Region 2		SMU.92-SMU.94
SMU.94c	96291	96461	57	putative transposase fragment	*Streptococcus agalactiae*		Region 2		SMU.92-SMU.94
SMU.106c	107734	107970	79	putative transcription factor		SmuNN2025.0099		4	SMU.100-SMU.116
SMU.107	109108	109278	57	hypothetical protein					SMU.100-SMU.116
SMU.108	109280	109417	46	hypothetical protein	*Deinococcus radiodurans* strain R1				SMU.100-SMU.116
SMU.135	139471	140364	298	putative transcriptional regulator		SmuNN2025.1803	Region 3		SMU.135-SMU.143
SMU.136c	140554	140907	118	hypothetical protein	*Thermotoga maritima* strain MSB8		Region 3		SMU.135-SMU.143
SMU.138	142800	143744	315	malate permease	*Oenococcus oeni*	SmuNN2025.1114	Region 3		SMU.135-SMU.143
SMU.139	143865	145049	395	hypothetical protein *yvrK*	*Bacillus subtilis*		Region 3		SMU.135-SMU.143
SMU.140	145133	146428	432	putative glutathione reductase	*Staphylococcus sciuri*	SmuNN2025.1176	Region 3		SMU.135-SMU.143
SMU.141	146430	147131	234	hypothetical protein		SmuNN2025.1892	Region 3		SMU.135-SMU.143
SMU.149	152999	153301	101	putative transposase	*Bacillus halodurans*	SmuNN2025.1088	Region 4		SMU.149-SMU.153
SMU.150	153813	154016	68	bovicin 255 peptide precursor	*Streptococcus* sp. LRC 0255	SmuNN2025.0237	Region 4		SMU.149-SMU.153
SMU.151	154025	154261	79	Bacteriocin (Lactacin F subunit)	*Lactobacillus* sp.		Region 4		SMU.149-SMU.153
SMU.152	154456	154875	140	hypothetical protein			Region 4		SMU.149-SMU.153
SMU.153	155368	155469	34	hypothetical protein			Region 4		SMU.149-SMU.153
SMU.185	182383	182520	46	hypothetical protein					
SMU.191c	192029	193192	388	integrase	bacteriophage TPW22	SmuNN2025.1734	Region 5	6	SMU.191-SMU.226
SMU.193c	193199	193384	62	hypothetical protein			Region 5	6	SMU.191-SMU.226
SMU.194c	193388	193639	84	hypothetical protein 11 kDa protein; Bacteriophage P2 associated	*Escherichia coli*		Region 5	6	SMU.191-SMU.226
SMU.195c	193675	193935	87	similar to ORF 5 of bacteriophage SPP1	Bacteriophage SPP1		Region 5	6	SMU.191-SMU.226
SMU.196c	193953	195050	366	transfer protein	*Streptococcus thermophilus*	SmuNN2025.1178	Region 5	6	SMU.191-SMU.226
SMU.197c	195072	197498	809	hypothetical protein L1156.02	*Leishmania major*		Region 5	6	SMU.191-SMU.226
SMU.198c	197498	200041	848	similar to ORF16 *Enterococcus faecalis* transposon Tn916	*Enterococcus faecalis*		Region 5	6	SMU.191-SMU.226
SMU.199c	200061	200441	127	hypothetical protein *yddD*	*Bacillus subtilis*		Region 5	6	SMU.191-SMU.226
SMU.200c	200470	200697	76	hypothetical protein			Region 5	6	SMU.191-SMU.226
SMU.201c	200710	201690	327	similar to ORF13 *Enterococcus faecalis* transposon Tn916	*Enterococcus faecalis*		Region 5	6	SMU.191-SMU.226
SMU.202c	201706	202179	158	hypothetical protein			Region 5	6	SMU.191-SMU.226
SMU.204c	202302	202430	43	hypothetical protein			Region 5	6	SMU.191-SMU.226
SMU.205c	202493	202765	91	hypothetical protein			Region 5	6	SMU.191-SMU.226
SMU.206c	202868	203269	134	hypothetical protein			Region 5	6	SMU.191-SMU.226
SMU.207c	203232	204464	411	similar to ORF20 *Enterococcus faecalis* transposon Tn916	*Enterococcus faecalis*		Region 5	6	SMU.191-SMU.226
SMU.208c	204699	206423	575	putative transfer protein	*Streptococcus thermophilus*	SmuNN2025.0486	Region 5	6	SMU.191-SMU.226
SMU.209c	206439	206864	142	similar to ORF22 and 23 *Enterococcus faecalis* transposon	*Enterococcus faecalis*		Region 5	6	SMU.191-SMU.226
SMU.210c	206888	207199	104	hypothetical protein			Region 5	6	SMU.191-SMU.226
SMU.211c	207419	207862	148	hypothetical protein			Region 5	6	SMU.191-SMU.226
SMU.212c	207865	208113	83	hypothetical protein BBI01 - Lyme disease spirochete plasmid I/lp28-4	*Borrelia burgdorferi*		Region 5	6	SMU.191-SMU.226
SMU.213c	208136	208327	64	hypothetical protein			Region 5	6	SMU.191-SMU.226
SMU.214c	208355	208663	103	hypothetical protein			Region 5	6	SMU.191-SMU.226
SMU.215c	208672	208872	67	hypothetical protein			Region 5	6	SMU.191-SMU.226
SMU.216c	208952	209266	105	transcription factor A	*Rattus norvegicus*		Region 5	6	SMU.191-SMU.226
SMU.217c	209386	209913	176	maltose ABCtransporter, permease protein	*Thermotoga maritima*		Region 5	6	SMU.191-SMU.226
SMU.218	210660	211013	118	putative transcriptional regulator	*Streptococcus agalactiae* COH1	SmuNN2025.1201	Region 5	6	SMU.191-SMU.226
SMU.219	211020	211451	144	hypothetical protein gbs0474	*Streptococcus agalactiae* NEM316		Region 5	6	SMU.191-SMU.226
SMU.220c	211770	212222	151	hypothetical protein			Region 5	6	SMU.191-SMU.226
SMU.223c	213755	213856	34	hypothetical protein	*Bacillus halodurans*		Region 5	6	SMU.191-SMU.226
SMU.225c	214336	214449	38	hypothetical protein		SmuNN2025.1731	Region 5	6	SMU.191-SMU.226
SMU.261c	250989	251945	319	transcriptional regulator	*Enterococcus faecalis* V583	SmuNN2025.1484	Region 6		SMU.261-SMU.226
SMU.262	252184	253203	340	putrescine carbamoyltransferase	*Enterococcus faecalis* V583	SmuNN2025.1410	Region 6		SMU.261-SMU.226
SMU.263	253301	254659	453	probable amino acid permease	*Pseudomonas aeruginosa*		Region 6		SMU.261-SMU.226
SMU.264	254669	255778	370	hypothetical protein	*Pseudomonas aeruginosa*		Region 6		SMU.261-SMU.226
SMU.265	255810	256760	317	carbamate kinase			Region 6		SMU.261-SMU.226
SMU.340	321953	322087	45	50S ribosomal protein L34, homologous to *E.coli rpmH*	*Pseudomonas putida*				
SMU.373	355112	355855	248	biosynthesis methyltransferase related PAB2213	*Pyrococcus abyssi*		Region 7		SMU.372-SMU.381
SMU.375	356563	357471	303	hypothetical protein *yydH*	*Bacillus subtilis*		Region 7		SMU.372-SMU.381
SMU.376	357487	358725	413	adenosylmethionine-8-amino-7-oxononanoate aminotransferase	*Bacillus thuringiensis* str. Al Hakam	SmuNN2025.1325	Region 7		SMU.372-SMU.381
SMU.378	358920	359159	80	hypothetical protein			Region 7		SMU.372-SMU.381
SMU.379	359137	359274	46	hypothetical protein			Region 7		SMU.372-SMU.381
SMU.390	366854	367012	53	hypothetical protein					
SMU.436c	406250	407086	279	putative transposase fragment	*Streptococcus thermophilus* LMD9	SmuNN2025.1360			
SMU.444	414522	414629	36	hypothetical protein					
SMU.451	421261	421365	35	hypothetical protein					
SMU.512c	480285	480740	152	methyltransferase	*S. pyogenes* MGAS9429	SmuNN2025.1368			
SMU.513	481136	481369	78	hypothetical protein		SmuNN2025.1370			
SMU.529	496432	496545	38	hypothetical protein					
SMU.565c	529007	529843	279	putative transposase fragment	*Streptococcus thermophilus* LMD9	SmuNN2025.1360			
SMU.566c	529837	530355	173	putative transposase fragment	*Streptococcus thermophilus* LMD9	SmuNN2025.1361			
SMU.590c	549699	549899	67	putative transposase fragment	*Streptococcus suis* 89/1591	SmuNN2025.1360			
SMU.604	562431	562706	92	hypothetical protein *yddK*	*Bacillus subtilis*		Region 8	9	SMU.604-SMU.606
SMU.605	562712	563263	184	hypothetical protein *yddK*	*Bacillus subtilis*		Region 8	9	SMU.604-SMU.606
SMU.606	563276	564523	416	hypothetical protein MJ0875	*Methanococcus jannaschii*		Region 8	9	SMU.604-SMU.606
SMU.613	576338	576616	93	hypothetical protein		SmuNN2025.1685			
SMU.614	576710	576850	47	calcium channel protein gamma chain, DHP-sensitive	*Oryctolagus cuniculus*				
SMU.620	579129	579287	53	hypothetical protein					
SMU.642	601170	601451	94	hypothetical protein		SmuNN2025.0818			
SMU.654	614935	615639	235	putative ATP-binding protein, MutF	*Streptococcus mutans*	SmuNN2025.0328			SMU.651-SMU.658
SMU.655	615641	615889	83	putative MutE	*Streptococcus mutans*				SMU.651-SMU.658
SMU.656	615874	616389	172	putative MutE	*Streptococcus mutans*				SMU.651-SMU.658
SMU.681	644674	644859	62	hypothetical protein TDE0674	*Treponema denticola* ATCC 35405		Region 9		SMU.681-SMU.687
SMU.682	645162	647720	853	hypothetical protein 84.2 kDa	*Escherichia coli*		Region 9		SMU.681-SMU.687
SMU.683	647736	651149	1138	putative ATP-binding protein	*Archaeoglobus fulgidus*		Region 9		SMU.681-SMU.687
SMU.684	651153	651842	230	hypothetical protein *yobL*	*Bacillus subtilis*		Region 9		SMU.681-SMU.687
SMU.685	651839	652240	134	calreticulin	*Schistosoma mansoni*		Region 9		SMU.681-SMU.687
SMU.687c	652698	652832	45	hypothetical protein			Region 9		SMU.681-SMU.687
SMU.722	681658	681837	60	hypothetical protein					
SMU.732	689393	690043	217	Predicted membrane protein	*Clostridium acetobutylicum* ATCC 824				
SMU.738	692912	693046	45	hypothetical protein		SmuNN2025.1263			
SMU.748	702575	702751	59	hypothetical protein					
SMU.750c	703472	703579	36	hypothetical protein					
SMU.791c	741866	742105	80	hypothetical protein					
SMU.804	749791	751005	405	hypothetical protein	*Plasmodium yoelii*				SMU.803-SMU.804
SMU.811	757851	758147	99	hypothetical protein					
SMU.812	758147	758260	38	hypothetical protein aq_022	*Aquifex aeolicus*				
SMU.847c	796972	797376	135	hypothetical protein					
SMU.875c	825849	826466	206	transposase fragment	*Bacillus thuringiensis*	SmuNN2025.1141			SMU.876-SMU.888
SMU.892	846893	848704	604	putative restriction endonuclease	*Xylella fastidiosa*		Region 10		SMU.891-SMU.896
SMU.893	848735	849880	382	anticodon nuclease NMB0832	*Neisseria meningitidis* MC58		Region 10		SMU.891-SMU.896
SMU.895	849954	850226	91	possible DNA-damage-inducible protein	*Xylella fastidiosa*		Region 10		SMU.891-SMU.896
SMU.896	850213	850491	93	hypothetical protein , *yafQ*	*Escherichia coli*		Region 10		SMU.891-SMU.896
SMU.897	850494	853541	1016	putative helicase subunits (*hsdR*)	*Escherichia coli*		Region 10		
SMU.925	879393	879863	157	putative immunity protein, BlpL-like	*S. pneumoniae* TIGR4	SmuNN2025.0238			
SMU.948	897864	898034	57	hypothetical protein					
SMU.959c	908541	908795	85	hypothetical protein					
SMU.982	927460	928017	186	putative glucan binding protein D, fragment	*S. mutans*	SmuNN2025.1042			SMU.980-SMU.992
SMU.1024c	976530	976691	54	hypothetical protein		SmuNN2025.1360	Region 11	16	
SMU.1025	976838	977263	142	probable transcription regulator	*Streptococcus criceti*		Region 11	16	
SMU.1026	977310	977690	127	hypothetical protein			Region 11	16	SMU.1026-SMU.1032
SMU.1027	977808	978446	213	putative transcription regulator, TetR family	*Thermoto gamaritima*		Region 11	16	SMU.1026-SMU.1032
SMU.1028	978482	979516	345	putative hydrolase or acyltransferase	*Streptomyces coelicolor*		Region 11	16	SMU.1026-SMU.1032
SMU.1029	980040	980396	119	hypothetical protein	Transposon		Region 11	16	SMU.1026-SMU.1032
SMU.1030	980476	980706	77	putative polyribonucleotide nucleotidyltransferase; Tn916 ORF8-like	*Enterococcus faecalis*,DS16		Region 11	16	SMU.1026-SMU.1032
SMU.1031	981073	981276	68	putative transposon excisionase; Tn916 ORF1-like, Tn1545	*Enterococcus faecalis*		Region 11	16	SMU.1026-SMU.1032
SMU.1047c	995546	995653	36	hypothetical protein					
SMU.1056	1001046	1001165	40	hypothetical protein					
SMU.1141c	1082159	1082437	93	hypothetical protein *yktA*	*Bacillus subtilis*				
SMU.1147c	1086868	1087053	62	hypothetical protein					
SMU.1155	1092763	1093218	152	hypothetical protein			Region 12		
SMU.1156c	1092916	1093263	116	ADP-ribosyltransferase (NAD+; poly (ADP-ribose) polymerase	Mus musculus		Region 12		
SMU.1157c	1093482	1096667	1062	hypothetical protein, *Bacillus anthracis* virulence plasmid pXO1,	*Bacillus anthracis*		Region 12	10	
SMU.1205c	1146796	1147284	163	CG13992 gene product	*Drosophila melanogaster*		Region 13		SMU.1205-SMU.1209
SMU.1206c	1147387	1147944	186	hypothetical protein contains similarity to (p)ppGpp synthase	*Arabidopsis thaliana*		Region 13		SMU.1205-SMU.1209
SMU.1207	1148062	1148667	202	similar to mobilization/cell filamentation proteins	Plasmid pNZ4000		Region 13		SMU.1205-SMU.1209
SMU.1208c	1148712	1149467	252	nonmuscle myosin II heavy chain A	*Xenopus laevis*		Region 13		SMU.1205-SMU.1209
SMU.1209c	1149743	1151083	447	similar to SMC domain protein	*Clostridium beijerinckii* NCIMB 8052		Region 13		SMU.1205-SMU.1209
SMU.1230c	1170442	1170918	159	hypothetical protein ORF N043	*Staphylococcus aureus*				
SMU.1231c	1171037	1171132	32	hypothetical protein					
SMU.1255c	1189531	1189788	86	hypothetical protein	*Methanococcus jannaschii*		Region 14		SMU.1254-SMU.1259
SMU.1256c	1189999	1190163	55	hypothetical protein			Region 14		SMU.1254-SMU.1259
SMU.1257c	1190156	1190827	224	hypothetical protein EF1889	*Enterococcus faecalis*		Region 14		SMU.1254-SMU.1259
SMU.1258c	1190950	1191612	221	possible restriction endonuclease	*Lactococcus lactis* subsp. *cremoris*		Region 14		SMU.1254-SMU.1259
SMU.1259	1191675	1192001	109	possible restriction endonuclease	*Lactococcus lactis* subsp. *cremoris*		Region 14		
SMU.1262c	1192812	1194152	447	putative transmembrane protein MinC	*Nostoc* sp. PCC9229				SMU.1262-SMU.1264
SMU.1310	1234816	1234953	46	hypothetical protein				11	
SMU.1329c	1252672	1253145	158	putative transposase fragment	*Streptococcus criceti*	SmuNN2025.1730	Region 15	12	SMU.1329-SMU.1363
SMU.1330c	1253191	1253331	47	putative transposase fragment		SmuNN2025.0321	Region 15	12	SMU.1329-SMU.1363
SMU.1331c	1253406	1253951	182	putative transposase fragment	*Streptococcus suis* 89/1591	SmuNN2025.0321	Region 15	12	SMU.1329-SMU.1363
SMU.1332c	1254039	1254302	88	putative transposase fragment	*Streptococcus suis* 89/1591	SmuNN2025.0321	Region 15	12	SMU.1329-SMU.1363
SMU.1335c	1255804	1256787	328	utative enoyl-(acyl-carrier-protein) reductase	*Clostridium beijerinckii*	SmuNN2025.0381	Region 15	12	SMU.1329-SMU.1363
SMU.1336	1256800	1257738	313	bacillaene synthesis	*Bacillus amyloliquefaciens* FZB42	SmuNN2025.0382	Region 15	12	SMU.1329-SMU.1363
SMU.1337c	1257767	1258516	250	hypothetical protein		SmuNN2025.0628	Region 15	12	SMU.1329-SMU.1363
SMU.1338c	1258545	1259699	385	possible multi drug-efflux transporter	*Thermotoga maritima*		Region 15	12	SMU.1329-SMU.1363
SMU.1339	1259790	1264157	1456	non-ribosomal peptide synthetase F	*Brevibacillus texasporus*	SmuNN2025.1658	Region 15	12	SMU.1329-SMU.1363
SMU.1340	1264255	1269141	1629	gramicidin S synthase 2	*Bacillus brevis*	SmuNN2025.1658	Region 15	12	SMU.1329-SMU.1363
SMU.1341c	1269141	1272830	1230	Gramicidin S synthetase 2	*Aneurinibacillus migulanus*	SmuNN2025.1660	Region 15	12	SMU.1329-SMU.1363
SMU.1344	1284428	1285645	406	acyl carrier protein	*Streptococcus pneumoniae*	SmuNN2025.0382	Region 15	12	SMU.1329-SMU.1363
SMU.1347c	1288285	1290627	781	hypothetical protein		SmuNN2025.0223	Region 15	12	SMU.1329-SMU.1363
SMU.1348c	1290632	1291333	234	ABC transporter	*Clostridium acetobutylicum* ATCC 824	SmuNN2025.0879	Region 15	12	SMU.1329-SMU.1363
SMU.1349	1291686	1292261	192	transcriptional regulator (TetR/AcrR family)	*Aquifex aeolicus*		Region 15	12	SMU.1329-SMU.1363
SMU.1351	1292444	1292812	123	putative transposase fragment	*Lactococcus lactis*		Region 15	12	SMU.1329-SMU.1363
SMU.1352	1292790	1293113	108	putative transposase	*Lactococcus lactis*		Region 15	12	SMU.1329-SMU.1363
SMU.1353	1293138	1293293	52	hypothetical protein		SmuNN2025.1139	Region 15	12	SMU.1329-SMU.1363
SMU.1354c	1293679	1293939	87	hypothetical protein		SmuNN2025.1139	Region 15	12	SMU.1329-SMU.1363
SMU.1355c	1293954	1294247	98	hypothetical protein		SmuNN2025.1360	Region 15	12	SMU.1329-SMU.1363
SMU.1356c	1294262	1294552	97	putative transposase fragment, protein pXO1-128	*Bacillus anthracis*		Region 15	12	SMU.1329-SMU.1363
SMU.1357	1294643	1294879	79	putative transposase fragment	*Streptococcus pyogenes*		Region 15	12	SMU.1329-SMU.1363
SMU.1358	1294890	1294991	34	putative transposase fragment	*Streptococcus pyogenes*		Region 15	12	SMU.1329-SMU.1363
SMU.1359	1295127	1295237	37	hypothetical protein			Region 15	12	SMU.1329-SMU.1363
SMU.1360c	1295272	1295400	43	hypothetical protein			Region 15	12	SMU.1329-SMU.1363
SMU.1361c	1295378	1295959	194	transcriptional regulator	*Listeria monocytogenes*	SmuNN2025.1720	Region 15	12	SMU.1329-SMU.1363
SMU.1365c	1297315	1299660	782	probable permiase, ABC transporter	*Methanobrevibacter smithii* ATCC 35061	SmuNN2025.0223	Region 15	12	SMU.1329-SMU.1363
SMU.1366c	1299662	1300363	234	putative ABC transporter	*Clostridium acetobutylicum* ATCC 824	SmuNN2025.0879	Region 15	12	SMU.1329-SMU.1363
SMU.1368	1301468	1301587	40	hypothetical protein		SmuNN2025.0147	Region 15	12	SMU.1329-SMU.1363
SMU.1369	1301828	1301950	41	hypothetical protein			Region 15	12	SMU.1329-SMU.1363
SMU.1372c	1303560	1303802	81	IS1223 hypothetical 20.7 kD protein (ORFA)	*Lactobacillus johnsonii*		Region 15	12	SMU.1329-SMU.1363
SMU.1373c	1303978	1304160	61	hypothetical protein HI0112	*Haemophilus influenzae* Rd		Region 15	12	SMU.1329-SMU.1363
SMU.1374	1304544	1304747	68	hypothetical protein			Region 15	12	SMU.1329-SMU.1363
SMU.1379	1307343	1307861	173	transposon ISSsa1	*Strepcoccus sanguis* SK36	SmuNN2025.1361		12	
SMU.1399	1327999	1328319	107	hypothetical protein					
SMU.1407c	1336342	1337178	279	putative transposase	*Strepcoccus thermophilus* LMD-9	SmuNN2025.1360			
SMU.1408c	1337172	1337690	173	putative transposase	*Strepcoccus thermophilus* LMD-9	SmuNN2025.1361			
SMU.1456c	1387243	1387371	43	hypothetical protein					
SMU.1459c	1388991	1389164	58	hypothetical protein					
SMU.1462c	1390759	1391862	368	putative oxidoreductase, *yurR*	*Bacillus subtilis*				
SMU.1505c	1430750	1430869	40	hypothetical protein		SmuNN2025.0594			
SMU.1553c	1478460	1478654	65	hypothetical protein					
SMU.1574c	1501433	1502491	353	hypothetical protein BB0007	*Borrelia burgdorferi*		Region 16		SMU.1574-SMU.1577
SMU.1575c	1502821	1503105	95	helicase-like protein	*Arabidopsis thaliana*		Region 16		SMU.1574-SMU.1577
SMU.1576c	1503086	1505134	683	hypothetical protein PFB0195c	*Plasmodium falciparum*		Region 16		SMU.1574-SMU.1577
SMU.1577c	1505191	1508940	1250	hypothetical protein *yobI*	*Bacillus subtilis*		Region 16		SMU.1574-SMU.1577
SMU.1579	1510124	1510324	67	hypothetical protein PGUG_03393	*Pichia guilliermondii*				
SMU.1584c	1512917	1514686	590	myosin-cross reactive antigen	*Lactobacillus acidophilus* NCFM	SmuNN2025.1454			SMU.1584-SMU.1585
SMU.1585c	1514805	1515329	175	putative transcriptional regulator, TetR family	*Alkaliphilus metalliredigens* QYMF	SmuNN2025.1455			SMU.1584-SMU.1585
SMU.1610	1540373	1540519	49	50S ribosomal protein L33					
SMU.1638c	1561426	1561587	54	hypothetical protein					
SMU.1648c	1568916	1569161	82	hypothetical protein					
SMU.1750c	1661570	1661680	37	hypothetical protein		SmuNN2025.0374	Region 17		SMU.1750-SMU.1776
SMU.1753c	1662432	1662725	98	hypothetical protein	*Bacillus halodurans*		Region 17		SMU.1750-SMU.1776
SMU.1754c	1662736	1663131	132	hypothetical protein	*Bacillus halodurans*		Region 17		SMU.1750-SMU.1776
SMU.1755c	1663207	1663506	100	hypothetical protein	*Bacillus halodurans*		Region 17		SMU.1750-SMU.1776
SMU.1757c	1663475	1663759	95	hypothetical protein	*Bacillus halodurans*		Region 17		SMU.1750-SMU.1776
SMU.1758c	1663756	1664400	215	hypothetical protein	*Bacillus halodurans*		Region 17		SMU.1750-SMU.1776
SMU.1760c	1664429	1665304	292	hypothetical protein	*Bacillus halodurans*		Region 17		SMU.1750-SMU.1776
SMU.1761c	1665308	1666717	470	hypothetical protein	*Bacillus halodurans*		Region 17		SMU.1750-SMU.1776
SMU.1762c	1666677	1667240	188	hypothetical protein	*Bacillus halodurans*		Region 17		SMU.1750-SMU.1776
SMU.1763c	1667240	1667989	250	hypothetical protein	*Bacillus halodurans*		Region 17		SMU.1750-SMU.1776
SMU.1764c	1668084	1670492	803	ATP dependent RNA hilicase	*Streptococcus pyogenes*	SmuNN2025.0603	Region 17		SMU.1750-SMU.1776
SMU.1766c	1672007	1672159	51	hypothetical protein, *yle*	*Agrobacterium tumefaciens*		Region 17		SMU.1750-SMU.1776
SMU.1767c	1672216	1672407	64	acetohydroxy acid synthase large subunit	*Buchnera aphidicola*		Region 17		SMU.1750-SMU.1776
SMU.1768c	1672391	1672696	102	cell growth regulatory protein *mazE*	*Escherichia coli*		Region 17		SMU.1750-SMU.1776
SMU.1771c	1675930	1676238	103	hypothetical protein	*Archaeoglobus fulgidus*		Region 17		SMU.1750-SMU.1776
SMU.1772c	1676210	1676620	137	hypothetical protein, tail protein	*Streptococcus* phage Cp-1		Region 17		SMU.1750-SMU.1776
SMU.1773c	1676613	1677548	312	hypothetical protein ORF2136	*Marchantia polymorpha*		Region 17		SMU.1750-SMU.1776
SMU.1775c	1678588	1678767	60	hypothetical protein			Region 17		SMU.1750-SMU.1776
SMU.1776c	1678998	1679243	82	DNA-damage-inducibile protein *dinJ*	*Escherichia coli*		Region 17		SMU.1750-SMU.1776
SMU.1804c	1704149	1704304	52	hypothetical protein					SMU.1804-SMU.1818
SMU.1808c	1707319	1707414	32	T13262 integrase -Lactococcus lactis phage BK5-T	*Lactococcus lactis* phage BK5-T				SMU.1804-SMU.1818
SMU.1813	1712141	1712251	37	putative transposase fragment	*Bacillus halodurans*				SMU.1804-SMU.1818
SMU.1818c	1715576	1715749	58	hypothetical protein	*Lactococcus lactis*				SMU.1804-SMU.1818
SMU.1832	1727988	1728152	55	hypothetical protein					
SMU.1891c	1781567	1781749	61	hypothetical protein					
SMU.1893c	1781999	1782835	279	transposase	*Strepcoccus thermophilus* LMD-9	SmuNN2025.1360			
SMU.1894c	1782829	1783347	173	transposase	*Strepcoccus thermophilus* LMD-9	SmuNN2025.1361			
SMU.1896c	1783662	1783964	101	hypothetical protein		SmuNN2025.0252			SMU.1896-SMU.2054
SMU.1899	1786364	1786489	42	hypothetical protein		SmuNN2025.0250		15	SMU.1896-SMU.2054
SMU.1903c	1787987	1788139	51	hypothetical protein				15	SMU.1896-SMU.2054
SMU.1908c	1790689	1790853	55	hypothetical protein				15	SMU.1896-SMU.2054
SMU.2003a	1882208	1882324	39	50S ribosomal protein L36					SMU.1896-SMU.2054
SMU.2048	1922836	1922988	51	hypothetical protein					SMU.1896-SMU.2054
SMU.2053c	1925407	1925499	31	hypothetical protein		SmuNN2025.1796			SMU.1896-SMU.2054
SMU.2076c	1950643	1950771	43	hypothetical protein					
SMU.2096c	1965640	1965864	75	hypothetical protein contains similarity to AAA-type ATPase gene_id:MYH19.21	Arabidopsis thaliana				
SMU.2104a	1975771	1975953	61	50S ribosomal protein L32					
SMU.2105	1976160	1976285	42	hypothetical protein, probable ribonuclease Cj0631c	*Campylobacter jejuni*				
SMU.2107c	1976672	1976941	90	hypothetical protein, NADH dehydrogenase subunit	*Alligator mississippiensis*				
SMU.2124	1989375	1989572	66	hypothetical protein					
SMU.2131	1995815	1995979	55	hypothetical protein					
SMU.2136c	1999951	2000127	59	hypothetical protein					

a: Genomic Islands predicted by the Oral Pathogen Sequence Database, http//www.oralgen.lanl.gov/. b: Blocks of open reading frames absent in one or more strain of *S. mutans* predicted by Waterhouse et al. (Ref).
