# Supplementary material for: Comparative genomic analyses of Streptococcus mutans provide insights into chromosomal shuffling and species-specific content
Source: BMC Genomics. 2009 Aug 5;10:358. doi: 10.1186/1471-2164-10-358 (PMC2907686; doi:10.1186/1471-2164-10-358)
Supplement: Additional file 4 — Long-PCR analyses of genomic rearrangement region and insertion/deletion regions of S. mutans strains. [file 1471-2164-10-358-S4.pdf]

Additional file 4. Long-PCR analyses of genomic rearrangement region and insertion/deletion regions of *S. mutans* strains.

Strain	STs	Inversion	Region 1[b] Variable region 1	Region 2[c] Variable region 2	Region 3[d] UA159-specific 1	Region 4[e] UA159-specific 2	Region 5[f] UA159-specific 3 (TnSmu1)	Region 6[g] UA159-specific 4	Region 7[h] UA159-specific 5	Region 8[i] UA159-specific 6	Region 9[j] Variable region 3	Region 10[k] Variable region 4	Region 11[l] UA159-specific 7	Region 12[m] UA159-specific 7	Region 13[n] UA159-specific 8	Region 14[o] Variable region 5	Region 15[p] Variable region 6 (TnSmu2)	Region 16[q] Variable region 7	Region 17[r] Variable region 8	Region 18[s] NN2025-specific 1 (ScnT)	Region 19[t] NN2025-specific 2 (CRISPR 1)	Region 20[u] NN2025-specific 3 (CRISPR 2)	Region 21[v] NN2025-specific 4 (*Lla* cluster)	Region 22[w] NN2025-specific 5	Region 23[x] NN2025-specific 6	Region 24[y] NN2025-specific 7 (BacA cluster 1)	Region 25[z] NN2025-specific 8 (BacA cluster 2)
UA159	1	-	M	L	H	H	-	H	H	H	H	H	H	H	H	L	-	H	A	L	L	+	L	L	L	L	L
NN2025	70	+	L	H	L	L	H	L	L	L	L	L	L	L	L	H	+	M	B	H	H	+	H	H	H	H	H
NN2099	44	+	M	L	H	L	L	L	L	L	L	H	L	H	L	L	+	M	A	L	L	+	L	H	L	H	H
NN2093	54	-	-	-	L	L	-	L	L	L	L	-	L	L	L	-	-	-	-	L	L	-	L	L	L	L	H
MT8148	63	+	M	L	H	L	H	H	L	H	H	H	L	L	L	L	+	M	A	L	H	+	L	H	H	H	H
NN2004	33	+	M	L	H	H	-	L	L	H	L	H	L	L	H	L	-	M	-	L	L	+	L	H	H	H	H
NN2085	39	+	H	L	L	H	-	H	L	L	H	H	L	L	L	H	+	M	A	H	L	+	L	L	H	H	H
NN2087	56	+	M	-	H	L	-	L	L	H	L	L	L	L	L	H	+	M	B	L	L	+	L	H	H	H	H
NN2089	42	-	-	-	H	H	-	-	-	-	L	H	L	L	H	-	-	L	-	-	L	-	-	H	L	L	H
NN2037	10	-	-	H	H	L	-	-	L	H	L	-	-	L	H	-	-	-	-	L	L	+	L	L	L	L	H
NN2044	22	+	M	H	H	H	H	L	H	H	L	H	L	-	L	H	+	M	A	H	H	+	L	H	H	H	H
NN2054	19	+	M	-	H	L	H	H	L	H	H	H	L	L	L	L	+	M	A	L	H	+	L	L	H	H	H
NN2076	75	-	M	H	L	H	H	L	L	H	L	H	L	L	L	-	-	M	-	L	L	+	L	L	H	H	H
NN2042	10	+	H	H	H	H	H	H	L	L	H	H	L	L	L	L	+	-	A	H	H	+	L	H	H	H	H
NN2053	76	+	M	L	L	L	H	L	L	H	L	L	L	L	L	H	+	M	B	-	L	+	L	H	H	H	L
NN2072	69	+	M	L	H	L	H	H	L	H	H	-	M	L	H	-	+	-	-	-	L	+	L	L	L	L	H
NN2165	57	+	M	H	H	L	-	H	L	H	L	H	L	M	H	-	+	M	-	L	L	+	L	L	L	L	H
NN2007	35	+	M	L	H	H	H	H	L	H	H	H	M	L	L	-	+	L	A	-	L	+	L	L	L	H	H
NN2117	48	+	M	H	H	H	H	L	L	H	L	H	-	L	H	-	+	H	B	L	L	+	L	L	H	L	H
NN2138-2	45	+	M	-	H	H	H	H	L	-	H	H	L	L	L	H	+	M	A	-	L	+	-	L	L	H	H
NN2168M-5	49	-	-	H	L	L	L	L	L	H	L	L	-	L	H	-	-	-	-	L	L	-	L	L	L	L	H
NN2121	85	+	M	H	H	H	L	L	L	H	L	H	L	-	L	L	+	M	A	L	H	+	L	H	H	H	H
NN2431M-2	86	+	M	H	H	H	-	L	H	H	L	H	L	L	L	L	+	M	-	L	L	+	L	H	H	H	H
TW295	80	+	M	H	H	L	-	L	L	H	L	H	M	L	L	L	+	H	B	L	L	+	L	H	H	H	H
TW871	79	+	M	H	H	L	H	H	L	H	H	H	H	M	L	L	+	M	A	L	L	+	L	H	H	H	H
NN2011	47	-	M	H	H	H	H	L	L	H	L	L	H	L	H	H	+	M	-	L	L	+	L	L	H	H	H
NN2111	67	+	M	H	H	H	H	L	L	H	L	L	H	L	H	-	+	M	-	L	L	+	L	L	H	H	H
NN2323M1	66	+	M	H	H	H	H	H	L	L	H	L	H	L	H	L	+	M	A	L	L	+	L	H	H	H	H
NN2193-1	16	-	M	H	H	L	L	H	H	H	H	L	L	M	H	-	+	H	B	L	L	+	L	L	H	H	H
NN2105	68	+	M	H	H	L	-	H	L	H	H	L	H	L	H	-	+	M	B	L	L	+	L	L	H	H	H
OR22P1	87	+	H	L	H	L	H	H	L	H	H	L	H	L	H	-	+	L	-	L	L	+	L	H	L	H	H
LJ1	17	+	M	H	H	H	-	L	L	H	H	H	H	L	H	-	-	M	A	L	H	+	L	H	H	H	H
LJ2	40	+	H	L	H	H	H	L	L	H	H	H	L	M	H	H	+	M	A	L	L	+	L	L	H	H	H
LJ3	5	+	M	H	H	L	-	H	H	L	L	H	L	L	L	H	+	M	A	H	L	+	L	H	H	H	H
LJ4	7	+	M	H	L	L	H	L	L	H	L	L	L	L	L	H	+	M	B	L	L	+	L	H	H	H	L
LJ5	14	+	L	L	H	L	H	H	H	H	L	H	L	L	L	-	+	-	A	L	L	+	L	L	H	H	H
LJ11	25	+	M	L	H	H	H	L	L	H	L	H	L	L	L	H	+	H	A	L	H	+	L	H	H	H	H
LJ12	25	+	M	L	H	H	H	L	L	H	L	H	L	L	L	H	+	H	A	L	H	+	L	H	H	H	H
LJ13	58	+	H	L	H	H	H	L	H	H	H	H	L	L	L	H	+	H	A	L	L	+	L	H	L	H	H
LJ14	52	+	M	H	H	H	-	L	L	L	H	H	L	H	L	L	+	H	B	L	L	+	L	H	H	H	H
LJ16	43	+	M	L	H	H	H	L	L	H	L	H	L	L	L	L	+	L	A	L	L	+	L	L	L	H	H
LJ17	63	+	M	H	H	L	H	H	L	H	H	H	L	L	L	L	+	M	A	L	H	+	L	H	H	H	H
LJ18	77	+	M	H	H	L	L	L	H	H	L	H	L	L	L	H	+	M	A	L	H	+	L	H	H	H	H
LJ19	71	+	M	H	H	L	H	L	H	H	L	H	L	L	L	H	+	M	A	L	L	+	L	H	H	H	H
LJ20	27	+	M	H	H	L	H	L	L	L	H	H	L	L	L	H	+	M	B	H	L	+	H	L	H	H	H
LJ22	82	+	L	L	H	H	H	L	L	H	L	L	L	L	L	L	+	M	B	L	H	+	L	H	H	H	H
LJ23	88	+	H	H	L	L	H	H	L	H	L	L	H	H	L	H	+	M	-	L	L	+	L	L	H	H	H
LJ24	34	+	L	H	H	H	L	H	L	L	H	H	M	L	L	L	+	M	A	L	L	+	L	L	L	H	H
LJ25	89	+	H	L	H	H	L	-	L	L	L	H	L	L	L	L	+	M	A	L	L	+	L	L	L	H	H
LJ26	51	+	-	H	H	H	H	L	L	H	L	H	L	L	L	L	+	M	A	L	L	+	L	L	L	H	H
LJ27	90	+	M	L	H	H	H	L	L	H	L	H	M	L	L	L	+	M	A	-	L	+	L	L	L	H	H
LJ30	8	+	H	L	H	H	H	L	L	H	L	H	L	L	H	H	+	M	B	-	L	+	L	H	H	L	H
LJ31	8	+	H	L	H	H	H	L	L	H	H	H	L	L	H	H	+	M	B	-	L	+	L	H	H	L	H
LJ32	37	+	M	H	H	L	H	H	L	H	H	H	M	L	H	H	+	M	-	L	L	+	L	L	L	L	H
LJ15	62	+	H	H	H	L	H	L	L	H	H	H	L	H	L	L	+	H	B	L	L	+	L	H	H	H	H
LJ29	12	+	H	H	H	L	L	L	L	H	H	L	L	L	L	L	-	M	B	L	L	+	L	L	H	H	H
SA22	21	+	M	L	H	H	L	H	H	L	L	H	H	L	L	L	+	M	A	L	L	+	L	L	H	H	H
SA31	36	+	M	H	H	H	H	H	H	H	H	L	L	L	L	-	+	H	-	L	L	+	L	L	H	H	H
SA51	2	+	M	H	H	H	H	H	H	L	H	H	H	L	L	L	+	-	-	L	L	+	L	L	L	H	H
SA53	55	+	M	H	H	H	L	H	L	H	H	H	M	M	L	L	+	M	B	L	L	+	L	L	L	H	H
SA72	38	+	M	H	H	L	H	H	H	H	H	L	L	H	L	L	+	H	B	L	L	+	L	L	H	H	H
LJ36	72	+	M	H	H	L	H	-	H	H	L	H	L	L	H	H	+	H	A	L	H	+	L	H	H	L	H
LJ59	78	+	M	H	H	L	H	H	H	L	L	H	L	M	L	H	+	H	A	L	H	+	L	H	H	H	H
LJ64	74	+	M	H	H	L	H	L	H	L	L	H	L	L	L	H	+	M	A	L	H	+	L	H	H	H	H
SA12	50	+	H	L	H	H	H	L	L	H	L	H	L	L	L	L	+	M	-	H	L	+	L	L	L	H	H
SA13	18	+	M	H	H	L	H	H	L	H	L	L	L	H	H	H	+	H	B	L	L	+	L	H	H	H	L
SA14	53	-	M	H	L	L	H	H	L	H	H	H	L	L	H	-	-	L	-	L	L	+	L	H	H	H	H
SA15	41	+	H	L	H	L	H	L	L	H	H	H	L	L	H	H	+	L	-	L	L	+	L	H	L	H	H
SA16	28	+	L	H	H	L	H	H	H	H	H	H	L	L	H	L	+	H	A	L	L	+	L	H	L	H	H
SA17	60	+	M	H	H	L	H	L	L	H	H	H	H	L	H	-	+	L	-	L	L	+	L	L	L	H	H
SA18	32	+	M	H	H	L	H	L	L	H	L	H	L	L	H	L	+	H	B	L	H	+	L	H	H	H	L
TW964	91	+	M	H	H	L	H	H	L	L	L	H	M	L	H	-	+	M	-	L	L	+	L	L	H	H	H
TW1378	13	+	M	L	H	H	H	L	L	H	H	H	L	L	H	H	+	M	A	H	H	-	L	L	L	H	H
V1	63	+	-	H	H	L	H	-	L	H	L	-	L	L	L	-	-	-	-	L	L	+	L	H	L	H	H
P1	4	-	-	-	L	H	H	-	L	H	L	-	L	L	L	-	-	L	-	L	L	-	L	H	L	L	H
MT4065	64	+	M	H	H	H	H	-	L	H	L	H	L	L	H	L	+	M	A	L	H	+	L	H	L	H	H
MT4071	92	+	M	-	H	H	-	-	H	H	L	H	L	M	H	L	+	-	A	L	L	+	L	H	H	H	H
MT4076	2	+	M	H	H	H	L	-	H	L	H	H	H	H	L	L	-	-	A	L	L	+	L	L	L	H	H
MT4078	20	+	M	H	H	L	L	L	L	H	L	H	M	L	L	L	+	M	A	L	H	+	L	L	L	H	H
MT4087	29	+	M	-	H	H	H	L	L	L	H	H	M	L	L	L	+	M	A	L	L	+	L	H	L	L	H
MT4093	24	+	M	-	H	H	H	L	L	H	L	H	L	L	L	H	-	H	-	L	H	+	H	H	H	L	L
MT4112	61	+	M	-	H	H	H	L	L	H	L	H	H	L	L	L	+	-	A	L	L	+	L	L	L	H	H
MT4164	65	+	M	L	H	H	L	L	L	L	L	H	L	L	L	H	+	H	-	L	H	+	L	H	H	L	H
MT4117	81	+	M	H	H	H	H	-	L	H	L	H	L	L	L	L	+	-	A	L	H	+	L	H	H	H	H
MT4119	59	+	M	L	H	H	L	L	L	H	H	H	L	L	H	H	+	M	A	L	L	+	L	L	L	H	H
MT4217	6	+	M	H	H	H	H	H	L	H	L	H	L	L	L	H	+	M	A	L	H	+	L	H	H	H	H
MT4245	72	+	H	H	H	H	H	H	H	H	L	H	L	L	L	H	+	H	A	L	H	+	L	H	H	H	H
MT4274	30	+	M	H	H	H	H	H	H	H	L	H	L	L	L	H	+	M	A	L	H	+	L	H	H	H	H
MT4279	83	+	M	H	H	H	H	H	H	H	L	H	L	L	H	H	+	M	A	L	H	+	L	H	H	L	H
MT4293	73	-	M	H	H	H	H	L	H	H	L	H	L	L	H	H	+	M	A	L	H	+	L	H	H	L	H
MT4368	10	+	H	-	H	H	L	-	L	H	H	H	L	L	L	L	+	-	-	L	H	+	L	H	H	H	H
MT4369	11	-	H	L	H	H	H	-	L	H	H	H	L	L	H	L	-	-	-	L	H	+	L	H	H	H	H
MT4333	46	+	M	H	H	H	H	H	L	H	H	H	L	L	L	H	+	M	A	L	L	+	L	L	L	H	H
LJ34	3	+	M	H	H	H	H	H	H	H	L	H	L	L	H	H	+	M	B	L	H	+	L	H	H	L	H
LJ50	78	+	M	H	L	H	H	L	L	L	L	L	L	L	L	H	+	M	-	H	L	+	L	H	H	H	L
MT4251	85	-	M	-	H	H	H	L	L	H	H	H	L	L	L	H	+	-	-	L	L	+	L	L	H	L	H
MT4348	15	-	M	-	H	H	-	-	H	H	L	H	L	L	L	L	+	-	-	L	L	+	L	H	H	H	H

a: + shows a single PCR product by long PCR, - shows no PCR products. H, M, L shows a different PCR product with the same primer pairs. b: H; 5.0 kb, M; 4.4 kb, L; 3.4 kb. c: H;19.6 kb, L; 11.1 kb. d: H; 8.3 kb, L: 0.3 kb. e: H; 4.6 kb, L; 1.5 kb. f: H; 6.8 kb, L; 0.2 kb. g: H; 7.4 kb, L; 1.0 kb. h: H; 4.7 kb, L; 2. 5 kb. i; H; 8.9 kb, L; 0.4 kb. j: H; 8.8 kb, L; 2.2 kb. k: H3.2 kb, L; 2.0 kb. l: H; 8.0 kb, M; 3.3 kb, L; 2.0 kb. m: H; 6.2 kb, M; 4.2 kb, L; 3.7 kb. n: H, 4.7 kb, L; 1.7 kb. o: H; 11.1 kb, L; 4.8 kb. p: + shows 4.0 kb of PCR product. q: H; 6.7 kb, M; 4.0 kb, L; 2.2 kb. r: The PCR products were digested with EcoRI, then separated on agarose gel. The digested products were divided into two (A and B) patterns. s: H; 12.0 kb, L; 0.2 kb. t: The size of PCR products in region 14 were variable because this region includes the palindromic repeats, therefore + shows a single PCR products and – shows no PCR products. u: H; 13.0 kb, L; 0.15 kb. v: H; 2.5 kb, L; 0.5 kb. w: H; 6.0 kb, L; 0.2 kb. x: H; 30 kb, L; 0.2 kb. y: H; 14.7 kb, L; 1.0 kb.
