# Supplementary material for: Comparative genomic analyses of Streptococcus mutans provide insights into chromosomal shuffling and species-specific content
Source: BMC Genomics. 2009 Aug 5;10:358. doi: 10.1186/1471-2164-10-358 (PMC2907686; doi:10.1186/1471-2164-10-358)
Supplement: Additional file 5 — Primers used for detection of rearrangement and strain-specific regions in S. mutans strains. Each strain-specific region is visualized as a black bar in Figure 2. [file 1471-2164-10-358-S5.pdf]

Additional file 5. Primers used for detection of rearrangement and strain-specific regions in *S. mutans* strains.

Primers	Sequence (5' to 3')	strains			
		NN2025		UA159	
		Position	length (bp)	Position	length (bp)
Inv-F	TGAGACATCATGATATGAGTTAGCTTTCTGGGTTG	179172	7685	183252	-
Inv-R	TTCTAATAGTTTAAGGAGAAACCATTAGCCCATTC	186857		1872996	
1-F	GAAATCTCGGTCATTATCTCAGGAAACGGCAAGGA	54292	3378	54886	4447
1-R	TAGGAATCTCACCCAACTCAGCCCAAGCCTCGTCA	57670		59333	
2-F	GCTCAAACATGACATTGCAATGAAAAAAGCGGTT	93859	19612	95518	11112
2-R	CAATTGCTTTTTCAATTTTAGCCTTGTTCTCTTC	113471		1065943	
3-F	GCTTTATCATGAGCACACGCAAGAAGAAGCACAC	146017	342	139116	8271
3-R	CTGAAAGGATATCAGTCGATCGTTGTCCAGCACG	146359		147387	
4-F	ACCAGCTAATCCACGTCAGCCTTACATTGAAGAG	151698	1491	152726	4543
4-R	TTGTTTGGCAACCTGACCGATTTCTACAGCCAGC	153189		157269	
5-F	TTTCAGTAAGTGTATGACCATAAACTTGTATCAAC	1831007	3209	192079	-
5-R	ACAACATTTCGGCAGGATATATCAAGATTATTGTC	1834216		215913	
6-F	CTAATAATAAATCAGGAAAACAGCAATCTGATCCTC	1662257	185	361002	6815
6-R	GCCCAACTTGATATGAAAATTGAAGTAATTGATATTG	1662442		354187	
7-F	TGCTCTTTATGCTATTTGGTTAGCACTAGCTGAAC	1796341	957	250556	7350
7-R	CTTGGGATTTCTATTTTTGAAACAGCAGCCAG	1795384		257906	
8-F	GGGATCAAATTCCATGAAATTGGCCCAAGTTGTG	1455232	2516	562273	4659
8-R	TCTGGATATTTATCTGCAAACCAATGAAGAGAAA	1457748		566932	
9-F	CGAAAATGTTTAGCAATACGACTAATATCGGATTCCGT	1379519	401	653116	8974
9-R	GCAAGAAAGAAATTGCTGCTATCATTACACATGCCGC	1379920		644142	
10-F	AAATTCCTTTTGACGTACAATTTCCATTGTTATCTCC	1177987	2196	853745	8758
10-R	GGCAGTGGTGATTAGTGTTTGTGTTCTACGTGTGA	1180183		844987	
11-F	GACAGGAAATTGCTGAATATGCAAGAAGTGAAGA	1040018	2025	976311	7932
11-R	TTGATGAGAGTTGTCTTACCTGCACCATTTTTACC	1042043		984243	
12-F	CTTTCTTTAATGTTGTCTTCGTTGGTCTCACTTT	928319	3692	1082086	17126
12-R	TTGACAGTCTTGAAAGTATATTTAATATTTTGGA	932011		1099212	
13-F	GCGAATATCTGGCAAAGCCCGTTCTTGAATGATG	878103	1701	1146502	4660
13-R	CCGCGTCAACGTCCTCATGGGCGACAAGGTTCCG	879804		1151162	
14-F	GGTGCTAAGTTTATCGTAGAAACAGTAGAAAACTTGAG	840421	11201	1189483	4873
14-R	TATGTCAAGTCCATCAAGACAGATTGTGACCAAGAC	829220		1194356	
15-F	GTAGATAAAACAATCATTGAGTGATGAGATATGCTATCTAG	767717	3965	1308755	-
15-R	CCTGAAGAACGCCCTTCTTCAACAGAAGCACGTAATGAT	771682		1251782	
16-F	GGACTGAGATTCTTAGGAGATTTATGAAAAAGAAAAGTTTTAGG	560644	6794	1501239	7845
16-R	AAGTTTTAGTGAAGAAAGTATCAAGGACGATGGAAGAAG	553850		1509084	
17-F	CCTCAGATGTTAATTGACTTTACTATTTTTATCCAAGCC	405031	17697	1661300	18142
17-R	CAAATAGATCAAACAAGCAACAATAGAAAAAAGGAACTG	387334		1679442	
18-F	ACCAATAGCATTTACTTCTGGTTCAGACAATCCT	347827	8921	1709286	3024
18-R	GTTAAATTAAGGAAAATGTCTTCAAGAGTCTCTG	356748		1712310	
19-F	GGAAAGCAAGACGACAAAATTGAATCGAGCTTTCATTACG	633464	11972	142916	-
19-F	AATCGGTCTAACATCACAAGTCTATATGTATAGGTAAAACATG	645436		1428994	
20-F	TGATGGGAAAGGAGTTCCTGAGTTTAGGATATGA	742128	6635	1327698	2411
20-R	TACAATTGAAGTCATCGGAACGGTCATTACCTAA	748763		1330109	
21-F	ATTCCAATTTCAGAGAAGGTACAATTGAAACGCCAA	1109226	13002	909045	157
21-R	AAGCTAAACTTGAAGAAGCTGGAGCAACTGTTACTC	1122228		908888	
22-F	GGTGCCTGTTGATCATAGATCGTATCATCAACATC	1301439	2454	723807	463
22-R	TGTTGGTCATTCAATTCTTCTGTATGTTGATGTG	1303893		724270	
23-F	TCCACGTCGCATGACTAAAGAACTCATTAATCAAGTC	1543743	6011	479396	198
23-R	TATAGCACAGAGAGTATGCCTTGGAATACCAATAGG	1537732		479594	
24-F	ACTTAATAAAAATCGGATGGAATAACTAACATCTTC	1729566	30421	293187	182
24-R	CCAGCAGCCCTAAGCGATGTAAGCTGTGTGCTCTA	1759987		293369	
25-F	TGCCAGCTTTCTAAATAGGAACTTTCAAAACGAGC	1863722	14657	1899549	964
25-R	GCTTATATGGAATCCGTAAATGGGAAAGTGATTAG	1878379		1900513	
