# Supplementary material for: Comparative genomic analyses of Streptococcus mutans provide insights into chromosomal shuffling and species-specific content
Source: BMC Genomics. 2009 Aug 5;10:358. doi: 10.1186/1471-2164-10-358 (PMC2907686; doi:10.1186/1471-2164-10-358)
Supplement: Additional file 6 — Characteristics of S. mutans reference strains and clinical isolates used in this study. [file 1471-2164-10-358-S6.pdf]

Additional file 6. Characteristics of *S. mutans* reference strains and clinical isolates used in this study.

Strain	serotype	STs[a]	Isolated year	Source	Origin	child/adult	Adhesion[b]	*cnm*[c]	reference
NN2025	*c*	67	2002	Oral	Japan	child	97.5	-	Nakano (2007)
UA159	*c*	1	1980s	Oral	United States	child	86	-	Ajdic et al (2002)
MT8148	*c*	63	1980s	Oral	Japan	child	91.5	-	Ooshima(1983)
MT4065	*c*	64	1980s	Oral	Japan	child	83.8	-	Nakano (2007)
MT4071	*c*	92	1980s	Oral	Japan	child	81.4	-	Nakano (2007)
MT4076	*c*	2	1980s	Oral	Japan	child	88.9	-	Nakano (2007)
MT4078	*c*	20	1980s	Oral	Japan	child	55.2	-	Nakano (2007)
MT4083	*c*	26	1980s	Oral	Japan	child	80.8	+	Nakano (2007)
MT4087	*c*	29	1980s	Oral	Japan	child	86.6	-	Nakano (2007)
MT4093	*c*	24	1980s	Oral	Japan	child	91.5	-	Nakano (2007)
MT4112	*c*	61	1980s	Oral	Japan	child	90.5	-	Nakano (2007)
MT4118	*c*	65	1980s	Oral	Japan	child	85.5	-	Nakano (2007)
MT4164	*c*	23	1980s	Oral	Japan	child	89.6	-	Nakano (2007)
MT4117	*e*	81	1980s	Oral	Japan	child	77.2	-	Nakano (2007)
MT4119	*e*	59	1980s	Oral	Japan	child	78.6	-	Nakano (2007)
MT4217	*e*	6	1980s	Oral	Japan	child	85.4	-	Nakano (2007)
MT4245	*e*	72	1980s	Oral	Japan	child	76.2	-	Nakano (2007)
MT4274	*e*	30	1980s	Oral	Japan	child	83.8	-	Nakano (2007)
MT4278	*e*	84	1980s	Oral	Japan	child	77.9	-	Nakano (2007)
MT4279	*e*	83	1980s	Oral	Japan	child	83.1	-	Nakano (2007)
MT4293	*e*	73	1980s	Oral	Japan	child	6.2	-	Nakano (2007)
MT4368	*e*	10	1980s	Oral	Japan	child	70.9	-	Nakano (2007)
MT4369	*e*	11	1980s	Oral	Japan	child	82.4	-	Nakano (2007)
MT4251	*f*	85	1980s	Oral	Japan	child	89.4	-	Nakano (2007)
MT4333	*f*	46	1980s	Oral	Japan	child	89.1	+	Nakano (2007)
MT4348	*f*	15	1980s	Oral	Japan	child	81.9	+	Nakano (2007)
NN2098	*c*	31	2002	Oral	Japan	child	83.3	-	Nakano (2004)
NN2099	*c*	44	2002	Oral	Japan	child	79.1	-	Nakano (2004)
NN2092	*c*	9	2002	Oral	Japan	child	89.1	-	Nakano (2004)
NN2093	*c*	54	2002	Oral	Japan	child	95	-	Nakano (2004)
NN2004	*c*	33	2002	Oral	Japan	child	90.2	-	Nakano (2004)
NN2085	*c*	39	2002	Oral	Japan	child	91.7	-	Nakano (2004)
NN2087	*e*	56	2002	Oral	Japan	child	93.6	-	Nakano (2004)
NN2089	*e*	42	2002	Oral	Japan	child	83.1	-	Nakano (2004)
NN2037	*e*	10	2002	Oral	Japan	child	92.3	-	Nakano (2004)
NN2044	*e*	22	2002	Oral	Japan	child	88.1	-	Nakano (2004)
NN2054	*e*	1980s	2002	Oral	Japan	child	86.2	-	Nakano (2004)
NN2076	*e*	75	2002	Oral	Japan	child	90	-	Nakano (2004)
NN2042	*e*	10	2002	Oral	Japan	child	89.2	-	Nakano (2004)
NN2053	*e*	76	2002	Oral	Japan	child	96.2	-	Nakano (2004)
NN2072	*f*	69	2002	Oral	Japan	child	77.6	+	Nakano (2004)
NN2165	*f*	57	2002	Oral	Japan	child	77.1	+	Nakano (2004)
NN2007	*f*	35	2002	Oral	Japan	child	82.9	+	Nakano (2004)
NN2117	*f*	48	2002	Oral	Japan	child	89.2	+	Nakano (2004)
NN2138	*f*	45	2002	Oral	Japan	child	45	+	Nakano (2007)
NN2168M-5	*c*	49	2002	Oral	Japan	adult	85.3	-	Nakano (2007)
NN2121	*f*	85	2002	Oral	Japan	child	90	+	Nakano (2004)
NN2431M-2	*f*	86	2002	Oral	Japan	adult	83.9	+	Nakano (2007)
NN2011	*k*	47	2002	Oral	Japan	child	83.1	-	Nakano (2004)
NN2111	*k*	67	2002	Oral	Japan	child	80.8	-	Nakano (2004b)
NN2323M-1	*k*	66	2002	Oral	Japan	adult	84.3	-	Nakano (2007)
NN2193-1	*k*	16	2002	Oral	Japan	child	77.4	-	Nakano (2007)
NN2105	*k*	68	2002	Oral	Japan	child	85.6	-	Nakano (2004b)
LJ1	*c*	17	2006	Oral	Japan	adult	88.3	-	Nakano (2007)
LJ2	*e*	40	2006	Oral	Japan	child	74.9	-	Nakano (2007)
LJ3	*e*	5	2006	Oral	Japan	adult	81.8	-	Nakano (2007)
LJ4	*e*	7	2006	Oral	Japan	child	91.6	-	Nakano (2007)
LJ7	*f*	14	2006	Oral	Japan	adult	86.4	+	Nakano (2007)
LJ11	*c*	25	2006	Oral	Japan	adult	93.1	-	Nakano (2007)
LJ12	*c*	25	2006	Oral	Japan	child	88.9	-	Nakano (2007)
LJ13	*c*	58	2006	Oral	Japan	adult	80.3	-	Nakano (2007)
LJ14	*c*	52	2006	Oral	Japan	adult	85.4	-	Nakano (2007)
LJ16	*c*	43	2006	Oral	Japan	adult	75.5	-	Nakano (2007)
LJ17	*c*	63	2006	Oral	Japan	child	82.2	-	Nakano (2007)
LJ18	*e*	77	2006	Oral	Japan	adult	80.8	-	Nakano (2007)
LJ19	*e*	71	2006	Oral	Japan	child	82.6	-	Nakano (2007)
LJ20	*c*	27	2006	Oral	Japan	adult	84.6	+	Nakano (2007)
LJ22	*c*	82	2006	Oral	Japan	child	87.7	-	Nakano (2007)
LJ23	*k*	88	2006	Oral	Japan	adult	81.3	+	Nakano (2007)
LJ24	*f*	34	2006	Oral	Japan	adult	78.9	+	Nakano (2007)
LJ25	*c*	89	2006	Oral	Japan	child	70.9	-	Nakano (2007)
LJ26	*c*	51	2006	Oral	Japan	adult	91.4	+	Nakano (2007)
LJ27	*c*	90	2006	Oral	Japan	child	79.4	-	Nakano (2007)
LJ30	*c*	8	2006	Oral	Japan	adult	87.3	-	Nakano (2007)
LJ31	*c*	8	2006	Oral	Japan	child	88.3	-	Nakano (2007)
LJ32	*f*	37	2006	Oral	Japan	adult	84.1	+	Nakano (2007)
LJ15	*c*	62	2006	Oral	Japan	child	80.7	-	Nakano (2007)
LJ29	*c*	12	2006	Oral	Japan	child	82.2	-	Nakano (2007)
LJ34	*e*	3	2006	Oral	Japan	child	83.9	-	Nakano (2007)
LJ36	*e*	72	2006	Oral	Japan	child	83.1	-	Nakano (2007)
LJ50	*e*	78	2006	Oral	Japan	child	92.1	-	Nakano (2007)
LJ59	*e*	72	2006	Oral	Japan	child	87.2	-	Nakano (2007)
LJ64	*e*	74	2006	Oral	Japan	adult	84.2	-	Nakano (2007)
SA22	*f*	21	1988	Oral	Finland	child	69.7	-	Nakano (2007)
SA31	*k*	36	1990	Oral	Finland	child	67.6	-	Nakano (2007)
SA51	*f*	2	1988	Oral	Finland	child	69.9	-	Nakano (2007)
SA53	*k*	55	early 90s	Oral	Finland	child	85.4	+	Nakano (2007)
SA72	*k*	38	early 90s	Oral	Finland	child	65.5	-	Nakano (2007)
SA12	*c*	50	after 2000	IE	Finland	adult	75.4	-	Nakano (2007)
SA13	*c*	18	after 2000	Bacteremia	Finland	adult	84.8	-	Nakano (2007)
SA14	*c*	53	after 2000	IE	Finland	adult	90.8	-	Nakano (2007)
SA15	*e*	41	after 2000	Bacteremia	Finland	adult	75.2	-	Nakano (2007)
SA16	*e*	28	after 2000	IE	Finland	adult	81.8	-	Nakano (2007)
SA17	*c*	60	after 2000	Bacteremia	Finland	adult	91.1	-	Nakano (2007)
SA18	*c*	32	after 2000	IE	Finland	adult	89.5	-	Nakano (2007)
TW295	*k*	80	after 2000	Bacteremia	Japan	adult	70.4	+	Fujiwara (2001)
TW871	*k*	79	1992	IE	Japan	adult	75.1	+	Fujiwara (2001)
TW964	*f*	91	1993	IE	Japan	adult	83.7	-	Fujiwara (2001)
TW1378	*e*	13	1993	IE	Japan	adult	88.6	-	Fujiwara (2001)
V1	*c*	63	2005	IE	Japan	adult	24.1	-	Nomura (2006)
P1	*c*	4	2005	Oral	Japan	adult	80.7	-	Nomura (2006)
OR22P1	*k*	87	2005	Oral	Japan	child	79.6	-	Nakano (2007)

a: Sequence Types (STs) were determined by Multilocus sequence methods (Nakano et al, 2007). b: Sucrose-dependent adhesion (Adhesion)(%) of organism was calculated from the percent aodherence was defined as 100% OD_{550} (adhesive cells)/OD_{550}(total cells). c: Distribution of collagen-binding protein (*cnm*) gene was determined by PCR.
