# Supplementary figures and images for: Comparative genomic analyses of Streptococcus mutans provide insights into chromosomal shuffling and species-specific content
Source: BMC Genomics. 2009 Aug 5;10:358. doi: 10.1186/1471-2164-10-358 (PMC2907686; doi:10.1186/1471-2164-10-358)

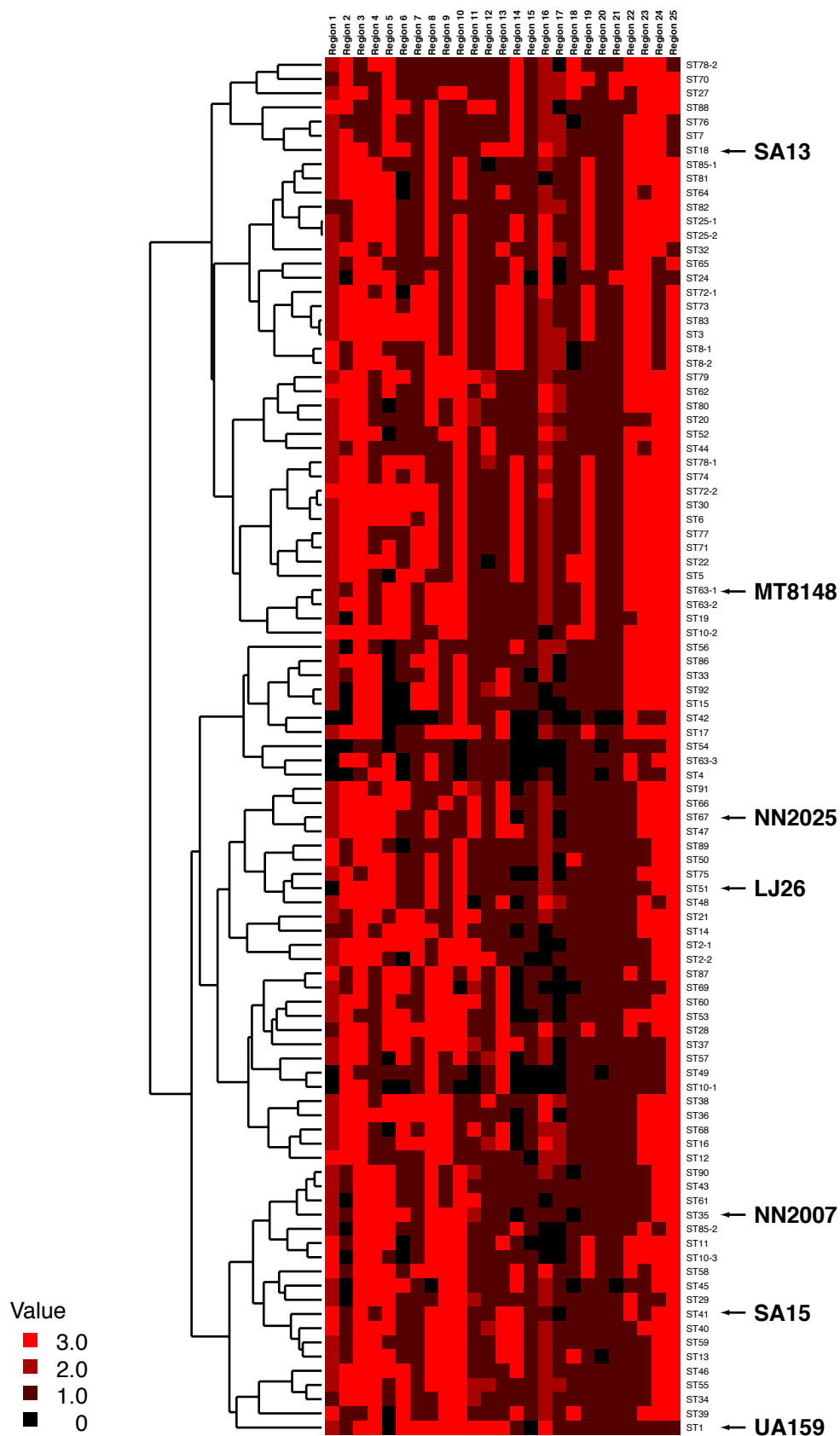

Supplement: Additional file 7 — Cluster analysis based on strain-specific regions showing the relationship between S. mutans strain NN2025, UA159 and 95 clinical isolates. Long-PCR results were converted to numerical values according to the length of the PCR products, then complete linkage clustering was performed on CLUSTER software and visualized with Java Tree view software (contrast value; 0 to 3.0). [file 1471-2164-10-358-S7.pdf]

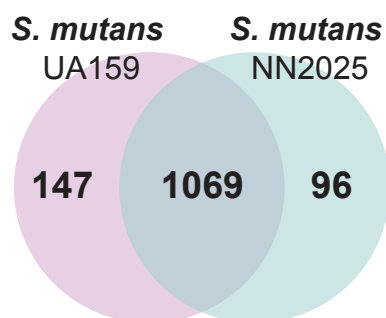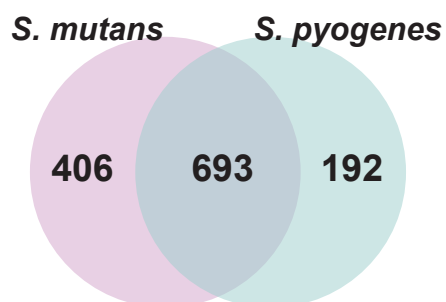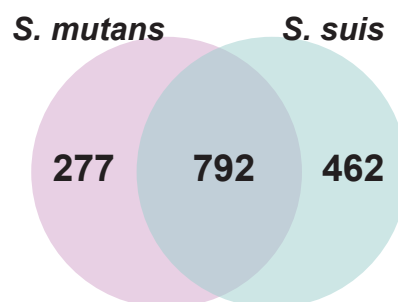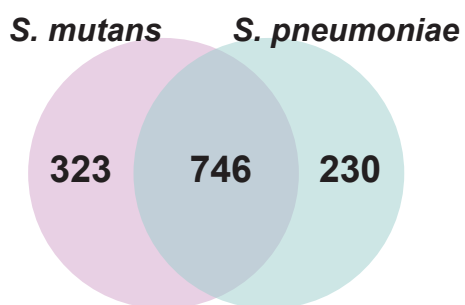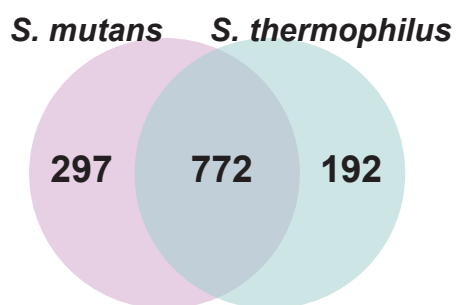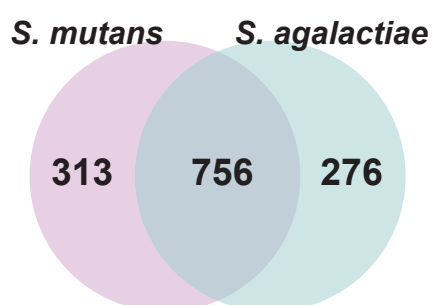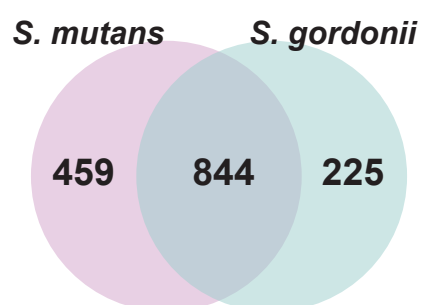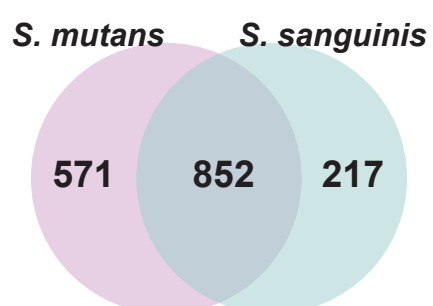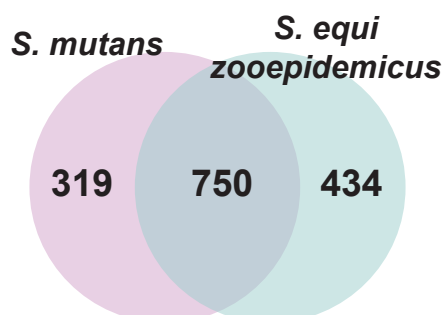

Supplement: Additional file 12 — Venn diagrams for the clustered gene groups in S. mutans species and eight other streptococcal species. All the predicted ORFs from the 32 streptococcal strains were clustered into groups based on a threshold of maximum E-value = 10-5 in the reciprocal BLATP analysis to compare the numbers of species-specific and -shared gene groups and those shared with S. mutans species. The number in the figure was the number of the gene groups, not of the ORFs (see Methods for details). [file 1471-2164-10-358-S12.pdf]

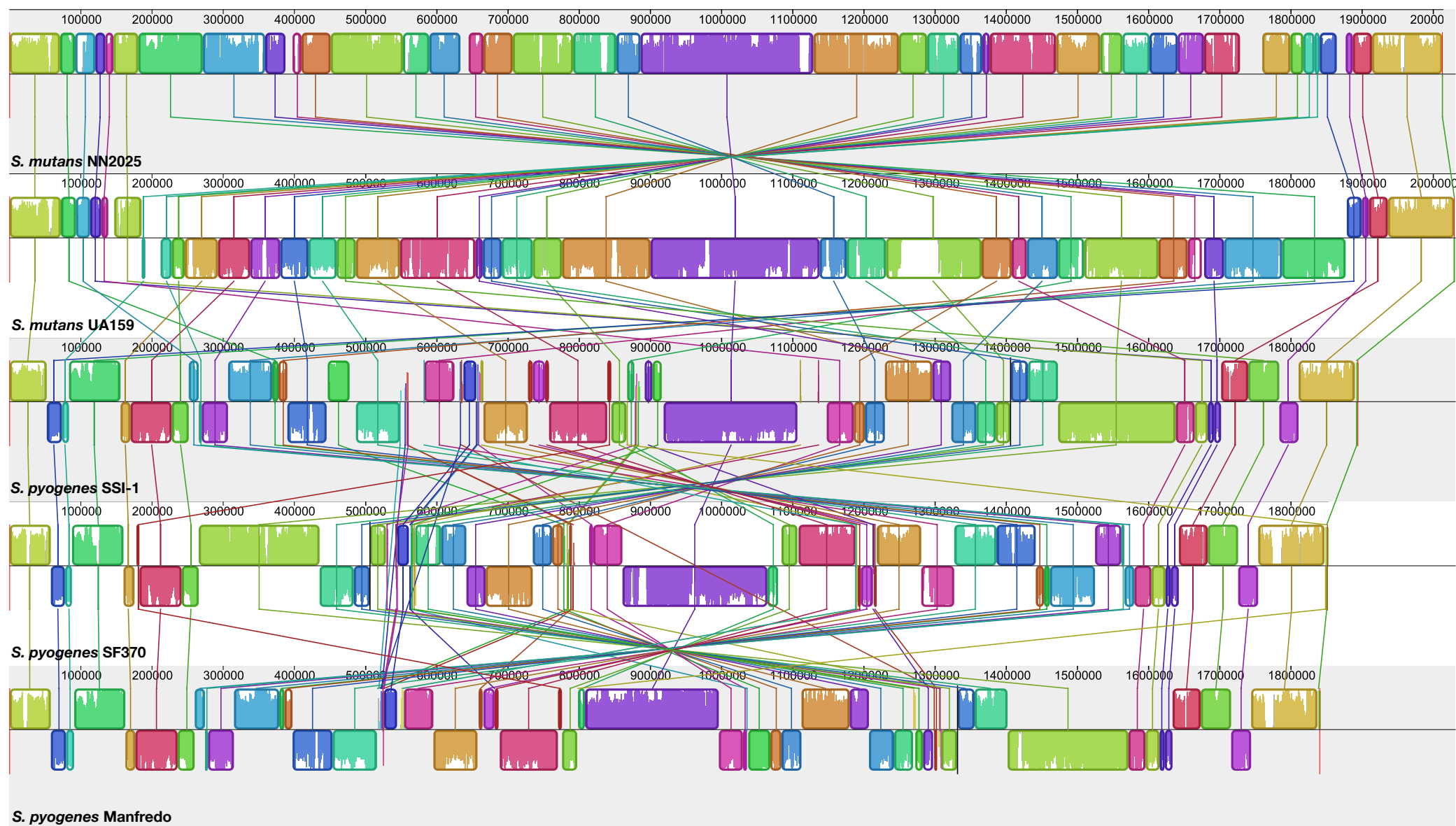

Supplement: Additional file 14 — Comparison of genomic shuffling between two strains of S. mutans (N2025 and UA159) and three strains of S. pyogenes (SSI-1, SF370 and Manfredo). A MAUVE representation of the total 64 local collinear blocks (LCBs) between chromosomal sequences of the S. mutans strains and S. pyogenes strains, at a minimum weight of 144. The S. mutans NN2025 DNA sequence given on the forward strand is the reference against which the sequence of the NN2205 was aligned and compared. LCBs placed under the vertical bars represent the reverse complement of the reference DNA sequence. LCBs placed under the vertical bars represent the reverse complement of the reference DNA sequence. The 64 connecting lines between genomes identify the locations of each orthologous LCB in the two genomes. Unmatched regions within an LCB indicate the presence of strain-specific sequence. Each sequential block represents a homologous backbone DNA sequence without rearrangements. [file 1471-2164-10-358-S14.pdf]

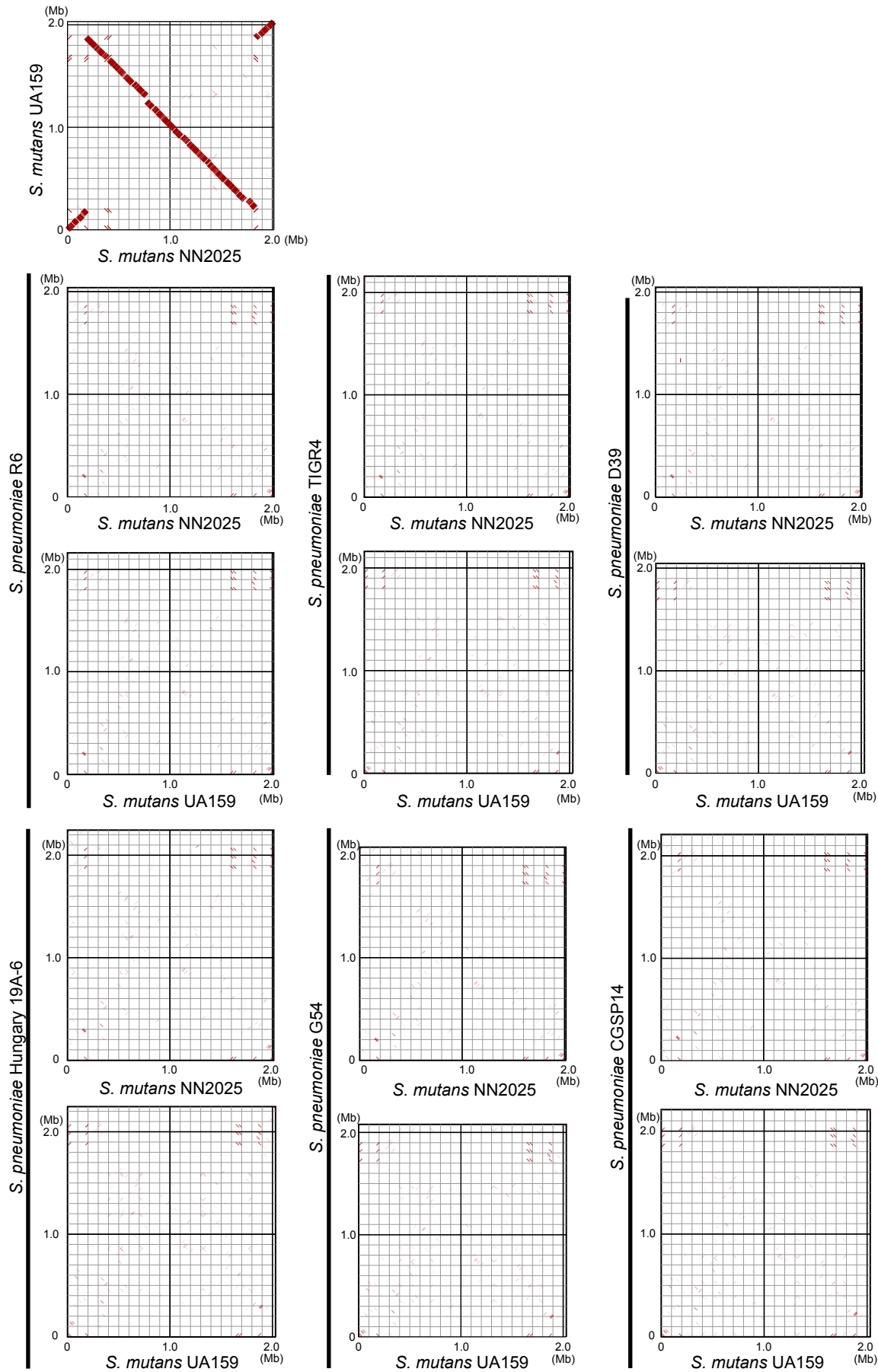

Supplement: Additional file 15 — Genome comparison of each S. mutans with six S. pneumoniae strains based on the chromosomal organization of the strain NN2025 or UA159. Dot plots of S. mutans NN2025 vs. six S. pneumoniae strains and of S. mutans UA159 vs the same set of S. pneumoniae strains are presented, as generated by PROmer of MUMmer software and visualized with GenomeMatcher software [116](see Methods). [file 1471-2164-10-358-S15.pdf]

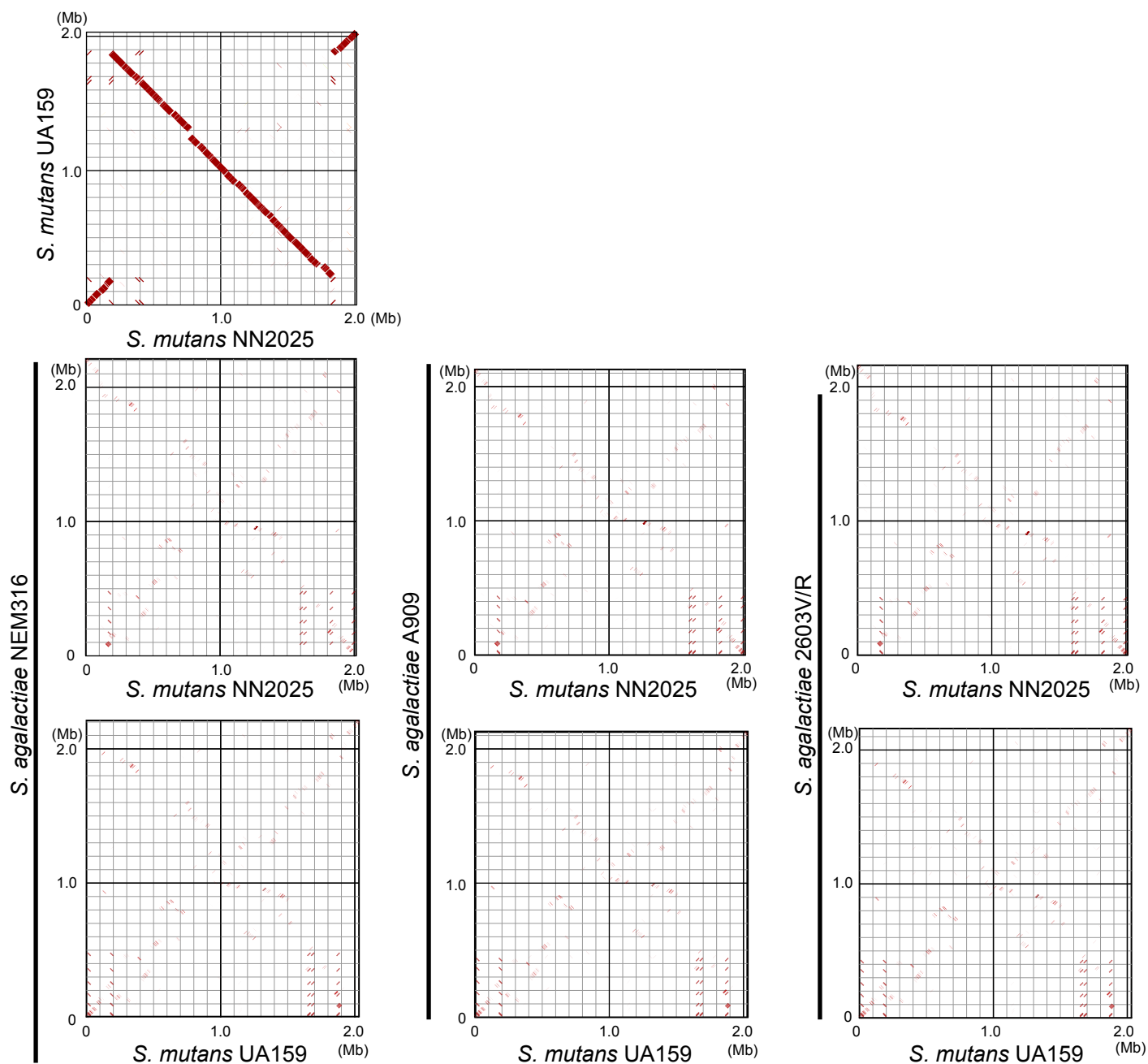

Supplement: Additional file 16 — Genome comparison of each S. mutans with three S. agalactiae strains based on the chromosomal organization of the strain NN2025 or UA159. Dot plots of S. mutans NN2025 vs. three S. agalactiae strains and of S. mutans UA159 vs. the same set of S. agalactiae strains are presented, as generated by PROmer of MUMmer software and were visualized with the GenomeMatcher software [116] (see Methods). [file 1471-2164-10-358-S16.pdf]

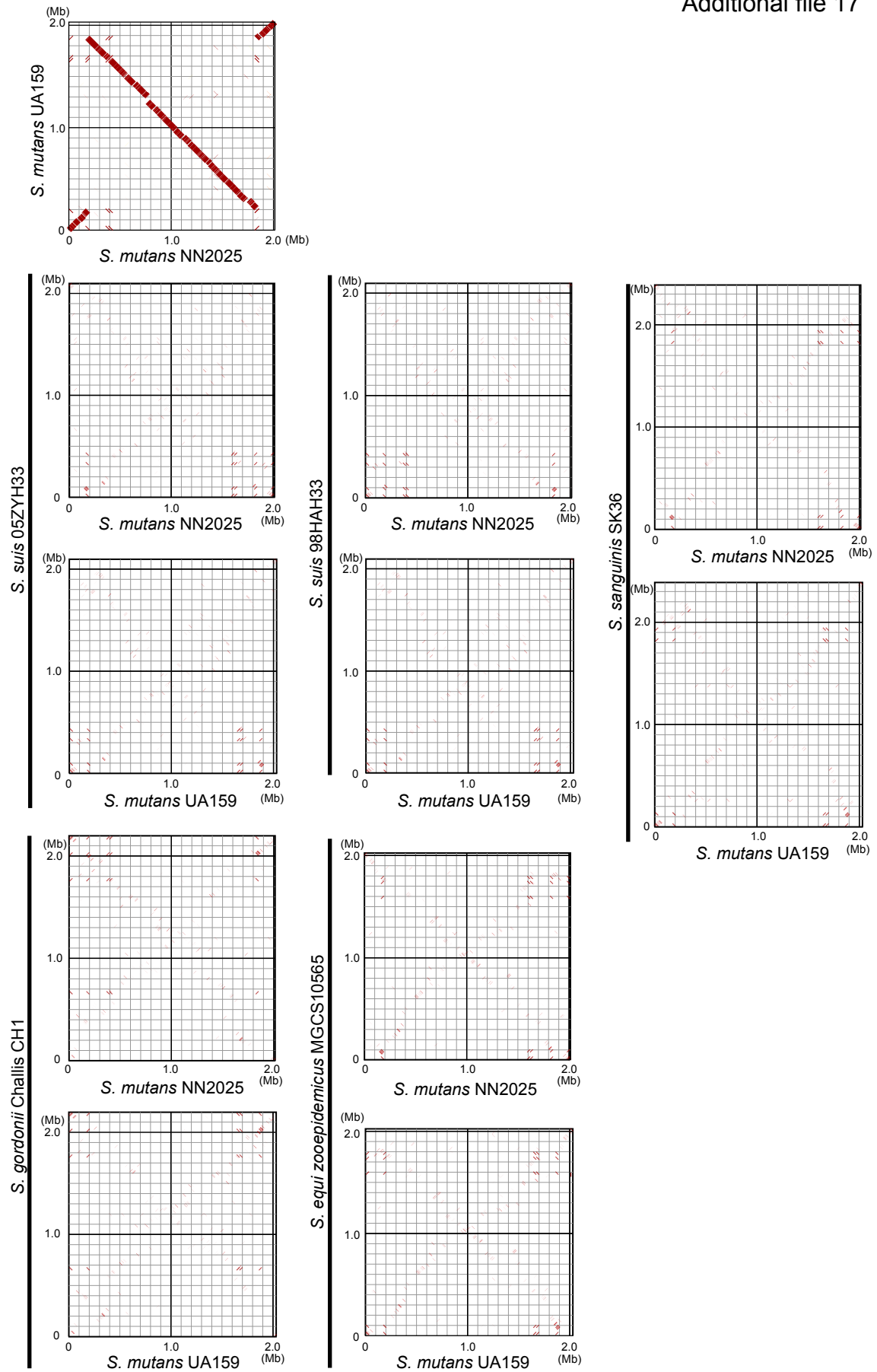

Supplement: Additional file 17 — Genome comparison of each S. mutans with three S. thermophilus strains based on the chromosomal organization of the strain NN2025 or UA159. Dot plots of S. mutans NN2025 vs three S. thermophilus strains and of S. mutans UA159 vs. the same set of S. thermophilus strains are presented, as generated by PROmer of MUMmer software and visualized with the GenomeMatcher software (see Methods). [file 1471-2164-10-358-S17.pdf]

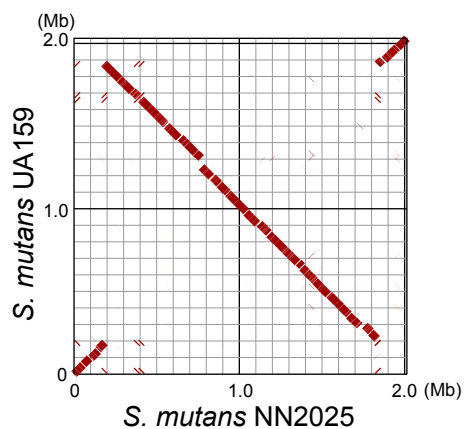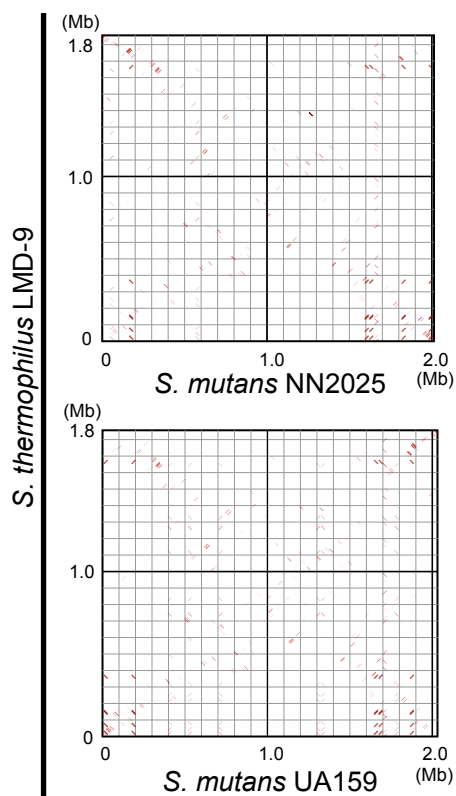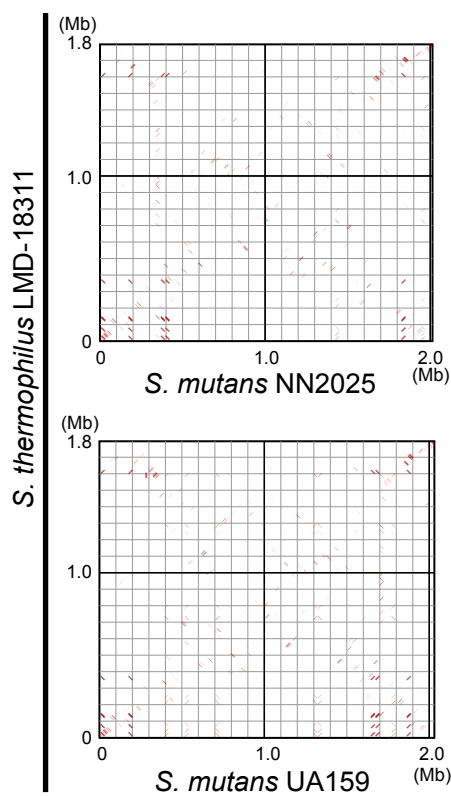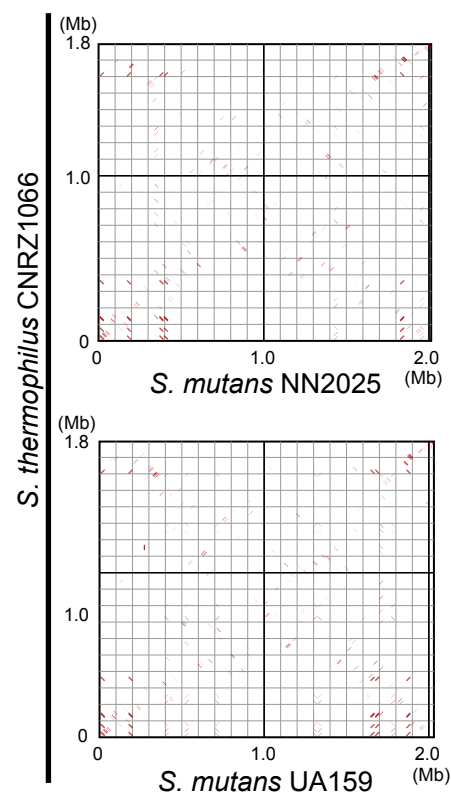

Supplement: Additional file 18 — Genome comparison of each S. mutans with two S. agalactiae strains, S. sanguinis SK36, S. gordonii Challis CH11, S. equi zooepidemicus MGCS10565 based on the chromosomal organization of the strain NN2025 or UA159. Dot plots of S. mutans NN2025 vs. two S. agalactiae strains, S. sanguinis SK36, S. gordonii Challis CH11, and S. equi zooepidemicus MGCS10565 and of S. mutans UA159 vs. the same set of streptococcal strains are presented, as generated by PROmer of MUMmer software and were visualized with the GenomeMatcher software (see Methods). [file 1471-2164-10-358-S18.pdf]

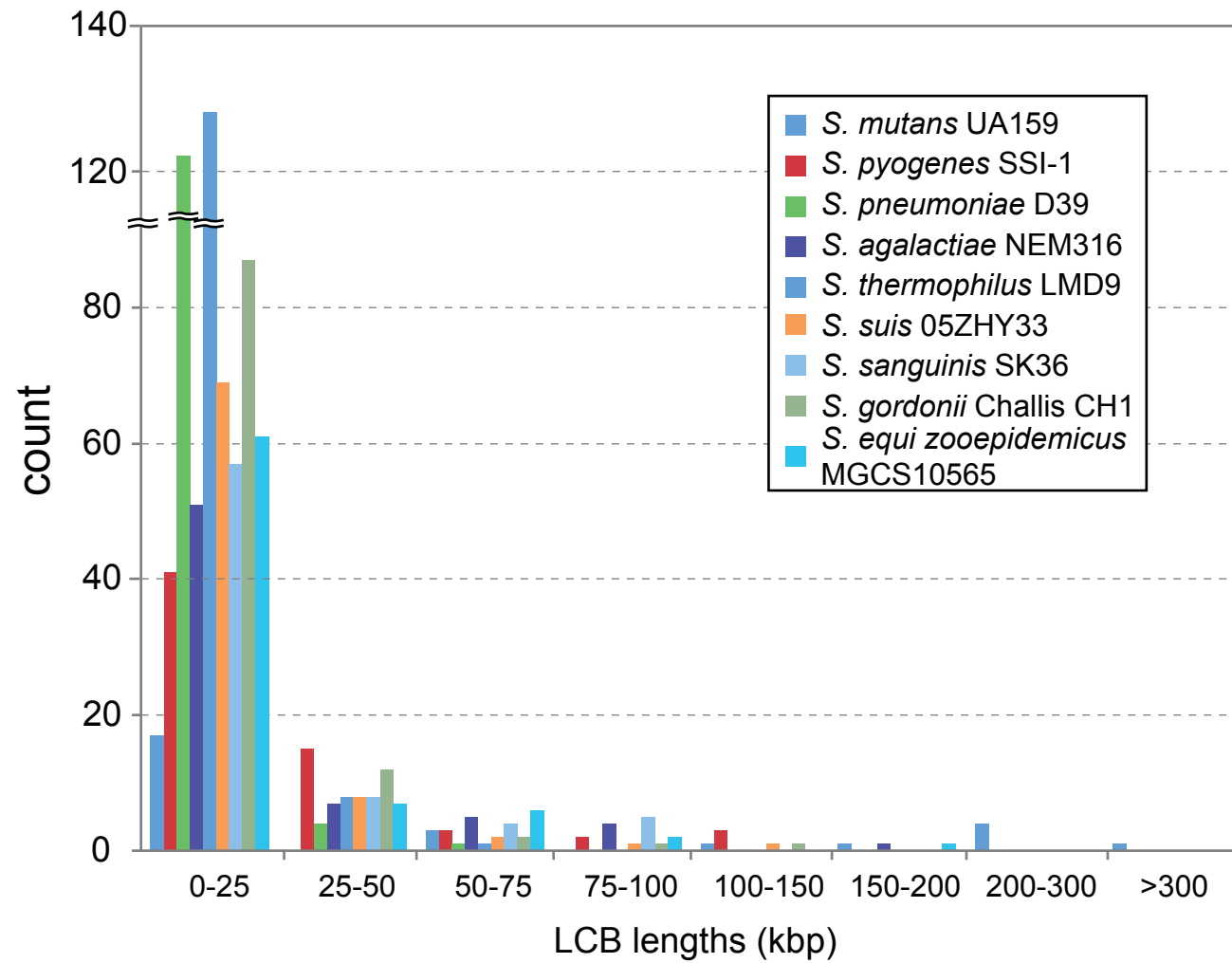

Supplement: Additional file 19 — Lengths of locally collinear blocks (LCBs) shared by the nine Streptococcal species. Block lengths are taken from the S. mutans NN2025 genome. Lengths of LCBs were determined using SPRING software. [file 1471-2164-10-358-S19.pdf]
