# Supplementary material for: Comparative genomic analyses of Streptococcus mutans provide insights into chromosomal shuffling and species-specific content
Source: BMC Genomics. 2009 Aug 5;10:358. doi: 10.1186/1471-2164-10-358 (PMC2907686; doi:10.1186/1471-2164-10-358)
Supplement: Additional file 8 — Characteristics of CRISPR loci found in S. mutans NN2025. The nomenclature, leader sequence, repeat sequence, number of repeats, and similarity were determined based on the method of Horvath et al. [64] with slight modifications (see Methods for details). [file 1471-2164-10-358-S8.pdf]

Additional file 8. CRISPR-associated ORF found in *S. mutans* NN2025

CRISPR-1 (Smut3) Ldbu1 family[1]

ORF	A.A. length	Description	Gene	E-value of NN2025 ORF	ORF in UA159	E-value of UA159 ORF
SmuNN2025.0603	937	CRISPR-associated helicase [*Lactobacillus casei* ATCC 334]	*cas3*	0	SMU.1764c	1.00E-10
SmuNN2025.0604	573	CRISPR-associated protein [*Lactobacillus casei* ATCC 334]	*cse1*	7.00E-78	-	-
SmuNN2025.0605	184	CRISPR-associated protein [*Lactobacillus casei* ATCC 334]	*cse2*	1.00E-17	-	-
SmuNN2025.0606	360	CRISPR-associated protein [*Lactobacillus delbrueckii* subsp. *bulgaricus* ATCC BAA-365]	*cas4*	6.00E-70	-	-
SmuNN2025.0607	239	CRISPR-associated protein [*Lactobacillus casei* ATCC 334]	*cas5*	1.00E-37	-	-
SmuNN2025.0608	199	CRISPR-associated protein [*Lactobacillus casei* ATCC 334]	*cas3*	2.00E-46	-	-
SmuNN2025.0609	314	CRISPR-associated protein [*Lactobacillus casei* ATCC 334]	*cas1*	0	-	-
SmuNN2025.0610	301	CRISPR-associated 3'-5' endonuclease [*Lactobacillus delbrueckii* subsp. *bulgaricus* ATCC BAA-365]	*cas2*	1.00E-60	-	-

Repeat seqeunce ATTTTACCCGCACGAGCGGGGGTGATCCT DR length : 29 (644279-645406), No. of spacers (18)

Leader sequence TAGCGGTTTTATTGGGATCTTTTAGT (28 bp)

CRISPR-2 (Smut1b) Sthe3 family[1]

ORF	A.A. length	Description	Gene	E-value of NN2025 ORF	ORF in UA159	E-value of UA159 ORF
SmuNN2025.0694	1346	Predicted CRISPR-associated nuclease, contains McrA/HNH-nuclease and RuvC-like nuclease domain [*Streptococcus agalactiae* A2603V/R]	*csn1*	0	SMU.1405c	0
SmuNN2025.0695	289	CRISPR-associated protein Cas1 [*Streptococcus agalactiae* A909]	*cas1*	0	SMU.1404c	0
SmuNN2025.0696	108	CRISPR-associated protein Cas2 [*Streptococcus agalactiae* A909]	*cas2*	2.00E-46	SMU.1403c	2.00E-57
SmuNN2025.0697	221	CRISPR-associated protein, SAG0897 family [*Streptococcus agalactiae* COH1]	*csn2*	2.00E-81	SMU.1402c	7.00E-102

Repeat seqeunce GTTTTAGAGCTGTGTTGTTTCGAATGGTTCCAAAAC DR length : 36 (644279-645406), No. of spacers (69)

Leader sequence TAAAACAAGAAAAGCGCTAGAAAGATTGATTTCTAGCGCTTTTTTAGGTATAATATAATTAATTAAAATCT TTTAAAAAGGAATTATTTGAAGCTGAATTCTAGCTGAGATGAATGGCGCGATTACGAAATGTCGTGACGA AAATTGGTCCACGAG (156 bp)

1: Horvath et al [63].
