# Supplementary material for: Comparative genomic analyses of Streptococcus mutans provide insights into chromosomal shuffling and species-specific content
Source: BMC Genomics. 2009 Aug 5;10:358. doi: 10.1186/1471-2164-10-358 (PMC2907686; doi:10.1186/1471-2164-10-358)
Supplement: Additional file 9 — Sequence similarities of the existing CRISPR spacers in S. mutans NN2025. The spacer similarities were determined by BLASTN against viruses including bacteriophage or bacteria databases (see Methods). No description in "origin" or "BLAST E-value" indicates that no similarity was found in the database. [file 1471-2164-10-358-S9.pdf]

Additional file 9. Sequence similarities to the acquired CRISPR spacers in S. mutans NN2025.

Spacer No.	Spacer sequence	origin	Blast E-value	Alignment length	%identity	Match with phage/ prophage	Match with plasmid	Match with chromosome
CRISPR-1 (Smut3)								
1	GGGTCTGACACCCAGTCACCCAAAGCTTTGAG	*Streptococcus* phage M102 ORF DNA	0.001	30	93	yes		
2	AGTTCAACCGCTTATCTTTACAGCAAAGAAAA	-						
3	GCTTGGAAAGATTGACCACACAGGGTCAGGCG	-						
4	ACTTGACAGCGCAGTGCGCGCTGTTGAATAGC	-						
5	AGCTCCAACAGCTACAACAGCTACAACAGCTA	*Shigella dysenteriae* Sd197	5.00E-06	31	96			yes
6	GGTAAGGAGGTGCGATTATATGCAAGAGTTAG	*Streptococcus* phage M102 ORF DNA	0.90	18	100	yes		
7	CATGCGCCAGTATATTATCAGGTCGTGGAATA	*Streptococcus* phage M102 ORF DNA	0.001	32	93	yes		
8	AAAATATACTTCTTTTGTGAAAGGGTTGAGAT	*Acinetobacter baumannii* ATCC 17978	0.015	21	100			yes
9	TTGATTTTCATTATCGCGGTCTGATGGCTTAAA	-						
10	CGGCTGACAAGCCTAAAATTGTTAATGTTTTC	-						
11	AACTCGCTAGCTTTTTTAGATACATAGCTCTT	-						
12	TACTGGAGACTCCGCTTATCCCTTTTTTGATG	-						
13	AGTTTTCGTGCGATTGCTTCCAAAATTTGAAA	*Nitrosomonas eutropha* C91	0.004	22	100			yes
14	ATATACGACAGTCTGAAAGAAGGCTACCGCGG	-						
15	GACCTCAGTTGCTATCCTCTTTTTTATTGTTG	-						
16	GACGGCAACGATAGCCCTGAGACTGACAATCG	*Streptococcus* phage M102 ORF DNA	3.0	17	100	yes		
17	GTTTTCCTACCTCTTACCAAGGTTTTTTCTAA	-						
18	GCCGTTGGGTCAATGCCGTTAGCTTTTAAAGA	*Streptococcus* phage M102 ORF DNA	0.018	25	96	yes		
CRISPR-2 (Smut2b)								
1	CAAAAGGGTTTCTAATCCCATTCGAAAGCG	-						
2	GATTGTGCCCGCTAGTAAACCGCCTCGCGC	*Streptococcus* phage M102 ORF DNA	0.003	30	93	yes		
3	CCAAGCTATGCAGTCACGCCTGACCCTGTG	*Streptococcus* phage M102 ORF DNA	0.65	24	92	yes		
4	TAGAGTTGAAAAAAACACTACAGGCGAAAA	-						
5	TAATACTTCCTCAATTATAAATTCAATTCT	-						
6	GATTGTATCAGTAATCGAACTTCTGCTTAT	*Streptococcus* phage M102 ORF DNA	0.94	27	92	yes		
7	GTTGCTTATGCAGATGATAACACAGGCGCA	-						
8	TGGTCCAAAGTGCAGAGCCAAAGAAAAACA	*Streptococcus* phage M102 ORF DNA	6.00E-05	26	100	yes		
9	ATTGTCAATCGCCGTTCTGCGCTTGCGACG	*Streptococcus* phage M102 ORF DNA	3.7	27	90	yes		
10	CTCTAGCGCTTATTTCGAGGACAGCGGAGC	-						
11	TCATTGCTCTTGACATCAATAACTTTTTCA	-						
12	GAGCAAAATCAACTATCTCAAAGCTTAGTT	-						
13	TGGCAATTTACCAGCACAGCAATTGCTGGC	-						
14	TTCGCGGTCTACTTTTTTTGATATGCTAAT	-						
15	AAAGAATAAGTTCTCTCACAATGTTTGAAA	-						
16	CAGGTTTCATCTTAGGAAACGTATTGATGA	-						
17	GCTTGAATATAATTGTGTATCCGCCAATGA	*Streptococcus* phage M102 ORF DNA	0.015	25	96	yes		
18	ACCTTGTGGTCCGTCTTCTCCGCCACTTTC	-						
19	CAAACGGCGGTTTGACTGACTCAGGAATTT	-						
20	GATTAAAAACATAGATGAAAACGGCTATTT	-						
21	ATCCACAGGCACAGAATTAGTGTTAATCCA	-						
22	TTGACCGTGATAAAGTGTTCAACCACACGT	-						
23	AAAAAGAAACGCCTTTTGATTTGACCAATC	*Streptococcus* phage M102 ORF DNA	0.015	22	100	yes		
24	CAATGCTAGTTAAGCTCTTGATGCTTTGAG	-						
25	CCACTCTTGCTCCTGATTGGTCAAATCAAA	-						
26	GTTCCATTGAACGCATTGATGGGAACAACA	-						
27	CGTTACTGGTGTTCACGGCGACAGCATGGA	-						
28	ATGAAAAGCCTCGTTTCTAAGGCTTTTGGT	-						
29	AGTTATTAATATCTATGACAGTCTCAAAGA	*Streptococcus* phage M102 ORF DNA	0.015	22	100	yes		
30	CGGATATAGCCTAGCATATAATCGCGTGTG	-						
31	TTCAATCGGTGATGATATTGTCTGCTATCA	-						
32	AATGTATGCACCGCGTTTTTTAACAAAATA	*Streptococcus* phage M102 ORF DNA	3.7	18	100	yes		
33	GTTTGCTTTTGTTTTTGCCTTGTGTGTTTC	-						
34	ATAAAGACGAATATAAAGACCCGAGCACTT	-						
35	TAGACACGGTTGCTAAAACAAAGAAGGTTA	-						
36	TAATATTATTGAGCAAAAATCCTTTAACTC	-						
37	TTCTGGCTGTCTTTCAGAGTGATAAGCGCA	*Streptococcus* phage M102 ORF DNA	0.015	25	96	yes		
38	GTATCAATACAATCTTAGACGCTCGCACTG	-						
39	CGCCGGTTACAACCGGTTGAACGCCTGCGT	-						
40	TGCAAGTTATCTTGCTATGTGGACGAATTG	*Streptococcus* phage M102 ORF DNA	0.015	28	93	yes		
41	TGACGGAAACAGAGACGGAACGGCAACTGT	*Streptococcus* phage M102 ORF DNA	0.94					
42	GCACTACAAGACGGCTATTGCTGATGAAGT	-						
43	GCAATTTAGTTTTATTCCGTGGGAGCAGCA	-						
44	CGATAAAATTTTGCTAACCCGGAAAGCGGTT	-						
45	AATTGTTACTCCCAACTTCAGCATTGACCA	-						
46	CTCTGCGCTTTGCACGAGCACAGCGATATC	-						
47	TGGCCATGACGATGACGGGACTAAGACTGT	-						
48	AGAGTATAGCCAGTGTTTTCAAGGCCTTTA	*Streptococcus* phage M102 ORF DNA	0.24	20	100	yes		
49	CGCAACAATGACTATTAATATCAACGGTGG	*Streptococcus* phage M102 ORF DNA	0.004		yes			
50	ATCAAATTTCGCTTCTGGAGCAATCTCAAC	-						
51	AAGAACTCTTAGAAAATATGGTCCACTCAT	-						
52	CAGTATACGCCATATTCCATCAGGATGACT	-						
53	CGATAAAGCTACTGTAGCAATTAAAGGCTA	-						
54	TTTCTGTTTTAGCTGAGTTAAGTGCCTTCT	-						
55	TTAAAAAGCTGACAAAAGACAGCAATGAGA	-						
56	AATCGCTTCTTTGCTAACCACAATTTGTGC	-						
57	TTAAAAAGCTGACAAAAGACAGCAATGAGA	-						
58	TATATGTGATGGCAGTTGAAAACATACGTA	-						
59	TTGCAATTGAGGGCAAACGTAGTGAATTGC	-						
60	AAATGCTCTTGAAGAACCTGATAGATGACA	*Streptococcus* phage M102 ORF DNA	8.00E-04	27	96	yes		
61	TCAGATAAAGATTTTTTTGGAAAGTACAAT	-						
62	TATTAAATTACTTGTAATTACAAAGCCTAA	-						
63	ATTAGAGAGCAGATGCAATCACCCGTTATG	-						
64	TAGAGCTCTTAGTTTTTTACGCGTTGGGCC	-						
65	CAAATTTGCCGGCGGGGTCGGTAAAACGGTC	-						
66	TGCAAAAGATGGCCTCGAGCAATTATCGCA	*Streptococcus* phage M102 ORF DNA	2.00E-07	26	96	yes		
67	GAAAAGGAAAATCTTACTTAGCTCATGAGT	-						
68	CTGACCAAGAGAATTAATTCTAAAGATAAA	-						
69	CTGACCAAGAGAATTAATTCTAAAGATAAA	-						

1: BLAST database minus (-) indicates that a spacer sequ
