# Supplementary material for: Comparative genomic analyses of Streptococcus mutans provide insights into chromosomal shuffling and species-specific content
Source: BMC Genomics. 2009 Aug 5;10:358. doi: 10.1186/1471-2164-10-358 (PMC2907686; doi:10.1186/1471-2164-10-358)
Supplement: Additional file 10 — Distribution of CRISPR-2 (Smut2b; Sthe3 family)-associated repeat sequences in genus Streptococcus. Repeat sequences in the CRISPR-2 homologous region (Sthe3 family) were found in 19 of 32 streptococcal genomes (see Methods; for NN2025, see additional file 8). Similarities of the direct repeat sequence of the strain NN2025 (Sthe3 family) were examined by BLASTN against each genome as a target database. The number of repeats was determined based on Horvath et al. [64] (see Methods). [file 1471-2164-10-358-S10.pdf]

Additional file 10. CRISPR spacers sequences in *S. mutans* strains.

Strain	Spacer sequence	Subject definition	Blast E-value	Alignment length	%identity	Match with phage/ prophage	Match with plasmid	Match with chromosome
Strain UA159: DR GTTTTGGAACCATTCGAAACAACACAGCTCTAAAAC (36 bp)								
1	TGTGGCAAGTCATGTCCGAATACATAGGCA							
2	GTTTGTCTATTGTCTAATTGAAAATATTTA							
3	CTAACTATGATGACACAACAGCTTTTAGCG	*Streptococcus* phage M102	2.00E-07	30	100	yes		
4	AGAGCACTAACTGCGCTAGCTGGTTCAATC	*Streptococcus* phage M102	2.00E-07	30	100	yes		
5	TCACCATATTAATTAATGGCGTTTCCTTTT							
Strain MT8148: DR GTTTTGGAACCATTCGAAACAACACAGCTCTAAAACT (37 bp)								
1	AAGTGCACTTAAAAATGCTTTGAATGCT							
2	CTTGCCAGCTGGGGCAGAAGTTTTGAACG							
3	GGCCCCTTAGTTGAATATGTAATTGCATGGT							
Strain LJ26: DR GTTTTAGAGCTGTGTTGTTTCGAATGGTTCCAAAAC (36 bp)								
1	AACCGTAAGCTTTGATTGCTGTACCAATGC							
2	GCCTGTGTAGACTGCAACAGTACCAGGTTT							
3	CCAAATCCTGCATTTTGTTGTTTAGTCTGC							
4	GTCAAGTACCAACAAACAATGAATGCTACA	*Streptococcus thermophilus* bacteriophage Sfi21	0.003	25	96	yes		
5	ATTTGCTAAACAGCAAACAGCAGATTATAA	*Streptococcus* phage M102	0.003	28	93	yes		
6	GTGTTTCTGGTCTTAGATACTTACTCATGC	*Streptococcus* phage M102	0.042	26	92	yes		
7	TGCAAGAAATTTTGGAATAGGAGGTAAACA							
8	GTTTCGATAGCGTCTGCCATATGTTCACCC							
9	GCTTCAAAGTCTTTTTGGATGCGGTTTAGT							
10	TCAAGATAAGTGGAAGCTGATGAAATAGGT							
11	TGTACGATATGGGCGGAGAATGACTGGGTT							
12	CTCACGTTTGGCTAATTTGGGTGTTAATCC							
13	TAACTTAGTAGCCCTGCGTCCTGCGATTTC							
Strain SA13: DR GTTTTAGAGCTGTGTTGTTTCGAATGGTTCCAAAAC (36 bp)								
1	AAAATTGCAAACTTATACTAAACTTGAAGA							
2	TGAATGATTAATGAATTAAGACAGAGGAAA							
3	TTAAAGAACTGACAGAAGACAGTGATAAGA	*Streptococcus* phage M102	0.003	28	93	yes		
4	TTTGCTTTTGTCTTTGCCTTGTGTGTTTCA							
5	TATGCTACATTTCAAAATGTTGACTTTGAA							
6	TGTTAAACTTTGCTTCTGGAGCAATTTCTAC							
7	TTATCTATTTTGACCAAGACACGAAAACAC	*Streptococcus* phage M102	2.6	26	89	yes		
8	GACAATCTTTAGTGAATGTTCTTTGAATTC							
9	TAATTTATACATTGCAGCCATGGAGAAACT							
10	AGCAAGGATTTTACTTACCCTAAAGTCCGT							
11	TTAAATTGCTATCAGCCCCTGCGTTTTGCT							
12	CGAGACAATGAGGCAAGTATGCTTTTAGGC							
13	GTTAGTCGAGACCTCGAAGCTTGACATCAA							
14	GGAGCTTTACTTGCCAGATTAAAGACGGCT	*Streptococcus* phage M102	0.17	22	95	yes		
15	TACAACGTAACTTTTGATGACTCTAGATTA							
16	AACTTCTTCAGCTGATAGCTTTTCTGCGTT							
17	GCTTCTGCAAAGCCCAACCATCTCGCCGAA							
18	GCGATTGACTGCAGTTATTAATACTAATGC	*Streptococcus* phage M102	0.66	27	90	yes		
19	ATGCTCATGATATTATCTTCGAATCGCCAA							
20	CAATCGCGCCGCGGATTAGATTGAGCCCGT							
21	GACGCGCTTGACTCTTAATACTAATATTAA							
22	ACAGTTCAGCTTCATTCCTTGGGAGCAACA							
23	AGTATATAATATAGTCAGAGAAAAGGAAAG							
Strain SA15: DR GTTTTAGAGCTGTGTTGTTTCGAATGGTTCCAAAAC (36 bp)								
1	GAATTACGGTGATGGCAGTAACTTGATTAC							
2	TAGAGCAACATCTAATCAATGGTCAATGAA							
3	CGTGAAGTTAAGCGTTCCGCTTGCGACGAA	*Streptococcus* phage M102	6.00E-05	26	100	yes		
4	AGGTACTGAAAAACCAAGTGAATGGTCTGC							
5	ACGTGTTGATGTATCAGACATATTTTCCAG							
6	AGAAGCAGAGCAGACACCGTGCGCATCTGC							
7	CTTTAGCGATAATATCGGCTGGTACTGGCT	*Streptococcus* phage M102	0.66	27	90	yes		
8	CTGGTCATGATAGATATTTTCTAGCGTAAA	*Streptococcus* phage M102	0.003	28	93	yes		
9	TCCACTTGTCGCAATTCAGCTTTATCCCCT	*Streptococcus* phage M102	0.003	28	93	yes		
10	TGCTGTTAGTGCTCTCGGCGTAATCACTCC							
11	GTCATAGATAATAAGTGTGATGAGTGGCT							
12	TAATTAAGGGCATAATTTGATGAAATATAT							
13	GCCTTATGCAAAGGTTACTAGTCATGAGTT	*Streptococcus* phage M102	0.17	22	95	yes		
14	GCAGAAGAGAGGCGTATGCAGCCTTGTAGA							
15	CGTATTTAAAGCATATTTGTTCTTTGCAAG							
16	TTTCGGACTGGTGAGATAGCTGGACTGACT							
Strain NN2007: DR GTTTTAGAGCTGTGTTGTTTCGAATGGTTCCAAAAC (36 bp)								
1	ATTCACAAAGGAAGTTTACTTCATCAAATA							
2	TTCGAATATTCTCAAGCAATTTTGGGATAT							
3	AAGAATGGTTTTGACTATGAACAACTGTTG							
4	ACAGTTATCCAAAATCAAAGTCATTCTTTG							
5	GCCTGCTCCAGTGTCCTCAAAATAAGCGCT							
6	ATAGCCTTTATCCATTAAAAGAATCGGTAA							
7	TTGCATAAGCATATTTGCTTTATCCTCAGC							
8	AGTAGAAAGAAAAAAAATGACAGAAGAACA							
9	GATACATTGCATCAATAATTGCATCAACAG							
10	TGTGGCACAAAGCTTTGCCCGTTTGGCAAG							
11	AGTAGAAAGAAAAAAAATGACAGAAGAACA							
12	CATTGTAGACCAATGCTTTTCTCCTTGTCC							
13	AAGCGCTCTGAGGTTGTCTTGTTAGAAAAT							
14	CATTAGTTTTAAAATCACCATCAGCCGCTT							
15	CATCAATAAGCCTCTCATAATGAGGACTAT							
16	CGTTTGGGGACGATTCGAGGAGTCCCTCAA							
17	AAAGATGTTATGTGAAAAAACTCAAAGCAC							
18	TACTTAGTCAAAAAGCGTGGCGCATACATT							
19	TCTCGTTTACTTTTAGCCTCTATGGAGCCT							
20	ACTTCAAACAGGATTGAGAAACTCGCTGGA							
Strain NN2138: DR GTTTTAGAGCTGTGTTGTTTCGAATGGTTCCAAAAC (36 bp)								
1	GAGCGACCATGGCTAAACCGTTAGTATACA	*Streptococcus* phage M102	2.6	20	95	yes		
2	AGTGAAGAAGAAATTAACGACCCTTCGACA							
3	AAGTTTATCTATCAGCCATTCGTGCTTTTC							
4	AGTTAAATGCCACTTGTGGAGCCCCCGGAT							
5	GCGTCAGTCAAAGCTATACCGTCTTTAACC							
6	CCATTCTTCTCCTTTCTATCCTAACGTTGT							
7	TTTAGTAGTAAGTCTAAGCGGACCATCTAA							
8	AGCGCTACACGTAACAATCAAATTGTGCGC							
9	AAGCTTAACGGAACGCTTTATGTCAATGAC							
10	CACTAATTCAGGCGTTCAGCCTGTAATTAC							
11	TCAGATTGCATGCCTCGCGAGTCAGTAACA							
12	GCAAGTTGAGTTTAGATTTGTTTACATAAT							
13	CTTTAATGCGTTTTGCTTTCTTATCGTCTA	*Streptococcus* phage M102	0.85	27	90	yes		
14	CGTGACTGTCTTAGCTTTTCAGTTGCTGAT	*Streptococcus* phage M102	9.00E-04	26	96	yes		
15	GCTTTTCAAATTTGGGGAAGCGATTATGAC	*Streptococcus* phage M102	3.4	26	89	yes		
16	CGCTCGTTCTCTGTACTCGATGCTGATTTC	*Streptococcus* phage M102	0.003	25	96	yes		
17	TCAAAGCTGACGAAGATGCGGTTAAGGCGC							
18	TGCAAAAGGATTTTTAATTCCATTCGAAAG							
19	ATACTAAGCAATTGAAAAGAGGAGCAATCA	*Streptococcus* phage M102	6.00E-06	30	100	yes		
20	ATAGTGTATGCGATGAGTTTTGGAGAAGCG	*Streptococcus* phage M102	0.85	24	92	yes		
21	AGTTGATGTACAAAGAAAGTTTGAAAGCGC							
22	TATTTCACATCTGTTTTCGTCAATGCACTC							
23	TGCTCGTTCTCTATACTCAATACTGATTTC							
24	TCACTACAACGCTGGTCAACTATATCCGAA							
