# Supplementary material for: Comparative genomic analyses of Streptococcus mutans provide insights into chromosomal shuffling and species-specific content
Source: BMC Genomics. 2009 Aug 5;10:358. doi: 10.1186/1471-2164-10-358 (PMC2907686; doi:10.1186/1471-2164-10-358)
Supplement: Additional file 11 — Distribution of CRISPR-2 (Smut2b)-associated repeat sequences in genus Streptococcus. Repeat sequences in the CRISPR-2 homologous region (Smut2b; Sthe3 family) found in streptococcal genomes except S. mutans NN2025 (15/31 strains; see Methods). Similarities were examined by BLASTN against each genome as a target database. The number of repeats was determined as described by Horvath et al. [64] (see Methods). [file 1471-2164-10-358-S11.pdf]

Additional file 11. Distribution of CRISPR-2 (Smut1b) direct repeat sequences in genus streptococci

Accession No.	Origin	CRISPR locus name	No. repeat	Repeat size (bp)	Spacer size (min-max) (bp)	cas genes	Typical repeat sequence
AP010655	*Streptococcus mutans* NN2025	Smut1b	70	36	30 (30-31)	yes	GTTTTAGAGCTGTGTTGTTTCGAATGGTTCCAAAAC
CP000419.1	*Streptococcus thermophilus* LMD-9	Sthe3c	8	36	30 (30-32)	yes	GTTTAGAGCTGTGTTGTTTCGAATGGTTCCAAAAC
AL766848.1	*Streptococcus agalactiae* NEM316	Saga1h	15	36	30	yes	GTTTTAGAGCTGTGCTGTTTCGAATGGTTCAAAAC
AE014133.1	*Streptococcus mutans* UA159	Smut1	7	36	30	yes	GTTTTAGAGCTGTGTTGTTTCGAATGGTTCCAAAAC
CP000114.1	*Streptococcus agalactiae* A909	Saga1d	16	36	30	yes	GTTTTAGAGCTGTGCTGTTTCGAATGGTTCCAAAAC
AE014233.1	*Streptococcus agalactiae* 2603V/R	Saga1c	26	36	30 (29-31)	yes	GTTTTAGAGCTGTGCTGTTTCGAATGGTTCCAAAAC
CP000829.1	*Streptococcus pyogenes* M49 NZ131	Spyo1a	5	36	30 (30-31)	yes	GTTTTAGAGCTATGCTGTTTTGAATGGTCCCAAAAC
CP001129.1	*Streptococcus equi* subsp. *zooepidemicus* MGCS10565	Sequ2	18	36	30	yes	GTTTTGGAACCATTCAATACAGCATAACTCTAAAAC
AE004092.1	*Streptococcus pyogenes* M1 GAS	Spyo1h	7	36	30	yes	GTTTTAGAGCTATGCTGTTTTGAATGGTCCCAAAAC
CP000261.1	*Streptococcus pyogenes* MGAS2096	Spyo1d	3	36	30 (30-31)	yes	GTTTTAGAGCTATGCTGTTTTGAATGGTCCCAAAAC
CP000260.1	*Streptococcus pyogenes* MGAS10270	Spyo1b	3	36	30	yes	GTTTTAGAGCTATGCTGTTTTGAATGGTCCCAAAAC
CP000259.1	*Streptococcus pyogenes* MGAS9429	Spyo1g	3	36	30	yes	GTTTTAGAGCTATGCTGTTTTGAATGGTCCCAAAAC
CP000017.1	*Streptococcus pyogenes* MGAS5005	Spyo1e	4	36	30	yes	GTTTTAGAGCTATGCTGTTTTGAATGGTCCCAAAAC
CP000056.1	*Streptococcus pyogenes* MGAS6180	Spyo1f	5	36	30 (30-31)	yes	GTTTTAGAGCTATGCTGTTTTGAATGGTCCCAAAAC
