# Supplementary material for: Comparative genomic analyses of Streptococcus mutans provide insights into chromosomal shuffling and species-specific content
Source: BMC Genomics. 2009 Aug 5;10:358. doi: 10.1186/1471-2164-10-358 (PMC2907686; doi:10.1186/1471-2164-10-358)
Supplement: Additional file 13 — Specific gene groups shared in oral streptococci. All the predicted ORFs from the 32 streptococcal strains were clustered into groups based on a threshold of maximum E-value = 10-5 in the reciprocal BLATP analysis to extract the ORF(s) of gene groups specific for oral streptococci (S. mutans, S. sanguinis, and S. gordonii). The predicted function is assigned based on the COG classification (see Methods for details). [file 1471-2164-10-358-S13.pdf]

Additional file 13. Specific gene groups shared in oral streptococci.

ORF No. in each strains				Predicted function of gene products
S. mutans NN2025	*S. mutans* UA159	*S. sanguinis* SK36	*S. gordonii*	
NN2025.0133	SMU.160	SSA_0367	SGO_0271	Predicted Zn-dependent protease
NN2025.1728	SMU.227c	SSA_0288	SGO_1785	Putative protein-S-isoprenylcysteine_methyltransferase
NN2025.1804	SMU.2061	SSA_2344	SGO_0279	Protease subunit of ATP-dependent Clp proteases
NN2025.0199	SMU.1956c	SSA_0224	SGO_1889	Mismatch repair ATPase (MutS family)
NN2025.0034/1367/ 1674/1773	SMU.39/622c/ 293	SSA_0714	SGO_1603	Cation transport ATPase Predicted membrane protein Ribosomal protein L31E ABC-type phosphate transport system permease component
NN2025.0434/0638/ 0602	SMU.1471c/ 1683c/1765c	SSA_0667	SGO_0686	Predicted transcriptional regulator Predicted transcriptional regulator Predicted transcriptional regulator
NN2025.0473	SMU.1643c	SSA_1705	SGO_0380	Uncharacterized protein required for cytochrome oxidase assembly
NN2025.0519	SMU.1593c	SSA_1011	SGO_0935	CDP-diglyceride synthetase
NN2025.0556	SMU.1552c	SSA_0368	SGO_0272	ABC-type sugar transport system periplasmic component
NN2025.0601	SMU.1502c	SSA_0599/0394	SGO_0297	Predicted membrane protein
NN2025.0639	SMU.1470c	SSA_0668	SGO_0687	Uncharacterized conserved protein
NN2025.0663	SMU.1442c	SSA_0004	SGO_0004	Uncharacterized conserved protein
NN2025.0732	SMU.1335c	SSA_0992	SGO_0895	Uncharacterized conserved protein
NN2025.0784	SMU.1271	SSA_1447	SGO_1409	ATP phosphoribosyltransferase
NN2025.0785	SMU.1270	SSA_1446	SGO_1408	Histidinol dehydrogenase
NN2025.0787	SMU.1268	SSA_1445	SGO_1407	Imidazoleglycerol-phosphate dehydratase
NN2025.0790	SMU.1265	SSA_1443	SGO_1405	Phosphoribosylformimino-5-aminoimidazole carboxamide_ribonucleotide (ProFAR) isomerase Imidazoleglycerol-phosphate synthase
NN2025.0791	SMU.1264	SSA_1442	SGO_1404	Phosphoribosylformimino-5-aminoimidazole carboxamide_ribonucleotide (ProFAR) isomerase Imidazoleglycerol-phosphate synthase
NN2025.0792	SMU.1263	SSA_1441	SGO_1403	Phosphoribosyl-ATP pyrophosphohydrolase
NN2025.0793	SMU.1261c	SSA_1440	SGO_1402	Phosphoribosyl-ATP pyrophosphohydrolase
NN2025.0997	SMU.1036	SSA_0441	SGO_0305	Predicted symporter
NN2025.1031	SMU.992	SSA_1671	SGO_1493	RecB family exonuclease
NN2025.1218	SMU.789	SSA_0253	SGO_1819	Predicted metal-dependent membrane protease
NN2025.1443	SMU.527	SSA_2021	SGO_0387	Uncharacterized conserved protein related to dihydrodipicolinate reductase
NN2025.1522	SMU.442	SSA_0254	SGO_1818	FOG: GAF domain
NN2025.1524	SMU.440	SSA_0360	SGO_0266	Asp-tRNAAsn/Glu-tRNAGln amidotransferase A subunit and related amidases
NN2025.1579	SMU.371	SSA_2010	SGO_0399/2077	Predicted DNA alkylation repair enzyme
NN2025.1600	SMU.350	SSA_2122	SGO_0194	Flagellar GTP-binding_protein
NN2025.1607	SMU.343	SSA_1059	SGO_0816	Putative protein-S-isoprenylcysteine methyltransferase
NN2025.1671	SMU.296	SSA_0961	SGO_1074	Ketopantoate reductase
NN2025.1705	SMU.252	SSA_0584	SGO_0453	Leucyl-tRNA synthetase
